# Loss of TMEM65 in mice causes mitochondrial disease mediated by mitochondrial Ca^2+^

**DOI:** 10.1038/s41467-026-71761-w

**Published:** 2026-04-14

**Authors:** Yingfan Zhang, Hailey A. Parry, Laura Reyes, Alex Shamoun, Junhui Sun, Chengyu Liu, Danielle Springer, Audrey Noguchi, Sachiko Nakamori, Angel M. Aponte, Jeeva Munasinghe, Raul Covian, Elizabeth Murphy, Brian Glancy

**Affiliations:** 1 National Heart, Lung, and Blood Institute, National Institutes of Health, Bethesda, MD USA; 2 National Institute of Biomedical Imaging and Bioengineering, National Institutes of Health, Bethesda, MD USA; 3 National Institute of Neurological Disorders and Stroke, National Institutes of Health, Bethesda, MD USA; 4 National Institute of Arthritis and Musculoskeletal and Skin Diseases, National Institutes of Health, Bethesda, MD USA

**Keywords:** Energy metabolism, Energy metabolism, Ion channels in the nervous system

## Abstract

Transmembrane protein 65 (TMEM65) depletion in a patient caused severe mitochondrial encephalomyopathy, highlighting its clinical importance. Recent studies show TMEM65 acts as a mitochondrial Na^+^/Ca^2+^ exchanger in vitro. Here, we generated conditional *Tmem65* knockout mice to define its role in neuromuscular tissues in vivo. Both whole-body and nervous system–specific *Tmem65* knockouts exhibited severe growth retardation and seizure-associated sudden death at ~3 weeks, establishing TMEM65 as indispensable for neuronal function. Additionally, skeletal muscle–specific knockout produced adult-onset myopathy preceded by elevated mitochondrial Ca^2+^. Consistently, TMEM65 ablation caused loss of Na^+^-dependent mitochondrial Ca^2+^ export. Notably, blocking mitochondrial Ca^2+^ entry by mitochondrial calcium uniporter (MCU) knockout rescued the early lethality of whole-body *Tmem65* ablation, extending lifespan from ~3 weeks to >1 year. These data reveal an essential physiological role for TMEM65 and suggest that modulating mitochondrial Ca^2+^ may offer therapeutic value for TMEM65 misexpression and other mitochondrial diseases associated with Ca^2+^ overload.

## Abstract (web summary)

Loss of Tmem65 leads to neuromuscular defects due to loss of mitochondrial calcium export. Consistently, blocking mitochondrial calcium entry extends lifespan in Tmem65 knockout mice >10-fold highlighting potential calcium based therapies.

## Introduction

TMEM65 was first reported in MitoCarta, an inventory of more than 1000 mammalian mitochondrial proteins^1^, and initially considered to be localized to the inner mitochondrial membrane with three putative transmembrane domains, an N-terminus facing to the mitochondrial matrix, and a C-terminus in the mitochondrial intermembrane space^2^. TMEM65 is encoded by nuclear DNA, and contains a mitochondrial targeting sequence at the N-terminus of the protein so that it can be imported into mitochondria^2^. The expression of TMEM65 is regulated by the long non-coding RNA Steroid Receptor RNA Activator (SRA)^3^, which forms a ribonucleoprotein complex with leucine-rich pentatricopeptide repeat-containing protein (LRPPRC) and the Stem-Loop-Interacting RNA-binding Protein (SLIRP)^4^.

TMEM65 was previously suggested as a candidate mitochondrial Ca^2+^ uptake protein during the identification of the mitochondrial calcium uniporter (MCU)^5^. Indeed, the TMEM65 protein structure contains a glycine zipper motif^6^, which is a conserved structural component of transmembrane channel proteins^7^, strongly indicating it may form a mitochondrial channel. While TMEM65 has no known protein family members in vertebrates, orthology study of TMEM65 using OrthoDB v11^8^ shows that TMEM65 is an evolutionarily conserved protein, and many non-Metazoan orthologs contain a Ca^2+^ binding EF-hand domain (Supplementary Fig. 1). Recent studies show that heterologous expression of TMEM65 induces mitochondrial sodium-calcium exchange (mito-NCX) in cells lacking native mito-NCX activity, and purified, liposome-reconstituted TMEM65 exhibits mito-NCX features, providing strong evidence that TMEM65 is a mitochondrial Na/Ca exchanger^9^.

Here, we aimed to elucidate the function of TMEM65 in mammalian physiology by generating TMEM65 reporter mice as well as whole-body and tissue-specific TMEM65 knockout mice. Similar to the previous clinical report^10^, we find that mice completely lacking TMEM65 have a severe mitochondrial encephalomyopathy without cardiomyopathy. Additionally, we show that mice without TMEM65 throughout the whole body or specifically in neurons both display seizures and die at ~3 weeks of age indicating that neuronal TMEM65 is essential for life. Moreover, we establish that muscle-specific loss of TMEM65 is not critical for development, but rather leads to a progressive muscle wasting and metabolic uncoupling phenotype associated with impaired proteostasis and preceded by elevated mitochondrial Ca^2+^ levels. Consistent with concurrent reports^9,11,12^, we find that TMEM65 mediates mitochondrial Ca^2+^ by regulating Na^+^-dependent mitochondrial Ca^2+^ efflux. Finally, we demonstrate that rebalancing mitochondrial Ca^2+^ levels through removal of the major mitochondrial Ca^2+^ influx pathway (MCU) rescues the early lethality of whole body TMEM65 loss and results in mice that can breed and run similar to their wildtype counterparts. Thus, this work confirms the significant physiological role of TMEM65 in Na^+^-dependent mitochondrial Ca^2+^ efflux as well as identifies mitochondrial Ca^2+^ transport as a potential therapeutic target in TMEM65 associated mitochondrial disease.

## Results

### TMEM65 KO mice have mitochondrial disease

**Fig. 1:**
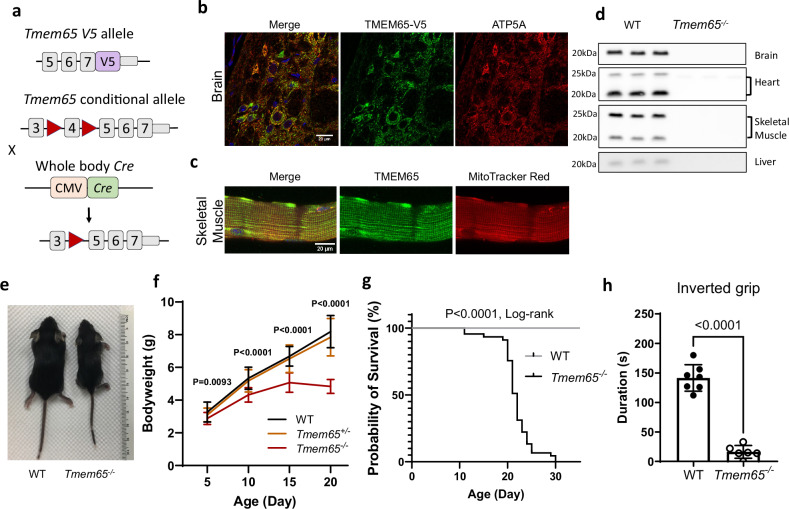
TMEM65 is a mitochondrial protein, and ablation of TMEM65 is lethal in mice. **a** Schematic of *Tmem65-V5* mouse, floxed mouse, and conditional KO mutant mice strategy. In *Tmem65*^*+V/+V*^ mice, *V5* tag (purple rectangle) was inserted before the stop codon in exon 7. In *Tmem65*^*fl/fl*^ mice *LoxP* sites(red triangles) flank exon 4. *Tmem65*^*fl/fl*^ were crossed with whole body Cre (CMV promoter) to generate *Tmem65*^*−/−*^. **b** TMEM65 colocalized with ATP5A, a mitochondrial complex V subunit in mouse brain section near lateral reticular nucleus. Pearson’s Coefficient r = 0.692. Representative section from 4 mice. **c** TMEM65 colocalized with MitoTracker Red in isolated mouse skeletal muscle. Pearson’s Coefficient r = 0.86. Representative image of 11 fibers from 2 mice. **d** Western blotting experiments show loss of TMEM65 in different tissues from P19 *Tmem65*^*−/−*^ mice. *n* = 3 replicates per group. **e** Representative image of a WT control and *Tmem65*^*−/−*^ littermate pups at P20. **f** Bodyweights of littermates measured at P5, P10, P15 and P20. WT (*Tmem65*^*+/+*^) *n* = 29; *Tmem65*^*+/−*^ *n* = 48; *Tmem65*^*−/−*^ *n* = 28 at 5, 10, and 15 -day-old, and *n* = 22 at 20-day-old. All data are presented as mean ± SD. One-way ANOVA were used for each age: *P* = 0.0093 at 5 days old, <0.0001 at 10 days old, <0.0001 at 15 days old, and <0.0001 at 20 days old. **g** Survival chart of WT controls (*n* = 31) and Tmem65^−/−^ littermates shows that Tmem65^−/−^ pups (*n* = 45) died between P11 and P30, with an average life span of 21.71 ± 3.57 days. Long-rank (Mantel–Cox) text was used. *P* < 0.0001. **h** Inverted grip test was done on weaning day of P21. Two-tailed *t* test was used. *P* < 0.0001. WT *n* = 7; *Tmem65*^*−/−*^ *n* = 6. Individual values as well as mean ± SD are presented.

To investigate the tissue and subcellular expression of TMEM65 protein, we generated a TMEM65-V5 in frame knock-in mouse model wherein a V5 tag was inserted to exon7 of *Tmem65* before the stop codon so that endogenous TMEM65-V5 instead of TMEM65 was expressed (*Tmem65*^*+V5/+V5*^, Fig. 1a). Western blots of TMEM65-V5 mouse tissues with 50 μg protein lysates indicated TMEM65 expression throughout the body with high expression in brain, heart, and soleus muscle (Supplementary Fig. 2a–e) with a molecular mass ~25 kDa for the full length TMEM65 protein, and/or ~20 kDa after cleavage of the mitochondrial targeting sequence^2^. Immunofluorescent analyses of TMEM65-V5 mouse tissue sections confirmed TMEM65 is localized primarily to mitochondria in brain (Fig. 1b and Supplementary Fig. 2f) and heart (Supplementary Fig. 3a–d). In wild-type mouse skeletal muscle, TMEM65 was expressed within the grid-like mitochondrial network^13^ surrounding the contractile machinery in both fixed, isolated soleus fibers (Fig. 1c) and in TMEM65-GFP-transfected Tibialis anterior (TA) muscle in vivo (Supplementary Fig. 2g). Therefore, TMEM65 is a mitochondrial protein highly expressed in excitable tissues.

To investigate the functional impact of TMEM65 in mice, we initially generated global TMEM65 KO mice with sgRNAs targeting exon1 and/or exon4 of *Tmem65* using standard CRISPR methodology (Supplementary Fig. 4a). However, almost all resultant KO mice had reduced body weight (Supplementary Fig. 4b) and died suddenly at 2-4 weeks of age precluding breeding. As a result, we generated a conditional loss-of-function mouse model wherein exon 4 of *Tmem65* was floxed by *LoxP* sites (*Tmem65*^*fl/fl*^, Fig. 1a) and we crossed these floxed mice with the CMV-Cre line^14^ to recreate TMEM65 whole-body KO mice. Western blotting results confirmed loss of TMEM65 in tissues from TMEM65 KO mice (Fig. 1d, Supplementary Fig. 12). TMEM65 KO mice (*Tmem65*^*−/−*^) were born at expected Mendelian ratios (Supplementary Table 1) but were growth retarded and showed growth regression after postnatal day (P) 15 (Fig. 1e, f). All *Tmem65*^*−/−*^ mice died between P11 and P30 with average life span of 21.7 ± 3.6 days (Fig. 1g) recapitulating the initial global TMEM65 KO phenotype. Epilepsy episodes were recorded with home cage monitoring and were always observed immediately prior to their sudden death (Supplementary Video 1). Weakness, occasional pulling/paralysis of hindlimb, and uncoordinated walking appeared with increasing age, culminating in death (Supplementary Table 2). Weakness of the *Tmem65*^*−/−*^ mice was also demonstrated by the lack of grip strength in an inverted grid test at P21 (Fig. 1h). Conversely, while the smaller hearts and lower heart rates were consistent with the growth delay^15^, no major cardiac structural or functional abnormalities were observed in the *Tmem65*^*−/−*^ mice (Supplementary Fig. 5). These data indicate an indispensable role for TMEM65 in mice and that the *Tmem65*^*−/−*^ mouse model recapitulates the mitochondrial encephalomyopathy without cardiomyopathy phenotype observed clinically^10^.

### Loss of TMEM65 in neurons is lethal

**Fig. 2:**
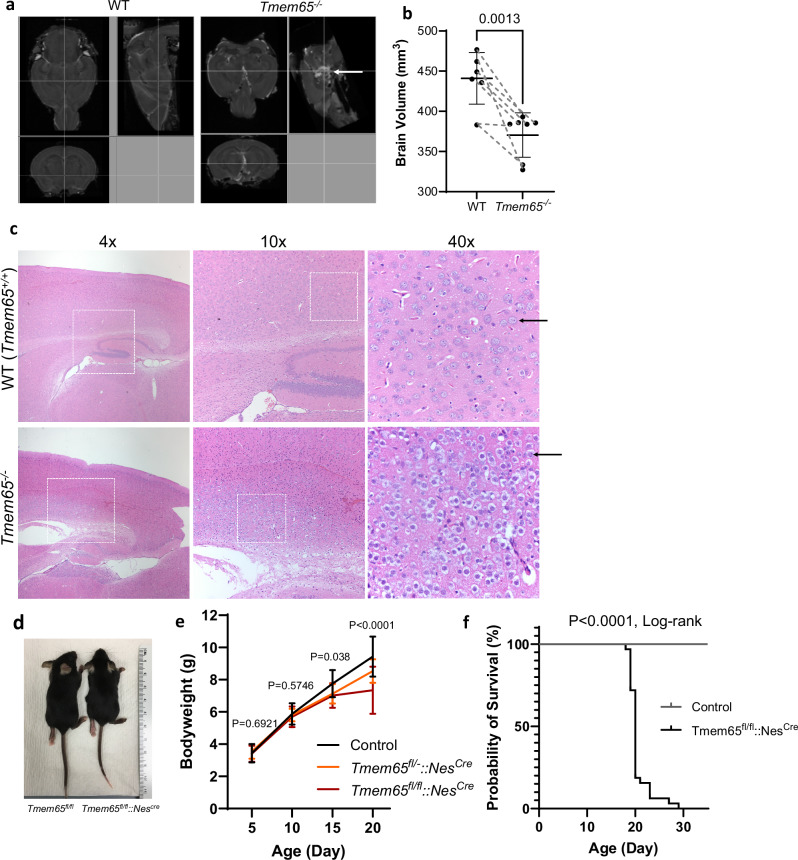
Loss of TMEM65 in brain causes neuronal vacuolar degeneration and sudden death. **a** MRI scan of a pair brains from P23 littermates show lesion and deformation of the *Tmem65*^*−/−*^ brain. White arrow indicates lesion in midbrain. **b** Mouse brain volumes measured with MRI scan. Dash line indicates littermate pairs. Two-tailed *t* test was used. WT *n* = 6; *Tmem65*^*−/−*^ *n* = 7. Individual values as well as mean ± SD are presented. **c** H&E staining of brain sagittal sections show diffusive neuronal vacuolar degeneration in the cingulate cortex region. Black arrows indicate normal and degenerated neurons in brain sections. **d** Representative image of *Tmem65*^*fl/fl*^ control and *Tmem65*^*fl/fl*^*::Nes*^*Cre*^ littermates at P20. **e** Body weights of littermates measured at P5, P10, P15 and P20. Control (*Tmem65*^*fl*^ or *Tmem65*^*fl/fl*^) *n* = 36 at 5-day-old, *n* = 28 at 10-day-old, *n* = 25 at 15-day-old, and *n* = 31 at 20-day-old; *Tmem65*^*fl*^*::Nes*^*Cre*^ *n* = 22 at 5-day-old, *n* = 21 at 10-day-old, *n* = 19 at 15-day-old, and *n* = 13 at 20-day-old. All data are presented as mean ± SD. One-way ANOVA was used for each age: *P* = 0.6921 at 5 days old, 0.5746 at 10 days old, 0.038 at 15 days old, and <0.0001 at 20 days old. **f** Survival chart of controls (*n* = 17) and *Tmem65*^*flox/flox*^*::Nes*^*Cre*^ pups (*n* = 32). Long-rank (Mantel–Cox) text was used. *P* < 0.0001.

Based on the high TMEM65 expression in the brain, seizures preceding death in TMEM65 KO mice, and the reported clinical phenotype^10^, we hypothesized that the primary functional impact of whole-body TMEM65 loss was on the brain. Indeed, magnetic resonance imaging (MRI) of P20–23 brains from *Tmem65*^*+/+*^ and *Tmem65*^*−/−*^ littermates revealed lesions and a microcephaly phenotype in TMEM65 KO mice with the most apparent volume loss in the cerebral cortex and midbrain (Fig. 2a, b), consistent with a pathological loss of neurons^16^. Additionally, neuronal vacuolar degeneration accompanied by nuclear shrinkage and condensation was observed in the cingulate cortex (Fig. 2c) and brain stem (Supplementary Fig. 6) on H&E stained sagittal sections. Interestingly, neuronal vacuolar degeneration has been previously reported in Leigh syndrome, another mitochondrial encephalomyopathy often accompanied by seizures^17^, and thus may be related to the phenotypes associated with TMEM65 KO.

To test whether loss of TMEM65 in the brain causes the sudden death observed in whole-body TMEM65 KO mice, TMEM65 was specifically ablated in the mouse nervous system by crossing the *Tmem65*^*fl/fl*^ mouse with the Nestin-Cre mouse line^18^, in which Cre recombinase expression starts at embryonic day (E) 12.5 in the central and peripheral nervous system^19^. *Tmem65*^*fl/fl*^*::Nes*^*Cre*^ mice recapitulated the epilepsy and sudden death in *Tmem65*^*−/−*^ mice as all died between P18-P29 with an average life span of 20.5 ± 2.3 days (Fig. 2f) and also developed growth delays starting at P15 (Fig. 2d, e). The phenotypic similarity between the whole-body KO *Tmem65*^*−/−*^ mice and nervous system-specific KO *Tmem65*^*fl/fl*^*::Nes*^*Cre*^ mice confirms that TMEM65 is indispensable for normal brain structure and function.

### TMEM65 mediates adult-onset muscle loss

**Fig. 3:**
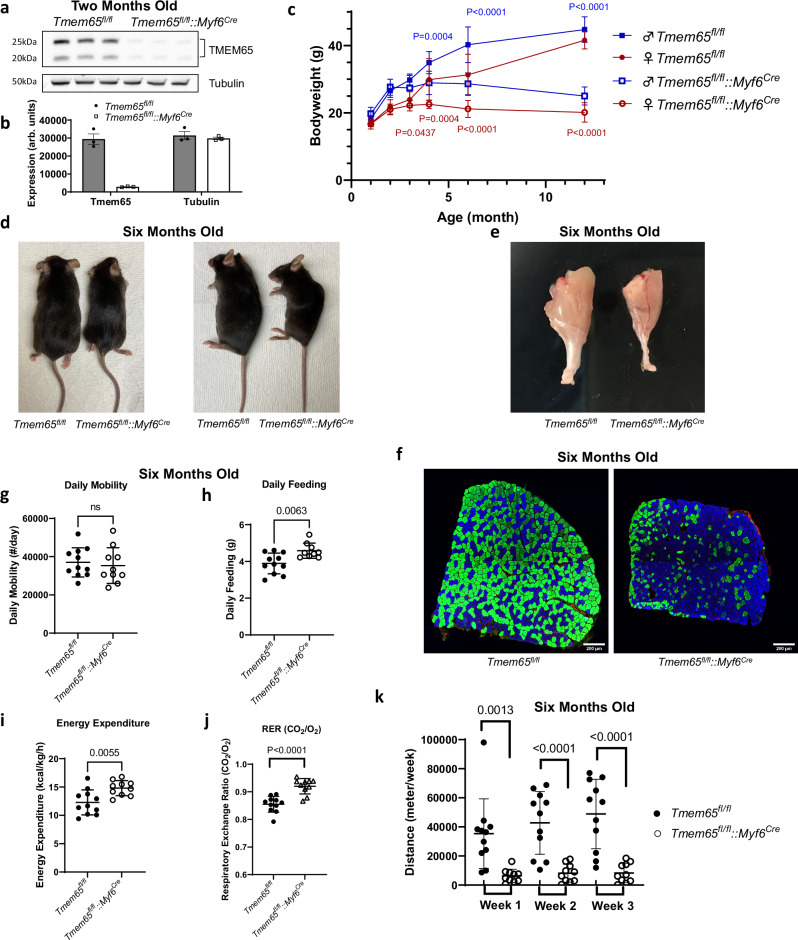
Loss of TMEM65 in skeletal muscle affects metabolism and leads to muscular atrophy and body weight loss. **a**, **b** Western blots of soleus muscles from 2 months old *Tmem65*^*fl/fl*^ and *Tmem65*^*fl/fl*^*::Myf6*^*Cre*^ littermates. *n* = 3 replicates for each groups. **c** Bodyweights of male and female mice at different ages. Female (red) *Tmem65*^*fl/fl*^ *n* = 19, 20, 10, 12, 24, and 5 at 1, 2, 3, 4, 6 and 12-month-old; female *Tmem65*^*fl/fl*^*::Myf6*^*Cre*^ *n* = 22, 18, 14, 13, 30, and 10 at 1, 2, 3, 4, 6 and 12-month-old; male *Tmem65*^*fl/fl*^ *n* = 18, 13, 8, 9, 9, and 8 at 1, 2, 3, 4, 6 and 12-month-old; male (blue) *Tmem65*^*fl/fl*^*::Myf6*^*Cre*^ *n* = 11, 11, 13, 14, 11, and 11 at 1, 2, 3, 4, 6 and 12-month-old respectively. All data are presented as mean ± SD. Female two-tailed *t* test: *P* = 0.6611 at 1-month-old, 0.2067 at 2-month-old, 0.0437 at 3-month-old, 0.0004 at 4-month-old, <0.0001 at 6-month-old, and <0.0001 at 12-month-old. Male two-tailed *t* test: *P* = 0.0553 at 1-month-old, 0.2755 at 2-month-old, 0.1569 at 3-month-old, 0.0004 at 4-month-old, <0.0001 at 6-month-old, and <0.0001 at 12-month-old. **d** Representative images of a pair of *Tmem65*^*fl/fl*^ and *Tmem65*^*fl/fl*^::*Myf6*^*Cre*^ littermates at 6-month-old. Side view of the mice shows hunched body posture of *Tmem65*^*fl/fl*^::*Myf6*^*Cre*^ mouse. **e** Gastrocnemius muscles from 6-month-old *Tmem65*^*fl/fl*^ and *Tmem65*^*fl/fl*^::*Myf6*^*Cre*^ littermates. **f** Immunofluorescence of soleus muscles from *Tmem65*^*fl/fl*^ and *Tmem65*^*fl/fl*^::*Myf6*^*Cre*^ littermates at 6-month-old. Myosin isoforms were identified with specific antibodies to muscle type I (blue), IIa (green) and IIb (red). **g**–**j** Metabolic parameters results from 6-month-old control *Tmem65*^*fl/fl*^ and *Tmem65*^*fl/fl*^::*Myf6*^*Cre*^ littermates collected in metabolic cages (CLAMS) for simultaneous measurement. *Tmem65*^*fl/fl*^ *n* = 11; *Tmem65*^*fl/fl*^::*Myf6*^*Cre*^ *n* = 10. Two-tailed *t* test was used. Individual value as well as mean ± SD are presented. ns not significant. *P* = 0.6552 for daily mobility, 0.0063 for daily feeding, 0.0055 for energy expenditure, and <0.0001 for RER. **k** Voluntary wheel running tests of 6-month-old control *Tmem65*^*fl/fl*^ and *Tmem65*^*fl/fl*^::*Myf6*^*Cre*^ littermates collected in individual housing for 3 weeks. *Tmem65*^*fl/fl*^ *n* = 11; *Tmem65*^*fl/fl*^::*Myf6*^*Cre*^ *n* = 10. Two-tailed *t* test was used. *P* = 0.0013 for week 1, *P* < 0.0001 for week 2, and *P* < 0.0001 for week3. Individual value as well as mean ± SD are presented.

Gross motor defects and muscle weakness, as described above for the whole-body TMEM65 KO mice, are most commonly attributable to dysfunctional skeletal muscle, in addition to brain^20,21^. To assess whether loss of TMEM65 in skeletal muscle accounts for the gross motor defects in whole-body TMEM65 KO mice, we generated skeletal muscle-specific TMEM65 KO mice by crossing *Tmem65*^*fl/fl*^ mice with Myf6-Cre mice where Cre expression is restricted to skeletal muscle starting in late gestation embryos^22–24^. The resultant *Tmem65*^*fl/fl*^*::Myf6*^*Cre*^ mice lacking TMEM65 in skeletal muscle (Fig. 3a, b) are viable, fertile, and maintain normal bodyweight (Fig. 3c) and muscle fiber-type distribution (Supplementary Fig. 7a, c) at 2 months of age. Thus, loss of TMEM65 in skeletal muscle alone does not account for the early-onset motor dysfunction in whole-body TMEM65 KO mice.

Despite the lack of overt phenotypes at 2 months old, both male and female muscle-specific TMEM65 KO mice begin to show growth delays by 3 months of age and regression after 4 months (Fig. 3b, c). This suggests that while TMEM65 may not be essential for muscle development, TMEM65 is critical for muscle maintenance in adulthood. By 6 months, *Tmem65*^*fl/fl*^*::Myf6*^*Cre*^ mice have significantly lower body weight (Fig. 3c, d), muscle mass (Fig. 3e), and muscle fiber cross-sectional area (CSA) in both soleus and flexor digitorum brevis (FDB) muscles (Supplementary Figs. 7a,b, 8a, b) compared to littermate controls (*Tmem65*^*fl/fl*^). Weight and muscle loss, frequently accompanied by hunched body posture (kyphosis, Fig. 3c right), continued through one year of age (Fig. 3c, Supplementary Fig. 7a, b, e) at which point mice were euthanized due to their emaciated nature. In slow-twitch soleus muscle, adult-onset muscle contractile type switching is prevalent in *Tmem65*^*fl/fl*^*::Myf6*^*Cre*^ mice as evident by the loss of muscle fibers expressing the fast-twitch myosin IIa isoform together with an increase in slow-twitch type I myosin-expressing fibers at 6 and 10 months (Fig. 3f, Supplementary Figs. 7a, c, 8a, b). This preferential loss of type IIa muscle fibers is consistent with the relatively greater expression of TMEM65 in this high mitochondrial content fiber type in mice^25^. Consistently, muscle-specific loss of TMEM65 also leads to an increase in type I fibers in the fast-twitch FDB muscle at 6 months (Supplementary Fig. 8a, c). These data demonstrate that persistent loss of TMEM65 in skeletal muscle leads to fast-to-slow fiber-type switching as well as muscle atrophy.

To evaluate the functional impact of muscle-specific TMEM65 KO, we assessed whole body metabolism and exercise activity in 6-month-old muscle-specific TMEM65 KO mice (*Tmem65*^*fl/fl*^*::Myf6*^*Cre*^) and littermate controls (*Tmem65*^*fl/fl*^). *Tmem65*^*fl/fl*^*::Myf6*^*Cre*^ mice and controls were similarly active when housed in metabolic cages (Fig. 3g). However, daily feeding, energy expenditure and respiratory exchange ratio (RER) were higher in *Tmem65*^*fl/fl*^*::Myf6*^*Cre*^ mice compared to controls (Fig. 3h–j, Supplementary Fig. 7d), indicating a reduced metabolic efficiency and greater reliance on carbohydrate metabolism. Further, when given access to a voluntary running wheel for three weeks, *Tmem65*^*fl/fl*^*::Myf6*^*Cre*^ mice ran less than 20% of their littermate controls each week (Fig. 3k). The reduction in metabolic efficiency, increased feeding, and lack of exercise performance combined with the loss of both fat and lean mass (Supplementary Fig. 7e) are all consistent with a metabolic uncoupling phenotype^26^ in mice lacking skeletal muscle TMEM65.

**Fig. 4:**
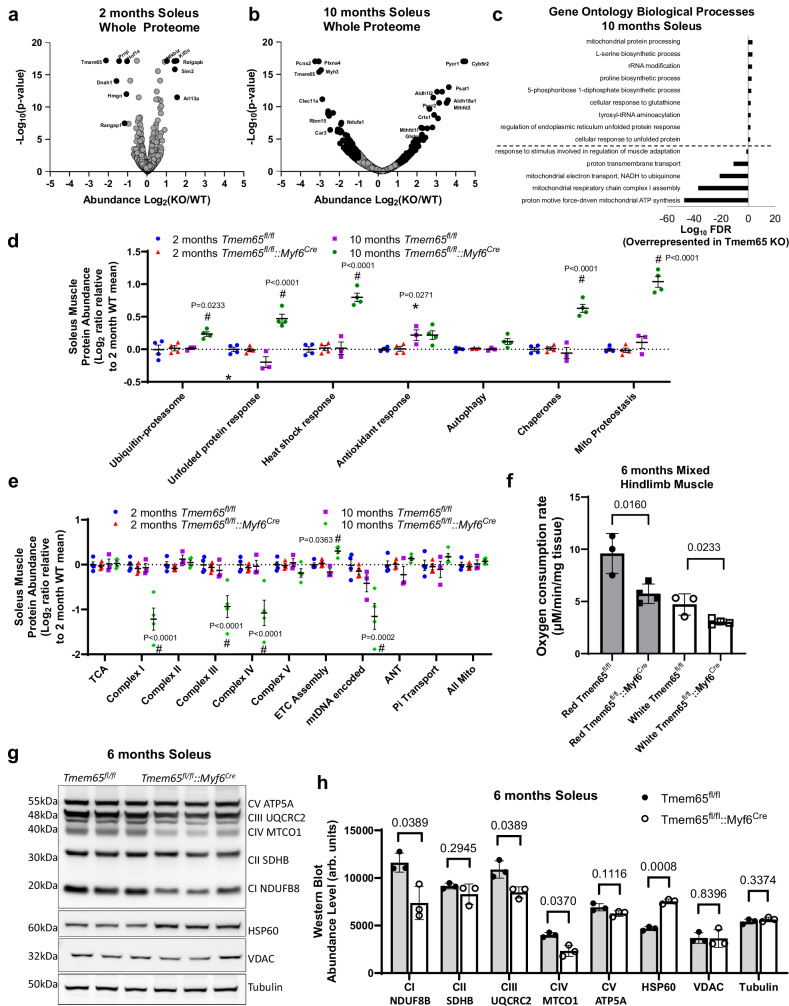
Proteomic analysis of muscle-specific TMEM65 KO and control muscles. **a**, **b** Volcano plots of protein abundances for 2-month-old and 10-month-old TMEM65 KO soleus muscles relative to controls. **c** Gene ontological process changes in 10-month-old soleus muscles *Tmem65*^*fl/fl*^ *n* = 3; *Tmem65*^*fl/fl*^*::Myf6*^*Cre*^ *n* = 4. **d** Abundances of proteins in proteostasis pathways were not changed at 2 months old. At 10 months old, major changes in these pathways occurred. * Significantly different from 2 month control; # Significantly different from 10 month control. For 2-month-old *Tmem65*^*fl/fl*^ *n* = 4; *Tmem65*^*fl/fl*^*::Myf6*^*Cre*^ *n* = 4. For 10-month-old *Tmem65*^*fl/fl*^ *n* = 3; *Tmem65*^*fl/fl*^*::Myf6*^*Cre*^ *n* = 4. Two-way ANOVAs with a Tukey multiple comparisons correction were used. Individual value as well as mean ± SEM are presented. *P* = 0.0233 for ubiquitin-proteasome; <0.0001 for unfolded protein response, <0.0001 for heat shock response, 0.0271 for antioxidant response, <0.0001 for chaperones, and <0.0001 for mito proteostasis. **e** Mitochondrial content proteins at 2-month-old and 10-month-old. For 2-month**-**old *Tmem65*^*fl/fl*^ *n* = 4; *Tmem65*^*fl/fl*^*::Myf6*^*Cre*^ *n* = 4. For 10-mo*n*th-old *Tmem65*^*fl/fl*^ *n* = 3; *Tmem65*^*fl/fl*^*::Myf6*^*Cre*^ *n* = 4. Two-way ANOVAs with a Tukey multiple comparisons correction were used. Individual value as well as mean ± SEM are presented. *P* < 0.0001 for Complex I, <0.0001 for complex III, <0.0001 for Complex IV, 0.0363 for ETC assembly, and 0.0002 for mtDNA encoded. **f** Oxygen consumption rates of frozen tissue lysates from red and white hindlimb muscles of 6-month-old. Red *Tmem65*^*fl/fl*^ *n* = 3; red *Tmem65*^*fl/fl*^*::Myf6*^*Cre*^ *n* = 4. White *Tmem65*^*fl/fl*^ *n* = 3; white *Tmem65*^*fl/fl*^*::Myf6*^*Cre*^ *n* = 4. Two-tailed *t* test was used. Individual value as well as mean ± SD are presented. **g** Western blots of 6-month-old muscle-specific TMEM65 KO and control soleus. **h** Quantification of protein expression from Western blots of (**g**), *n* = 3 replicates for each group. Two-tailed *t* test was used. Individual value as well as mean ± SD are presented. *P* = 0.0389 for Complex I, 0.2945 for Complex II, 0.0389 for Complex III, 0.0370 for Complex IV, 0.1116 for Complex V, 0.0008 for HSP60, 0.8396 for VDAC, and 0.3374 for tubulin.

To better understand the underlying cause of the adult-onset muscle wasting and metabolic uncoupling phenotypes in mice lacking TMEM65 in muscle, we evaluated the muscle proteome using tandem mass tag (TMT) mass spectrometry on soleus muscles from *Tmem65*^*fl/fl*^*::Myf6*^*Cre*^ mice and their littermate controls at 2 months and 10 months of age. Of the 4524 proteins identified across all 15 samples (*n* = 4 for 2 months control and KO and 10 months KO, *n* = 3 for 10 months control, Supplementary Dataset 1), only 11 proteins had abundances significantly altered by more than two-fold in the 2-month knockout muscles (Fig. 4a), and no overrepresented Gene Ontology biological processes were identified from these 11 proteins. However, at 10 months, 313 proteins were significantly altered by more than two-fold (162 up-regulated, 153 down-regulated, Fig. 4b). Gene Ontology analyses normalized for the whole proteome background identified protein and amino acid processing as the major up-regulated pathways whereas mitochondrial oxidative phosphorylation pathways were largely down-regulated (Fig. 4c). Further investigation into several different proteostasis pathways^27–33^ (Fig. 4d) revealed that proteins involved in the ubiquitin-proteasome, unfolded protein response, chaperones, heat shock response, and mitochondrial proteostasis were all up-regulated in *Tmem65*^*fl/fl*^*::Myf6*^*Cre*^ mice at 10 months. Altered proteostasis appears to occur prior to 10 months in *Tmem65*^*fl/fl*^*::Myf6*^*Cre*^ mice as mitochondrial heat shock protein 60 (Hsp60) abundance is also up-regulated at 6 months in soleus muscles (Fig. 4g, h). Stress induced by impaired mitochondrial proteostasis has resulted in reduced muscle mass across several different models^34–36^ and can also cause metabolic uncoupling^35^ and a loss of type IIa muscle fibers^36^. Thus, altered proteostasis provides a likely explanation for the major adult-onset phenotypes observed in the *Tmem65*^*fl/fl*^*::Myf6*^*Cre*^ mice.

Deeper exploration into the mitochondrial proteome (Fig. 4e) showed that although overall mitochondrial content (i.e. combined abundance of all mitochondrial proteins) was unchanged across groups, there was a specific loss of electron transport chain (ETC) Complexes I, III, and IV in the 10-month *Tmem65*^*fl/fl*^*::Myf6*^*Cre*^ muscles whereas Complexes II and V as well as the tricarboxylic acid (TCA) cycle were unchanged. Consistent with these proteomic results, Complexes I, III, and IV but not Complexes II and V were also down-regulated in 6-month-old *Tmem65*^*fl/fl*^*::Myf6*^*Cre*^ soleus muscles as evaluated by Western blot (Fig. 4g, h). To determine whether the loss of ETC proteins resulted in reduced energetic function in muscles lacking TMEM65, we evaluated the combined capacity of Complexes I + III + IV (NADH → O_2_) in 6-month-old muscles using a recently developed method for frozen tissues^37^. In order to minimize potential artifacts caused by the fiber-type switching reported above, hindlimb muscles were separated into red (more oxidative) and white (more glycolytic) portions. As expected, oxygen consumption rates were greater in red compared to white muscles for both groups (Fig. 4f). However, muscles lacking TMEM65 in both muscle types had reduced ETC capacity (Fig. 4f), consistent with the loss of protein abundance (Fig. 4c, e, g) as well as previous data from muscle biopsies and primary fibroblasts from a patient with loss of Tmem65 activity^10^. As a result, in order to meet the same ATP demands as their wildtype counterparts, 6-month-old *Tmem65*^*fl/fl*^*::Myf6*^*Cre*^ mice would require greater rates of fuel utilization in order to provide a larger NADH input into a lower activity ETC. Thus, the loss of ETC activity likely contributes to the increased feeding (Fig. 3h) observed in 6-month-old *Tmem65*^*fl/fl*^*::Myf6*^*Cre*^ mice.

### TMEM65 regulates mitochondrial calcium efflux

**Fig. 5:**
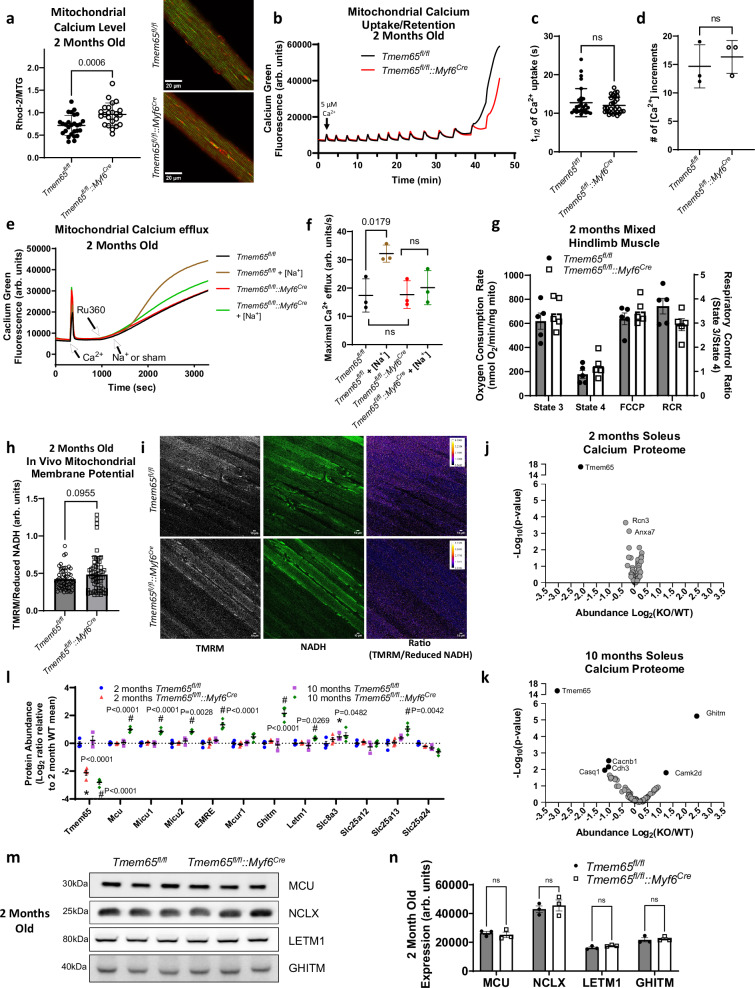
TMEM65 regulates mitochondrial calcium release in Na^+^ dependent manner. **a** Relative levels of mitochondrial matrix calcium in FDB muscle from control *Tmem65*^*fl/fl*^ (*n* = 27 cells from 3 mice) and *Tmem65*^*fl/fl*^*::Myf6*^*Cre*^ (*n* = 23 cells from 3 mice) at 2 months old as measured by the fluorescence ratio of Rhod-2 to MitoTracker Green. Two-tailed *t* test was used. Individual values as well as mean ± SD are presented. Representative images of *Tmem65*^*fl/fl*^ and *Tmem65*^*fl/fl*^*::Myf6*^*Cre*^ FDBs are shown on the right. **b** Representative mitochondrial calcium uptake with sequential Ca^2+^ additions (5 µM) to isolated skeletal muscle mitochondria (100 µg) of *Tmem65*^*fl/fl*^ and *Tmem65*^*fl/fl*^*::Myf6*^*Cre*^ mice. Arrow indicates the first increment of 5 μM Ca^2+^. **c** Mitochondrial calcium uptake half time (t_1/2_) was similar between *Tmem65*^*fl/fl*^ and *Tmem65*^*fl/fl*^*::Myf6*^*Cre*^ samples. *n* = 30 for each group. Two-tailed *t* test was used. Individual value as well as mean ± SD are presented. ns not significant. **d** Summary of the mitochondrial calcium uptake capacity of isolated skeletal mitochondria from *Tmem65*^*fl/fl*^ and *Tmem65*^*fl/fl*^*::Myf6*^*Cre*^ mice. *n* = 3 mice. Two-tailed *t* test was used. Individual value as well as mean ± SD are presented. ns not significant. **e** Representative traces of mitochondrial calcium efflux assay. A bolus of Ca^2+^ (50 µM), MCU inhibitor, Ru360 (3 µM), and NaCl (20 mM) or sham were added sequentially to isolated skeletal muscle mitochondria (100 µg/well) incubated with Calcium Green. **f** Summary of the maximal rates of mitochondrial Ca^2+^ efflux induced by Ru360 alone, or Ru360 and Na^+^ in *Tmem65*^*fl/fl*^ control or *Tmem65*^*fl/fl*^*::Myf6*^*Cre*^ mitochondria. *n* = 3 replicates per group. Two-tailed t test was used. Individual value as well as mean ± SD are presented. **g** Skeletal muscle mitochondria functions. Isolated mitochondria from 2-month-old mice were given glutamate, malate and pyruvate as fuel. At 2 months of age, no difference was observed in state 3 and state 4 oxygen consumption rates, and respiratory control ratio (RCR) and FCCP maximum rate is similar between *Tmem65*^*fl/fl*^ and *Tmem65*^*fl/fl*^*::Myf6*^*Cre*^*. Tmem65*^*fl/fl*^ *n* = 5; *Tmem65*^*fl/fl*^*::Myf6*^*Cre*^ *n* = 5. Two-tailed *t* test was used. Individual value as well as mean ± SEM are presented. **h** Mitochondrial membrane potential was measured in vivo in *Tmem65*^*fl/fl*^ and *Tmem65*^*fl/fl*^*::Myf6*^*Cre*^ mice at 2 months old. For *Tmem65*^*fl/fl*^ *n* = 57 from 8 mice; for *Tmem65*^*fl/fl*^*::Myf6*^*Cre*^ *n* = 63 from 9 mice. Two-tailed *t* test was used. Individual value as well as mean ± SEM are presented. **i** Representative images of in vivo muscle fibers. **j**, **k** Volcano plots of calcium-related protein abundances for 2-month-old and 10-month-old TMEM65 KO soleus muscles relative to controls, respectively. **l** Mass spectrometry results of Soleus muscles from 2-month-old and 10-month-old *Tmem65*^*fl/fl*^ or *Tmem65*^*fl/fl*^*::Myf6*^*Cre*^ mice show that protein expression levels of detectable mitochondrial calcium transport channels, carriers, and regulators were not affected in 2-month-old *Tmem65*^*fl/fl*^*::Myf6*^*Cre*^ soleus. Two-way ANOVA was used. * Significantly different from 2 month WT; # Significantly different from 10 month WT. For 2-month-old *Tmem65*^*fl/fl*^ *n* = 4; *Tmem65*^*fl/fl*^*::Myf6*^*Cre*^ *n* = 4. For 10-month-old *Tmem65*^*fl/fl*^ *n* = 3; *Tmem65*^*fl/fl*^*::Myf6*^*Cre*^ *n* = 4. Individual value as well as mean ± SEM are presented. *P* < 0.0001 for *Tmem65*, <0.0001 for *Mcu*, <0.0001 for *Micu1*, 0.0028 for *Micu2*, <0.0001 for *Mcur1*, <0.0001 for *Ghitm*, 0.0269 for *Letm1*, 0.0482 for *Slc8a3*, and 0.0042 for *Slc25a13*. **m** Western blots of mitochondrial calcium import and export proteins in 2-month-old Soleus muscles from *Tmem65*^*fl/fl*^ and *Tmem65*^*fl/fl*^*::Myf6*^*Cre*^ mice. **n** Quantification of (**m**) (*n* = 3 replicates per group). Two-tailed *t* test was used. Individual value as well as mean ± SD are presented. ns not significant.

Seizures^38^, gross motor defects^38^, apoptosis^38,39^, muscle atrophy^38^, and metabolic uncoupling phenotypes^39^, as described here for the TMEM65 KO mice, have all previously been linked to mitochondrial Ca^2+^ handling. TMEM65 was reported to regulate mitochondrial Ca^2+^ efflux in cell lines^9,11,12^, and a recent paper demonstrated TMEM65 as a mitochondrial Na^+^/Ca^2+^ exchanger in vitro^9^. To evaluate the role of TMEM65 in mitochondrial Ca^2+^ handling in mammalian tissues in vivo, we used the 2-month-old *Tmem65*^*fl/fl*^*::Myf6*^*Cre*^ mice, which display no overt phenotype, in an attempt to identify primary mechanisms rather than secondary effects involved after the onset of systemic neuromuscular pathologies observed in the whole body, neuron-specific, and older muscle-specific TMEM65 KO mice. Mitochondrial Ca^2+^ levels in isolated FDB muscle fibers, assessed by the fluorescence of mitochondrial calcium indicator Rhod-2 normalized to a mitochondrial content marker, MitoTracker Green, were higher in 2-month-old *Tmem65*^*fl/fl*^*::Myf6*^*Cre*^ mice compared to littermate controls (Fig. 5a). To determine whether altered Ca^2+^ uptake or retention could explain the increased mitochondrial Ca^2+^ levels in *Tmem65*^*fl/fl*^*::Myf6*^*Cre*^ mice, we gave successive 5 µM additions of Ca^2+^ to isolated hindlimb muscle mitochondria in the presence of extramitochondrial Ca^2+^ indicator, Calcium Green (Fig. 5b). Both Ca^2+^ uptake and retention capacity were similar between mitochondria from *Tmem65*^*fl/fl*^*::Myf6*^*Cre*^ mice and littermate controls (Fig. 5c, d) indicating that altered Ca^2+^ uptake and retention do not explain the increased mitochondrial Ca^2+^ levels in *Tmem65*^*fl/fl*^*::Myf6*^*Cre*^ mouse muscles. Next, we loaded mitochondria with a 50 µM bolus of Ca^2+^ and then inhibited mitochondrial Ca^2+^ uptake with Ru360, a MCU inhibitor, in order to assess the rate of Ca^2+^ efflux (Fig. 5e). While the basal rate of mitochondrial Ca^2+^ efflux was no different between *Tmem65 *^*fl/fl*^*::Myf6*^*Cre*^ mitochondria and controls, addition of Na^+^ was unable to stimulate increased Ca^2+^ efflux in *Tmem65*^*fl/fl*^*::Myf6*^*Cre*^ mitochondria as it was in controls (Fig. 5f). Similarly, Na^+^-dependent mitochondrial Ca^2+^ efflux is also reduced in brain mitochondria from 19 day old whole body TMEM65 KO mice (Supplementary Fig. 9). The loss of Na^+^-dependent mitochondrial Ca^2+^ efflux could explain why mitochondrial Ca^2+^ levels are higher in *Tmem65*^*fl/fl*^*::Myf6*^*Cre*^ mice. However, because mitochondrial Ca^2+^ exchange is electrogenic, Ca^2+^ levels are also dependent on the mitochondrial membrane potential in addition to the activities of the mitochondrial Ca^2+^ transport machinery. Activity of the oxidative phosphorylation pathway, which generates and utilizes the mitochondrial membrane potential to make ATP, was not different in isolated mitochondria from 2-month-old *Tmem65*^*fl/fl*^*::Myf6*^*Cre*^ mice and littermate controls (Fig. 5g), consistent with the lack of differences in oxidative phosphorylation protein abundances at this time point (Fig. 4e). Additionally, we assessed the muscle mitochondrial membrane potential of 2-month-old *Tmem65*^*fl/fl*^*::Myf6*^*Cre*^ mice and littermate controls in vivo (Fig. 5h, i), in isolated muscle fibers (Supplementary Fig. 10a, b), and in isolated mitochondria (Supplementary 10c, d). The mitochondrial membrane potential was no different between *Tmem65*^*fl/fl*^*::Myf6*^*Cre*^ mice and controls using each of these three different methods suggesting that an altered voltage across the mitochondrial inner membrane was not contributing to the increased mitochondrial Ca^2+^ levels in *Tmem65*^*fl/fl*^*::Myf6*^*Cre*^ mice. Together, these data indicate that removal of TMEM65 in vivo, just as in cell lines^11,12^, leads to increased mitochondrial Ca^2+^ levels as a result of the loss of Na^+^-dependent mitochondrial Ca^2+^ efflux.

To determine whether altered expression of any other known Ca^2+^ handling proteins as a result of TMEM65 loss may explain the increased mitochondrial Ca^2+^ levels and loss of sodium-dependent efflux, we filtered our soleus muscle proteomic results (Fig. 4) for known Ca^2+^ related proteins. The abundance of several Ca^2+^ handling proteins was altered in the 10-month-old *Tmem65*^*fl/fl*^*::Myf6*^*Cre*^ muscles with the muscle wasting phenotype (Fig. 5k, l). Interestingly, the most up-regulated Ca^2+^ related protein at 10 months was GHITM (aka TMBIM5/MICS1) suggesting that non-Na^+^ dependent mitochondrial Ca^2+^ efflux^40,41^ may be up-regulated in these muscles to compensate for the loss of TMEM65 mediated Na^+^ dependent Ca^2+^ efflux. However, at 2 months, TMEM65 was the only Ca^2+^ related protein detected in our proteome with an altered abundance (Fig. 5j, l). The lack of change in protein abundances for MCU, GHITM, and LETM1 in 2-month *Tmem65*^*fl/fl*^*::Myf6*^*Cre*^ soleus muscles was additionally confirmed by Western blot (Fig. 5m, n). Western blotting also allowed us to show that NCLX abundance was unchanged with TMEM65 KO (Fig. 5m, n) since this protein was not detected by mass spectrometry. Thus, the increased mitochondrial Ca^2+^ levels and loss of Na^+^-dependent mitochondrial Ca^2+^ efflux observed in 2-month-old *Tmem65*^*fl/fl*^*::Myf6*^*Cre*^ muscles appears to be directly attributable to the loss of TMEM65.

Na^+^-dependent mitochondrial Ca^2+^ release has long been solely ascribed to the NCLX (SLC8B1/SLC24A6) protein^38,42,43^. However, NCLX abundance was not changed here in muscle-specific TMEM65 KO soleus at 2 months old (Fig. 5m, n) or in 19-day-old whole body TMEM65 KO brains when mitochondrial Na^+^-dependent Ca^2+^ release was lost. Additionally, a concurrent report^12^ demonstrated that a change in NCLX levels was not detected in TMEM65 knockdown (KD) mouse heart tissue, TMEM65 overexpression AC16 cardiomyocytes, or TMEM65 KD C2C12 myoblasts, and NCLX deletion in mouse embryonic fibroblasts resulted in no change in TMEM65 expression. Thus, the abundance of TMEM65 and NCLX does not necessarily track each other. To further evaluate whether TMEM65 interacts with NCLX in mammalian tissues in vivo, we performed co-immunoprecipitation (Co-IP) analyses. However, we first needed to verify that we had a valid anti-NCLX antibody. We generated a plasmid expressing NCLX-FLAG, in which a FLAG tag was added to the C terminus of NCLX protein and expressed NCLX-FLAG protein in HEK293T cells. We found that anti-NCLX antibody detected a major band of ~50 kDa in Western blotting (Supplementary Fig. 11a). Anti-FLAG antibody also detected the same band of ~50 kDa on the same blot, confirming this band of 50 kDa in Western blot was NCLX-FLAG, and this commercially available anti-NCLX antibody accurately detects NCLX by Western blot. Moreover, several others have validated this same NCLX antibody in NCLX KO or KD experiments^44–46^. Co-IP experiments were done using brain lysates from homozygous TMEM65-V5 mice (Supplementary Fig. 11b). With V5 antibody-conjugated beads, TMEM65-V5 was pulled down successfully. However, NCLX was not able to be pulled down with TMEM65-V5, again suggesting TMEM65 does not interact with NCLX directly. A lack of interaction between native TMEM65 and NCLX when both proteins were expressed with tags in HEK cells was also recently reported^9^, thereby offering additional support for our results. Additionally, when Co-IP experiments were performed using brain lysates from heterozygous TMEM65-V5 mice (*Tmem65*^*+V/+*^), both TMEM65-V5 protein and native TMEM65 protein were pulled down with V5 antibody-conjugated beads, strongly suggesting direct interaction between TMEM65 proteins to form a TMEM65 complex (Supplementary Fig. 11c), consistent with a recent report that TMEM65 forms a homodimer^9^. Indeed, purified TMEM65 reconstituted in liposomes in the absence of NCLX was recently shown to mediate Na^+^-dependent Ca^2+^ exchange^9^. Thus, our data offer further support that TMEM65 mediates mitochondrial Na^+^-dependent Ca^2+^ exchange independent of NCLX^9^.

### Rebalancing calcium restores lifespan

**Fig. 6:**
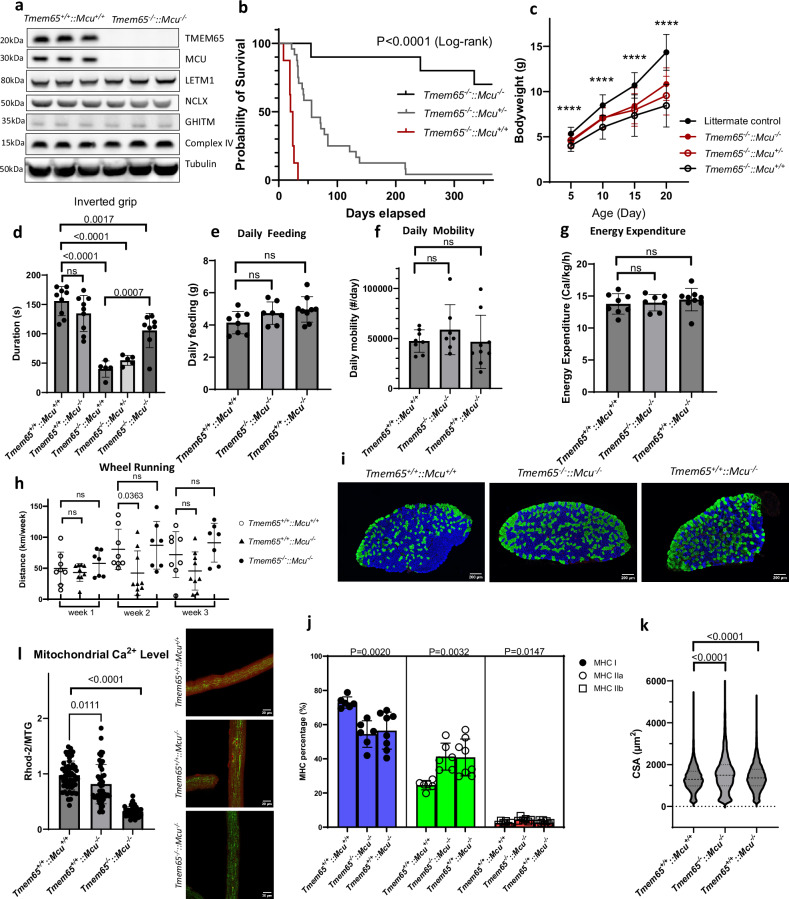
Removal of MCU rescues lethality of Tmem65^−/−^ mice. **a** Western blot analysis of brain tissues from P20 WT (*Tmem65*^*+/+*^*::Mcu*^*+/+*^) and *Tmem65*^*−/−*^*::Mcu*^*−/−*^ confirmed the loss of both TMEM65 and MCU in *Tmem65*^*−/−*^*::Mcu*^*−/−*^ mice. Expression level of other calcium-handing proteins does not change. *n* = 3 replicates per group. **b** Survival chart for *Tmem65*^*−/−*^*::Mcu*^*+/−*^ (*n* = 24), *Tmem65*^*−/−*^*::Mcu*^*−/−*^ (*n* = 10) and *Tmem65*^*−/−*^*::Mcu*^*+/+*^ (*n* = 8) mice. Log-rank (Mantel–Cox) test was used. *P* < 0.0001. **c** Bodyweights of control (*n* = 129, 114, 97 and 86 from 5 to 20-day-old), *Tmem65*^*−/−*^*::Mcu*^*+/+*^ (*n* = 5, 5, 4 and 4 from 5 to 20-day-old), *Tmem65*^*−/−*^*::Mcu*^*+/−*^ (*n* = 23, 25, 21 and 21 from 5 to 20day-old), and *Tmem65*^*−/−*^*::Mcu*^*−/−*^ (*n* = 6, 6, 5 and 6 at 5 to 20-day-old) mice. Data are represented as mean ± SD. One-way ANOVA test was used. ****P < 0.0001. **d** Inverted grip test was done on weaning day of P20-21on *Tmem65*^*+/+*^*::Mcu*^*+/+*^(*n* = 9), *Tmem65*^*+/+*^*::Mcu*^*−/−*^(*n* = 9), *Tmem65*^*−/−*^*::Mcu*^*+/+*^, (*n* = 5), *Tmem65*^*−/−*^*::Mcu*^*+/−*^ (*n* = 5) and *Tmem65*^*−/−*^*::Mcu*^*−/−*^(*n* = 8). Two-tailed *t* tests were used. *P* = 0.1297 for *Tmem65*^*+/+*^*::Mcu*^*+/+*^ vs *Tmem65*^*+/+*^*::Mcu*^*−/−*^, <0.0001 for *Tmem65*^*+/+*^*::Mcu*^*+/+*^ vs *Tmem65*^*−/−*^*::Mcu*^*+/+*^, <0.0001 for *Tmem65*^*+/+*^*::Mcu*^*+/+*^ vs *Tmem65*^*−/−*^*::Mcu*^*+/−*^, 0.0017 for *Tmem65*^*+/+*^*::Mcu*^*+/+*^ vs *Tmem65*^*−/−*^*::Mcu*^*−/−*^, and 0.0007 for *Tmem65*^*−/−*^*::Mcu*^*+/+*^ vs *Tmem65*^*−/−*^*::Mcu*^*−/−*^. Individual values as well as mean ± SD are presented. ns not significant. **e**–**g** Metabolic parameters from 3.5-month-old *Tmem65*^*+/+*^*::Mcu*^*+/+*^(*n* = 8), *Tmem65*^*+/+*^*::Mcu*^*−/−*^(*n* = 9) and *Tmem65*^*−/−*^*::Mcu*^*−/−*^ (*n* = 7) littermates collected with CLAMS. Two-tailed *t* tests were used. Individual value as well as mean ± SD are presented. ns not significant. **h** Voluntary wheel running tests of 4.5-month-old *Tmem65*^*+/+*^*::Mcu*^*+/+*^(*n* = 8), *Tmem65*^*+/+*^*::Mcu*^*−/−*^(*n* = 9), and *Tmem65*^*−/−*^*::Mcu*^*−/−*^ (*n *= 7) littermates collected in individual housing for 3 weeks. Two-tailed *t* test was used. Individual value as well as mean ± SD are presented. ns not significant. **i** Representative images of soleus muscle cross-sections from these mice. Blue: myosin type I, green: myosin type IIa, and red: myosin type IIb. Individual value as well as mean ± SD are presented. **j** Quantification of myosin isoform composition in control *Tmem65*^*+/+*^*::Mcu*^*+/+*^ (*n* = 6), *Tmem65*^*−/−*^*::Mcu*^*−/−*^(*n* = 6), and *Tmem65*^*+/+*^*::Mcu*^*−/−*^ (*n* = 8) soleus muscle cross sections at 6-month-old. One-way ANOVA test was used. Individual value as well as mean ± SD are presented. **k** Quantification of soleus muscle fiber cross section area. One-way ANOVA test *P* < 0.0001. Two-tailed *t* test *P* < 0.0001 for *Tmem65*^*+/+*^*::Mcu*^*+/+*^ vs *Tmem65*^*−/−*^*::Mcu*^*−/−*^, and <0.0001 for *Tmem65*^*+/+*^*::Mcu*^*+/+*^ vs *Tmem65*^*+/+*^*::Mcu*^*−/−*^. **l** Relative levels of mitochondrial matrix calcium in FDB muscle fibers from 2-month-old *Tmem65*^*+/+*^*::Mcu*^*+/+*^*, Tmem65*^*+/+*^*::Mcu*^*−/−*^, and *Tmem65*^*−/−*^*::Mcu*^*−/−*^ mice. *n* = 55, 47, and 49 respectively, from 3 mice in each group. Two-tailed *t* test was used. *P* = 0.0111 for *Tmem65*^*+/+*^*::Mcu*^*+/+*^ vs *Tmem65*^*+/+*^*::Mcu*^*−/−*^ and <0.0001 for *Tmem65*^*+/+*^*::Mcu*^*+/+*^ vs *Tmem65*^*−/−*^*::Mcu*^*−/−*^. Individual value as well as mean ± SD are presented.

Based on the observed mitochondrial Ca^2+^ overload and loss of mitochondrial Ca^2+^ export capacity in TMEM65 KO mice, we hypothesized that rebalancing mitochondrial Ca^2+^ levels by inhibiting mitochondrial Ca^2+^ uptake would rescue the lethality of TMEM65 KO. Thus, we crossed *Tmem65*^*−/−*^ mice with global MCU knockout mice^47^ (*Mcu*^*−/−*^), which led to complete loss of TMEM65 and MCU. LETM1, GHITM and mitochondrial complex IV expression levels were not changed, while NCLX expression level was slightly reduced in 20 day old brains of *Tmem65*^*−/−*^*::Mcu*^*−/−*^ mice (Fig. 6a). Whole body TMEM65 KO again led to death around 20 days of age (Fig. 6b) indicating that the mixed genetic background (C57BL/6 and CD1) of the *Tmem65*^*−/−*^*::Mcu*^*+/+*^ mice did not alter the phenotype of lethality. However, heterozygous MCU KO (*Tmem65*^*−/−*^*::Mcu*^*+/−*^) more than doubled the lifespan of TMEM65 KO mice while homozygous MCU KO (*Tmem65*^*−/−*^*::Mcu*^*−/−*^) further prolonged lifespan to longer than one year for most mice (Fig. 6b) despite not fully rescuing the delayed growth associated with TMEM65 KO (Fig. 6c). *Tmem65*^*−/−*^*::Mcu*^*−/−*^ mice were also fertile and capable of maintaining colonies. Loss of MCU also improved muscle function in *Tmem65*^*−/−*^ mice, as *Tmem65*^*−/−*^*::Mcu*^*−/−*^ mice have a prolonged inverted grip duration compared to *Tmem65*^*−/−*^*::Mcu*^*+/+*^ mice (Fig. 6d). These results demonstrate that the lethality and weakness associated with TMEM65 KO is mediated by mitochondrial Ca^2+^ handling.

To further evaluate the functional impact of the dual loss of MCU and TMEM65, we assessed whole body metabolism and exercise activity in 3.5-month-old *Tmem65*^*+/+*^*::Mcu*^*+/+*^, *Tmem65*^*+/+*^*::Mcu*^*−/−*^ and *Tmem65*^*−/−*^*::Mcu*^*−/−*^ mice. Daily feeding, daily mobility and energy expenditure were all comparable to control *Tmem65*^*+/+*^*::Mcu*^*+/+*^ mice (Fig. 6e–g), indicating that metabolic uncoupling did not occur in *Tmem65*^*−/−*^*::Mcu*^*−/−*^ mice. In a voluntary wheel running study, the running capacity of *Tmem65*^*−/−*^*::Mcu*^*−/−*^ mice was similar compared to *Tmem65*^*+/+*^*::Mcu*^*+/+*^ mice for three continuous weeks (Fig. 6h). While we observed reduced running capacity in *Tmem65*^*+/+*^*::Mcu*^*−/−*^ mice on the second week, consistent with previous reports that loss of MCU function affects exercise endurance in mice^47^, the running capacity of the double knockout *Tmem65*^*−/−*^*::Mcu*^*−/−*^ mice was similar to the wildtype *Tmem65*^*+/+*^*::Mcu*^*+/+*^ mice (Fig. 6h). These results show that removal of MCU also restores running capacity in TMEM65 KO mice.

To evaluate if MCU loss altered the fast-to-slow contractile type switch observed in *Tmem65*^*+/+*^*::Myf6*^*cre*^ mice, soleus muscles from 6-month-old mice were examined. Interestingly, both *Tmem65*^*−/−*^*::Mcu*^*−/−*^ and *Tmem65*^*+/+*^*::Mcu*^*−/−*^ mice underwent a slow-to-fast contractile type switch (Fig. 6i, k), which was opposite to the fast-to-slow switch in TMEM65 muscle-specific KO mice at the same age. CSAs of *Tmem65*^*−/−*^*::Mcu*^*−/−*^ and *Tmem65*^*+/+*^*::Mcu*^*−/−*^ soleus muscle were also increased compared to *Tmem65*^*+/+*^*::Mcu*^*+/+*^ control, probably attributed to the slow-to-fast muscle type switch. Finally, lower mitochondrial Ca^2+^ levels were confirmed in isolated FDB fibers from 2 months old *Tmem65*^*−/−*^*::Mcu*^*−/−*^ mice (Fig. 6l), indicating modulating Ca^2+^ handling in mitochondria plays significant role in rescuing TMEM65 KO phenotypes.

## Discussion

Tissue-specific variability in the expression of TMEM65 and other known mitochondrial Ca^2+^ import/export proteins^48–52^ highlights the complexity of mitochondrial Ca^2+^ handling within different parts of the body and points to the need for more comprehensive understanding of its regulatory mechanisms. The conditional TMEM65 KO mouse model developed here offers compelling evidence that loss of Na^+^-dependent mitochondrial Ca^2+^ export in the brain leads to a lethal encephalopathy phenotype. This broadens the existing framework of knowledge beyond the accelerated progression of Alzheimer’s disease and cognitive decline associated with the neuronal-specific deletion of the putative mitochondrial Na^+^-dependent Ca^2+^ exporter, NCLX^53,54^, and adds another dimension to the role of mitochondrial Ca^2+^ in neuronal health. In our hands, TMEM65 does not appear to interact with NCLX directly (Supplementary Fig. 11), and NCLX abundance is not correlated with the rate of Na^+^-dependent mitochondrial Ca^2+^efflux in both muscle (Fig. 5) and brain (Supplementary Fig. 10). These conclusions are similar to recent work reported by the DeStefani group^11^ where TMEM65 overexpression still leads to an increase in Na^+^-dependent mitochondrial Ca^2+^ efflux in cells where NCLX is knocked out, and heterologous expression of TMEM65 induces mitochondrial Na^+^/Ca^2+^ exchange in cells lacking native mito-NCX activity^9^. Further, purified TMEM65 reconstituted into liposomes was recently shown to facilitate Ca^2+^ uptake dependent on Na^+^ and Li^+^, and inhibited by CGP-37157^9^, all critical characteristics of the mitochondrial Na^+^/Ca^2+^ exchanger^55^. Thus, TMEM65 appears to be a mitochondrial Na^+^/Ca^2+^ exchanger on its own and independent of NCLX.

The adult-onset of the muscle atrophy and tissue remodeling phenotype upon loss of TMEM65 in skeletal muscle provides a unique window of time when the primary functions of TMEM65 can be probed without major influence from the pathological adaptations that occur with whole-body and neuronal-specific TMEM65 ablation. In this model, loss of TMEM65 results in greater accumulation of mitochondrial Ca^2+^ and a lack of Na^+^-stimulated mitochondrial Ca^2+^ efflux despite no alterations in the expression of known mitochondrial Ca^2+^ handling machinery. Our data corroborate recent work showing that mitochondrial Na^+^/Ca^2+^ exchange is activated when TMEM65 is overexpressed and blocked when TMEM65 is removed in vitro^11,12^. That rebalancing mitochondrial Ca^2+^ dynamics by removing MCU is able to reverse the lethality associated with loss of TMEM65 in the brain offers further support for the importance of TMEM65 in mediating mitochondrial Ca^2+^ export. Moreover. these results link disruption in mitochondrial Ca^2+^ homeostasis to severe mitochondrial disease and suggest inhibition of mitochondrial Ca^2+^ influx^56^ may be a potential therapeutic target for defects associated with TMEM65 loss of function^10^ or other diseases characterized by mitochondrial Ca^2+^ overload^53,54,57,58^.

## Methods

### Animal studies

All mouse procedures were performed in accordance with institutional guidelines for animal studies described in the Animal Care and Welfare Act (7 USC 2142 § 13) and were approved by the National Heart, Lung, and Blood Institute Animal Care and Use Committee (Animal Protocol NHLBI-H-0308). Mice were fed rodent chow pellets (LabDiet, Cat. No. 5021) and maintained on a 12-h light, 12-h dark cycle at 20–26 °C with humidity at 30-70%. Free access to food and water were provided and pups were weaned at ~P21. To harvest tissues and organs, mice were euthanized using isoflurane anesthesia followed by cervical dislocation. Reporting of results abides by the ARRIVE guidelines for reporting of animal experiments.

### Generation of transgenic mice

To investigate the in vivo function of *Tmem65* gene, we first attempted to generate a mouse line with total body *Tmem65* knockout by co-microinjecting Cas9 mRNA with two single guide RNAs (sgRNAs) targeting Exon 1 and Exon 4 (the italic and underlined nucleotides are PAM):

Tmem-Ex1: gccgtgttcagcgcctccat*ggg*

Tmem-Ex4: gcgatacctttcgtagggtt*tgg*

into zygotes collected from C57BL/6N mice, but all of the 19 offspring died between 2- and 4-week of age. Since hybrid mice show hybrid vigor and sometimes can help overcoming lethality, we next injected the same CRISPR reagents into zygotes collected from B6CBA/F1 hybrid mice (JAX #100011), but again all of the 26 offspring died between 2 and 4 weeks. Some of these offspring were genotyped by PCR either shortly before or shortly after their death, and all of them turned out to be homozygous mutants, indicating that these pair of sgRNAs are extremely potent at making deletions at their target sites. In attempting to obtain heterozygous mutant mice by reducing CRISPR cutting potency, we then microinjected Cas9 mRNA with each of these two sgRNAs alone. All of the 18 offspring injected with Tmem-Ex4 sgRNA died, and all but one of the 24 pups injected with Tmem-Ex1 sgRNA died before they are one month old. The single surviving mouse appeared normal and bred well. However, PCR and sequencing analyses of this founder and its offspring revealed that this line had a 3 bp deletion in Exon 1, which led to the in frame deletion of E58 from the TMEM65 protein. Therefore, it is a point mutation mouse line rather than a *Tmem65* knockout.

The TMEM65 conditional KO mouse line was generated by sequentially inserting *loxP* sites into Intron 3 and Intron 4, so that Exon4 can be excised by exposing to Cre recombinase, resulting to translation frame shift. Briefly, the Intron 3 *loxP* was inserted by co-microinjecting Cas9 mRNA with the Tmem-In3 sgRNA:

gtagttttgtgatagtcctc*agg*

and the Tmem-In3 donor oligos:

gaatgtaggcatttttaaaaattattattacaaatacagagattagtagttttgtgatagtataacttcgtatagcatacattatacgaagttatggatcccctcaggcacaagagagaaatagactggcatgtgtcaggttaaatgggggcatcccgtg

into zygotes collected from C57BL6/N mice. The Intron 4 *loxP* was inserted by co-microinjecting Cas9 mRNA with the Tmem-In4 sgRNA:

cattctagaagtacagctag*agg*

and the Tmem-In4 donor oligos:

tcacgttgcaacatcttctgccttcgttgtgttgtgtagtattatgaaaagttccctctagataacttcgtatagcatacattatacgaagttataagcttctgtacttctagaatgctttatggattcaactattttggatattagcagtaaatagactg into zygotes collected from mice that already contain the Intron 3 *loxP* site. Mice containing both *loxP* sites on the same allele were expanded and use for conditional knockout experiments.

To generate whole-body TMEM65 KO mice, *Tmem65*^*fl/fl*^ mice were crossed with *CMV-Cre* transgenic mice (available from the Jackson Laboratory, stock no. 006054). To generate neuronal-specific TMEM65 KO mice, *Tmem65*^*fl/fl*^ mice were crossed with *Nestin-Cre* transgenic mice (the Jackson Laboratory, stock no. 003771). To generate skeletal muscle-specific TMEM65 KO mice, *Tmem65*^*fl/fl*^ mice were crossed with *Myf6-Cre* mice (the Jackson Laboratory, stock no. 010528).

The TMEM65-V5 mouse line was generated by adding a V5 tag sequence in-frame to the c-terminus of the *Tmem65* gene, immediately before the stop codon. Briefly, Cas9 mRNA was co-microinjected with the Tmem-V5 sgRNA:

atcttcttataggtgttcta*agg*

and the Tmem-V5 donor oligos:

ggaatgttcccgttgattttctttggaggaagtgaagaggatgagaaactggaaacaacaaatggcaagcccatccccaaccccctgctgggcctggacagcacctaatcacgtttcaacacctataagaagatgtaaactaatgtacctcatcattaactatactgtccccacagttagc

into zygotes collected from the *Tmem65* conditional knockout mice. Mice containing both an intact V5 tag and correctly floxed Exon 4 were bred and used for experiments.

All primers and DNA oligos were generated by Integrated DNA Technologies (IDT). Cas9 mRNA was purchased from TriLink (Cat. No. L-7606). The sgRNAs were in vitro transcribed using ThermoFisher’s custom In Vitro transcription services.

### Plasmid

Plasmid encoding C-term Flag-tagged human NCLX (NM_024959) was purchased from OriGene.

### Mouse line genotyping

Genomic DNA was extracted from mouse tail snips and standard PCR was performed for genotyping.

The following primers were used for genotyping floxed mice:

5’ floxed site:

Forward: aaggactgcacagagatgtc

Reverse: acttgacaggagttatgctg

3’ floxed site:

Forward: cattctagactaccttaggtg

Reverse: gttagactctgtgaagctcac

The following pair of primers were used for genotyping *Tmem65*^*+V/+V*^ mice:

Forward: ccgttgattttctttggagg

Reverse: gaagaccaagccacaataag

Cre mice were genotyped according to the protocols from the Jackson Laboratory.

All primers and DNA oligos were generated by Integrated DNA Technologies (IDT).

### Western blot analysis

Mouse tissues were harvested and lysed mechanically with homogenizer in 1× RIPA lysis buffer (20–188, Sigma-Aldrich) containing 1× proteinase inhibitor cocktail (P8340, Sigma-Aldrich). Tissue lysates were incubated on ice for 10 minutes, and clarified by centrifugation at 10,000 × *g* for 10 min at 4 °C to pellet the tissue debris. Supernatants containing equal amounts of soluble proteins were mixed with 2× SDS protein sample buffer containing 100 mM DTT (Sigma-Aldrich), denatured by incubating on a heat block at 98 °C for 5 min, and were loaded on NuPAGE 10% Bis-Tris protein gels (Life Technologies). The gel was running at 150 V for about one hour to separate the proteins. Proteins were then transferred from Bis-Tris gel onto PVDF membrane via XCell II blot module (Invitrogen) for immunoblotting. The PVDF membrane blots were blocked with blocking buffer (Li-Cor P/N927-40000) for one hour, incubated in blocking buffer containing 0.1% Tween and primary antibodies for 3 h at room temperature or overnight at 4 °C, washed 3 times with 1× PBS containing 0.1% Tween (PBST), incubated with blocking buffer containing 0.1% Tween and IRDye secondary antibodies (Li-Cor), washed 3 times with PBST, and imaged with Azure c600 imaging system (Azure Biosystems). Chameleon duo pre-stained protein ladder (Li-Cor 928-60000) was used as a reference of molecular weights for detected proteins. The primary antibodies used were anti- TMEM65 (HPA025020, Sigma-Aldrich, 1:400; PA5-112762, Invitrogen, 1:1000), V5 (R960-25, Life Technologies, 1:2000), α-tubulin (P/N 926-42212, Li-Cor, 1:2000; 3873, Cell Signaling Technology, 1:1000), MCU (HPA016480, Sigma-Aldrich, 1:1000), LETM1 (16024-1-AP, Proteintech, 1:1000), NCLX (21430-1-AP, Proteintech, 1:1000), GHITM (16296-1-AP, Proteintech, 1:500), Complex IV subunit IV (A21348, Invitrogen, 1:1000), Oxphos rodent Western blot antibody cocktail (45-8099, Invitrogen, 1:1000), Heat shock protein 60 (PA5-79414, Invitrogen, 1:1000), VDAC (4611, Cell Signaling Technology, 1:1000). For Western blot of Oxphos rodent Western blot antibody cocktail, 50 °C for 5 min denaturing was performed. Uncropped and unprocessed scans of the blots are in the Source data of Gel blots.

### Co-Immunoprecipitation (Co-IP)

Co-IP experiment was performed with Dynabeads Co-IP kit (14321D, Life Technology). Brain tissues from *Tmem65*^*+V5/+V5*^ or mice *Tmem65*^*+V5/+*^ were dissected out and stored in −80 °C. 1 mg Dynabeads were weighed out and 5 μg anti-V5 antibody (R960-25, Life Technologies) were coupled to the beads at 37 °C overnight. Antibody-coupled beads were washed and stored at 4 °C before use. On the day of experiment, frozen brain tissue were homogenized in 1xIP buffer (25 mM Tris-HCl pH7.4, 150 mM NaCl, 1 mM EDTA, 1% NP-40, 1 mM PMSF, and 5% glycerol), incubated on ice for 15 min, and then centrifuged at 2600 × *g* for 5 min. Supernatant was collected, and concentration of protein were measured with Bradford method. 900 μl tissue lysate was incubated with pre-washed antibody-coupled beads on a roller at 4 °C for 30 min, then beads were collected and washed with wash buffer (Tris-HCl pH7.4, 150 mM NaCl, 0.02% Tween^TM^-20) for 3 times. Finally, 0.1 M glycine buffer (pH 3.0) was added to the washed beads to elute the proteins binding to the antibody-coupled beads. Western blot analysis was performed to evaluate the Co-IP experiment.

### Tissue harvesting and fixation for H&E staining

Under anesthesia, P20 or older mice were perfused with 1xPBS solution, followed by perfusion of 1xPBS containing 4% paraformaldehyde (PFA). Mouse Brain tissue was dissected out and fixed in 1xPBS containing 4% PFA at 4 °C overnight. The fixed brain tissue was washed with 1xPBS, and stored in 70% ethanol for paraffin embedding. Paraffin embedding, tissue sectioning, and H&E staining were performed by Histoserv Inc (Germantown, MD).

### Confocal microscopy

Mouse was anesthetized with 5% Isoflurane through gas anesthesia system, and was perfused through heart with 1xPBS and then 4% paraformaldehyde in 1xPBS. Mouse tissue was harvested and further fixed for 4 h. Fixed tissue was washed with 1x PBS, and transferred into 30% sucrose in 1xPBS solution overnight till tissue sank to the bottom of the solution. The tissue was trimmed and embedded in optimal cutting temperature (O.C.T.) compound (Sakura). For skeletal muscles, tissue were immediately embedded in O.C.T. after harvesting. O.C.T. embedded tissues were then quickly frozen in isopentane prechilled in dry ice. 10 µm sections were cut in cryostat at −20 °C. Frozen tissue sections were blocked with blocking buffer (1xPBS containing 5% goat serum and 1% bovine serum albumin) for one hour, and then incubated in blocking buffer containing 0.05% Tween and primary antibodies overnight. Sections were washed 3 times with PBS containing 0.05% Tween, and then incubated in Alexa Fluor secondary antibodies (Invitrogen) for one hour. Coverslips were mounted on the sections with coverslips with Prolong gold antifade mountant (Life Technologies) after 3 washes of 1xPBS containing 0.05% Tween. Confocal images were collected with a Zeiss LSM 780 confocal microscope system. Primary antibodies used were to ATP5A (ab14748, Abcam, 1:200), V5 (13202S, Cell Signal Technology, 1:100; or R960-25, Life technologies, 1:200), TMEM65 (HPA025020, Sigma-Aldrich, 1:50), Desmin (D1033, Sigma-Aldrich, 1:100), Connexin 43 (ab11370, Abcam, 1:400), Myosin heavy chain type I (BA-D5, DSHB, 1:60), Myosin heavy chain type IIa (SC-71, DSHB, 1:100), Myosin heavy chain type IIb (BF-F3, DSHB, 1:80), Laminin (L9393, Sigma-Aldrich, 1:200).

### Inverted grip suspension test

7 WT (*Tmem65*^*+/+*^, C57BL/6 background, 4 females and 3 males) and 6 KO littermates (*Tmem65*^*−/−*^, 3 females and 3 males) mice of P21 were subject to inverted grip strength tests. Mouse was gently placed on top of a wire grid to allow its paws to attach the grid with its torsi horizontal. The grip was gently inverted 180 degrees so the mouse was suspended above a padded surface. Latency to fall was record and averaged for three trials, separated by a rest period. The maximum trial length was set to 180 s. For TMEM65 and MCU double KO experiment, 9 *Tmem65*^*+/+*^*::Mcu*^*+/+*^ (C57BL/6 and CD1 background, 6 females and 3 males), 9 *Tmem65*^*+/+*^*::Mcu*^*−/−*^ (same background, 4 females and 5 males), 5 *Tmem65*^*−/−*^*::Mcu*^*+/+*^ (same background, 3 females and 2 males), 5 *Tmem65*^*−/−*^*::Mcu*^*+/−*^ (same background, 2 females and 3 males), and 8 *Tmem65*^*−/−*^*::Mcu*^*−/−*^ (same background, 4 females and 4 males) were tested.

### Home cage monitoring

Paired breeding mice with new born liters containing at least 1 WT (*Tmem65*^*+/+*^, C57BL/6 background) and 1 KO (*Tmem65*^*−/−*^) pups were moved into PhenoTyper home cages (Noldus, Wageningen, Netherlands) equipped with camera mounted in the cage lids for 24 h video monitoring to record episodes of epilepsy and any other behavior changes in the pups.

### Indirect calorimetry

The Oxymax-CLAMS setup (Columbus Instruments) was used to assess mouse metabolism. Mice were singly housed in the CLAMS chambers, received food and water ad libitum throughout the experiment, and were checked on at least twice a day throughout the experiment. Oxygen consumption, carbon dioxide production, food intake, and beam breaks (locomotion) were monitored during the experiment. The resulting data was analyzed using CalR. For muscle specific KO study, 11 *Tmem65*^*fl/fl*^ (C57BL/6 and 129 background, 7 females and 4 males) and 10 *Tmem65*^*fl/fl*^*::Myf6*^*Cre*^ littermates (same background, 8 females and 2 males) of 6-month-old mice were tested. For TMEM65/MCU double KO study, 8 *Tmem65*^*+/+*^*::Mcu*^*+/+*^ (C57BL/6 and CD1 background, 6 females and 2 males), 9 *Tmem65*^*+/+*^*::Mcu*^*−/−*^ (same background, 4 females and 5 males), and *Tmem65*^*−/−*^*::Mcu*^*−/−*^ (same background, 4 females and 3 males) 3.5-month-old mice were tested.

### Body composition

Whole body lean and fat mass was measured using the EchoMRI™ body composition analyzers for live mice before and after CLAMS. 11 *Tmem65*^*fl/fl*^ (C57BL/6 and 129 background, 7 females and 4 males) and 10 *Tmem65*^*fl/fl*^*::Myf6*^*Cre*^ littermates (same background, 8 females and 2 males) of 6-month-old mice were tested.

### Running wheel

The mice were housed individually, received food and water freely, and the running activity was measured using low-profile wireless running wheel (Med Associates Inc.) For muscle specific KO study, 11 *Tmem65*^*fl/fl*^ (C57BL/6 and 129 background, 7 females and 4 males) and 10 *Tmem65*^*fl/fl*^*::Myf6*^*Cre*^ littermates (8 females and 2 males) mice were tested. For TMEM65/MCU double KO study, 8 *Tmem65*^*+/+*^*::Mcu*^*+/+*^ (C57BL/6 and CD1 background, 6 females and 2 males), 9 *Tmem65*^*+/+*^*::Mcu*^*−/−*^ (same background, 6 females and 3 males), 7 and *Tmem65*^*−/−*^*::Mcu*^*−/−*^ (same background, 4 females and 3 males) mice were tested.

### Magnetic resonance imaging (MRI)

A total of thirteen 20, 21, and 23 days old littermates of 6 WT (*Tmem65*^*+/+*^, C57BL/6 background, 3 females and 3 males) and 7 KO (*Tmem65*^*−/−*^, 3 females and 4 males) were anesthetized with 5% Isoflurane through gas anesthesia system and perfused with 1xPBS, and then 1xPBS containing 4% PFA and Magnevist (1:500, Bayer HealthCare Pharmaceuticals) to enhance the brain with MR contrast. The mouse head was dissected out and the skin was removed to obtain the brain within the skull. The samples were then stored in 1xPBS containing 4% PFA in 4 °C refrigerator. The brain remained inside the cranium for scanning to preserve the brain anatomy. Before the scan, samples were washed and equilibrated in 1xPBS for at least 24 h.

MRI acquisition was performed ex vivo on a 14 T Bruker (Billerica, MA, USA) microimaging system with a 10 mm linear radio frequency coil. A T2-weighted image was acquired using a 3D multislice multiecho (MSME) pulse sequence with 100 µm isotropic resolution and a matrix size of 160 × 84 × 80, and with TE/TR = 30/3000, nex = 1. To examine brain volume, masks for the brain parenchyma were created that excluded skull and other non-brain material using the automatic segmentation snake tool in ITK-SNAP^59^. The volumes of these parenchymal brain masks were measured using ITK-SNAP and the values were plotted for each specimen.

### Echocardiography and electrocardiography

Mouse heart echocardiography and electrocardiography (ECG) were performed at the NHLBI Phenotyping Core. 16 control *Tmem65*^*+/+*^ (C57BL/6 background, 9 females and 7 males) and 10 *Tmem65*^*−/−*^ littermates (4 females and 6 males) were tested at P21. Mice were lightly anesthetized with isoflurane and placed supine over a heated platform with ECG leads and a rectal temperature probe. The Vevo2100 ultrasound system (VisualSonics, Toronto, Canada) with a 30 MHz ultrasound probe (VisualSonics, MS-400 transducer) was used to acquire heart images. Measurements were made from standard 2D and M mode images from the parasternal long axis and mid-papillary short axis views of the left ventricle.

### Mass spectrometry

Soleus muscles were harvested quickly from 2-month-old *Tmem65*^*fl/fl*^ (C57BL/6 and 129 background, *n* = 4, 2 females and 2 males) and *Tmem65*^*fl/fl*^*::Myf6*^*cre*^ (same background, *n* = 4, 2 females and 2 males) mice and 10-month-old *Tmem65*^*fl/fl*^ (*n* = 3, all females) and *Tmem65*^*fl/fl*^*::Myf6*^*cre*^ (*n* = 4, all females) mice, fast frozen in dry ice and stored in −80 °C freezer. Frozen soleus muscles were individually transferred to 130 µl of urea-based lysis buffer, 6 M Urea, 2 M Thiourea, 50 mM Triethylammonium bicarbonate (TEAB). Tissues were homogenized with ceramic beads using 3 cycles of 40 s at 5500 rpm and 4 °C (Precellys® Cryolys Evolution, Bertin Technologies). Tissue lysates were further clarified by microcentrifuge spin columns (QIAshredder, Qiagen) to obtain clear protein lysate centrifuged at 10,000 × *g* for 2 min at 4 °C. The extracted protein supernatants were transferred to 1.5 ml microtubes for further processing. Bradford assay (Thermo Fisher Scientific) was used to estimate the protein concentration of lysate, 100 µg of each lysate was reduced, alkylated, delipidated, digested with trypsin and individually labeled with Tandem Mass Tag (TMT) 16plex labeling reagent kit as per manufacturer’s instructions (Thermo Fisher Scientific).

High pH reversed-phase liquid chromatography was performed on an offline Agilent 1200 series HPLC. The quenched TMT labeled peptides were all combined, concentrated and desalted using HLB Oasis 1cc, Waters) column per manufacturer’s instructions. The dried desalted peptides were resuspended in 0.1 ml 10 mM triethyl ammonium bicarbonate with 2% (v/v) acetonitrile. Peptides were loaded onto an Xbridge C18 HPLC column (Waters; 2.1 mm inner diameter ×100 mm, 5 μm particle size), and profiled with a linear gradient of 5–35% buffer B (90% acetonitrile, 10 mM triethyl ammonium bicarbonate) over 60 min, at a flowrate of 0.25 ml/min. The chromatographic performance was monitored by sampling the eluate with a diode array detector (1200 series HPLC, Agilent) scanning between wavelengths of 200 and 400 nm. Fractions were collected at 1 min intervals followed by fraction concatenation. Twelve concatenated fractions were dried and resuspended in 0.01% formic acid, 2% acetonitrile. Approximately 500 ng of peptide mixture was loaded per liquid chromatography-mass spectrometry run.

All fractions were analyzed on an Ultimate 3000-nLC coupled to an Orbitrap Fusion Lumos Tribrid instrument (Thermo Fisher Scientific) equipped with a nanoelectrospray source. Peptides were separated on an EASY-Spray C18 column (75 μm × 50 cm inner diameter, 2 μm particle size and 100 Å pore size, Thermo Fisher Scientific). Peptide fractions were placed in an autosampler and separation was achieved by 120 min gradient from 4 to 40% buffer B (100% ACN and 0.1% formic acid) at a flow rate of 300 nL/min. An electrospray voltage of 1.9 kV was applied to the eluent via the EASY-Spray column electrode. The Lumos was operated in positive ion data-dependent mode, using Synchronous Precursor Selection (SPS-MS3). Full scan MS1 was performed in the Orbitrap with a precursor selection range of 380–1400 m/z at nominal resolution of 1.2 ×10^5^. The AGC target and maximum accumulation time settings were set to 4 ×10^5^ and 50 ms, respectively. MS2 was triggered by selecting the most intense precursor ions above an intensity threshold of 5 ×10^3^ for collision induced dissociation (CID)-MS2 fragmentation with an AGC target and maximum accumulation time settings of 2 ×10^4^ and 75 ms, respectively. Mass filtering was performed by the quadrupole with 0.7 m/z transmission window, followed by CID fragmentation in the linear ion trap with 35% normalized collision energy in rapid scan mode and parallelizable time option was selected. SPS was applied to co-select 10 fragment ions for HCD-MS3 analysis. SPS ions were all selected within the 350–1300 m/z range and were set to preclude selection of the precursor ion and TMTC ion series. The AGC target and maximum accumulation time were set to 1 ×10^5^ and 150 ms (respectively) and parallelizable time option was selected. Co-selected precursors for SPS-MS3 underwent HCD fragmentation with 55% normalized collision energy and were analyzed in the Orbitrap with nominal resolution of 5 ×10^4^. The number of SPS- MS3 spectra acquired between full scans was restricted to a duty cycle of 3 s.

Raw data files were processed using Proteome Discoverer (v2.4, Thermo Fisher Scientific), with Sequest HT (Thermo Fisher Scientific) search node. The data files were searched against UniProtKB/Swiss-Prot mus musculus (17,030 reviewed, release 2020_10) protein sequence database, with carbamidomethylation of cysteine, TMT-Pro (+304.207) modification of lysines and peptide N-terminus set as static modifications; oxidation of methionine as dynamic. For SPS-MS3, the precursor and fragment ion tolerances of 10 ppm and 0.5 Da were applied, respectively. Up to two-missed tryptic cleavages were permitted. Percolator algorithm (v.3.02.1, University of Washington) was used to calculate the false discovery rate (FDR) of peptide spectrum matches (PSM), set to a *q*-value < 0.05. TMT 16-plex quantification was also performed by Proteome Discoverer by calculating the sum of centroided ions within 20 ppm window around the expected m/z for each of the 15 TMT reporter ions. Spectra with at least 60% of SPS masses matching to the identified peptide are considered as quantifiable PSMs. Quantification was performed at the MS3 level where the median of all quantifiable PSMs for each protein group was used for protein ratios.

### Gene ontology analyses

UniProt accession identifiers for up- or down-regulated proteins (*p* < 0.05 with 2 + -fold KO/WT difference) were entered in to the GO Enrichment Analysis interface at geneontology.org and assessed for enriched biological processes with a false discovery rate (FDR) < 0.05 in *Mus musculus*. To identify processes generally enriched in muscle tissue, accession identifiers for all 4524 proteins were run separately to generate a background enrichment profile. Biological processes with an FDR that were lower in the whole muscle proteome than in the KO-specific analyses were thus removed.

### Oxygen consumption of frozen muscle tissue lysates

Oxygen consumption of frozen muscle tissue lysate were modified according to the protocol in Acin-Perez R, et al.^37^. Fresh hindlimb muscles were harvested from 3 *Tmem65*^*fl/fl*^ (C57BL/6 and 129 background, 2 females and 1 male) and 3 *Tmem65*^*fl/fl*^*::Myf6*^*Cre*^ littermates (2 females and 1 male) of 6-month-old, fast frozen in dry ice, and stored in -80 °C freezer. Frozen hindlimb muscles were thawed on ice and red and white muscles were dissected out. Thawed muscle tissues were minced and homogenized in MAS buffer (70 mM sucrose, 220 mM mannitol, 5 mM KH2PO4, 5 mM MgCl2, 1 mM EGTA, 2 mM HEPES pH 7.4), and collagenase (0.25 mg/ml final concentration, Sigma-Aldrich) was added and samples were incubated at 37 °C for 30 min. Samples were then homogenized using Dounce homogenizer with 20–30 strokes, and centrifuged at 1000 × *g* for 10 min at 4 °C. Supernatant was collected, and protein concentration was determined by Bradford method. For oxygen consumption with Oxygraph+ system (Hansatech Instruments, Norfolk, England), oxygen calibration was performed on each experiment day. After oxygen calibration, 1 ml muscle tissue lysate in MAS buffer with 10 µg/ml cytochrome c was added to the chamber at 37 °C. After 2–3 min, 10 µl 100 mM NADH (1 mM final) was added. Oxygen consumption rate was measured continuously throughout the procedure.

### Muscle fiber isolation and calcium imaging

The FDB muscle was dissected from the mouse foot and digested in collagenase (Sigma) at 37 °C for 2–3 h. After digestion, individual fibers were released by gentle trituration in MEM culture medium, and plated on Matrigel-coated dishes. After 1 h incubation with 4 µM Rhod-2-AM and 100 nM MitoTracker Green (Thermo Fisher Scientific) at 37 °C, the buffer was gently exchanged with MEM culture medium. Fibers were imaged 20 minutes after. Rhod-2 and MitoTracker Green fluorescence were simultaneously measured on a Zeiss LSM 780 confocal microscope.

### Isolation of mitochondria from tissues

For isolation of skeletal muscle mitochondria, hind limb muscles were harvested from 2-month-old mice, and transferred into ~10 ml isolation medium (150 mM sucrose, 75 mM KCl, 50 mM Tris-HCl, 1 mM KH2PO4, 5 mM MgCl2, 1 mM EGTA, pH 7.4) on ice. Muscles were minced into small pieces in isolation medium, and 0.5 mg Subtilisin A (1 mg/ml in isolation buffer, Sigma-Aldrich Cat# P3910) were added. Before homogenization, 5 ml of isolation medium containing with 0.2% BSA were added. The minced muscle tissues were then homogenized for 15 s at 40% of full power with Polytron (IKA Works, INC. Model: Ultra Turrax T25). Homogenate was centrifuged at 700 × *g* for 10 min at 4 °C. After first spin, supernatant was poured into a clean tube chilled on ice and then centrifugated at 10,000 × *g* for 10 min at 4 °C. The resulting pellet (mitochondria) was resuspended in Buffer B (225 mM Mannitol, 75 mM Sucrose, 5 mM MOPS, 2 mM Taurine, 1 mM EGTA, pH 7.25), 0.2% bovine serum albumin (BSA). The enrichment of mitochondria were wash and centrifuged at 11,000 × *g* for 3 min 4 °C. The final mitochondrial pellet was resuspended in 1 ml of Buffer B without albumin to measure Mitochondrial protein concentration by Bradford method.

For isolation of brain mitochondria, mouse brain were minced and then homogenized in ice-cold Buffer B containing 0.1% BSA. Homogenate was centrifuged at 700 × *g* for 10 min at 4 °C to pellet unlysed cells, and the supernatant was centrifugated at 10,000 × *g* for 10 min to pellet crude mitochondria. The crude mitochondria were resuspended in 3.5 mL of 15% Percoll solution in Buffer B, which was layered on top of 3.7 mL 24% and 1 mL 40% Percoll solutions in Buffer B. The solutions were then centrifugated at 30,000 × *g* for 40 min. Non-synaptosomal mitochondria were collected from a band between the 24% and 40% Percoll layers. Mitochondria were then resuspended in Buffer B and centrifuged at 10,000 × *g* for 10 min. The final mitochondrial pellet was resuspended in 1 ml of Buffer B without albumin to measure Mitochondrial protein concentration by Bradford method.

### Mitochondrial membrane potential measurements

Mitochondrial membrane potential was measured in vitro in isolated skeletal muscle fibers, in vivo in tibialis anterior muscle fibers, and in isolated mitochondria from hindlimb skeletal muscle.

In vitro mitochondrial membrane potential was measured in skeletal muscle fibers isolated from the FDB of 2-month-old. After isolation, fibers were incubated Tyrode’s buffer (10 mM HEPES, 137 mM sodium chloride, 4.5 mM potassium chloride, 0.5 mM potassium phosphate, 10 mM glucose, 1.8 mM calcium chloride, 1 mM pyruvate, and 1 mM 2,3-butanedione monoxime, pH 7.4) containing 5 mM tetramethylrhodamine methyl ester (TMRM) and 200 nM MitoTracker Green dye (MTG; Thermo Fisher Scientific, Waltham, MA, USA) at 37 °C for 30 min before imaging.

For in vivo mitochondrial membrane potential measurement, 2-month-old muscle-specific TMEM65 KO mice and their littermate controls were continuously anesthetized with 5% isoflurane by nose cone. Using medical adhesive tape, animals were secured to a custom-3D-printed acrylic water-heated perfusion platform. The skin and fascia were removed to expose the tibialis anterior muscle fibers. A water-heated reservoir surrounded the exposed muscle fibers and was continuously perfused with Tyrode’s buffer and 2.5 nM TMRM. NADH autofluorescence and TMRM were imaged. To obtain a fully reduced NADH pool, 5 mM sodium cyanide was added to perfused buffer.

Isolated skeletal muscle cells and tibialis anterior muscle fibers were imaged using an upright SP8 microscope (Leica) with a 25× (1.1 NA) water-immersion objective (Nikon, Tokyo, Japan). In vitro images were collected with confocal sequential line scanning and internal HyD detectors. MTG and TMRM were imaged with 488 nm and 552 nm excitation and 500–550 nm and 590–650 nm emission, respectively. In vivo images were taken using two-photon imaging with resonant scanning. NADH autofluorescence was excited using a Ti:Sapphire laser tuned to 750 nm. Emission for NADH was collected with a Leica HyD detector in the 414–538 nm range. Emission for TMRM was collected with a Leica HyD detector in the 538–605 nm range.

To measure mitochondrial membrane potential in isolated mitochondria from hindlimb skeletal muscle, hindlimb muscles were dissected from 2-month-old muscle-specific TMEM65 KO mice and their littermate controls. Dissected tissues were placed in 15 mL of Solution 1 (100 mM potassium chloride, 40 mM Tris-HCl, 10 mM Tris Base, 5 mM magnesium chloride, 1 mM EDTA, and 1 mM ATP, pH 7.4). Dissected skeletal muscle was minced on ice for 10 min before muscle homogenization with a Virtis blade for 10 s at 40% power. Subtilisin A (2.5 mg per 625 µL Solution 1) was added to the homogenized solution, and the digestion continued for 7 minutes while mixing continuously. An additional 15 mL of Solution 1 was added to the homogenized solution to dilute the protease. The homogenized solution was centrifuged at 2000 × *g* for 10 min at 4 °C to precipitate the cell debris. Supernatant was collected and filtered through a cheesecloth, and 250 µL of 2.67 mg/mL phenylmethylsulfonyl fluoride (PMSF) was added to terminate the protease digestion. Samples were centrifuged at 10,000 × *g* for 10 min at 4 °C to pull down the mitochondria. The mitochondrial pellet was resuspended in 10 mL Solution 2 with a Dounce homogenizer (100 mM potassium chloride, 40 mM Tris-HCl, 10 mM Tris-Base, 5 mM magnesium chloride, 1 mM EDTA, 1 mM ATP, and 1.5% BSA, pH7.4). The resuspended mitochondria were spun at 7000 × *g* for 10 min at 4 °C, and the mitochondria pellets were resuspended with a Dounce homogenizer in 5 mL of Solution 3 (100 mM potassium chloride, 40 mM Tris-HCl, 10 mM Tris Base, 1 mM magnesium chloride, 0.1 mM EDTA, 0.2 mM ATP, pH7.4). The last centrifugation spin was completed at 35,000 × *g* for 10 min at 4 °C. The mitochondrial pellets were collected and suspended in 250–350 µL mannitol-sucrose buffer using a Dounce homogenizer (220 mM mannitol, 70 mM sucrose, 10 mM Tris-HCl, 1 mM EGTA, pH 7.4).

Isolated mitochondrial membrane potential was measured with Oxygraph+ system (Hansatech Instruments, Norfolk, England). After oxygen calibration, mitochondrial membrane potential was measured in respiration buffer (100 mM potassium chloride, 50 mM MOPS, 10 mM monopotassium phosphate, 10 mM magnesium chloride, 1 mM EDTA, and 0,2% BSA, pH 7.0). A 0.1 mM Triphenyl phosphate (TPP) stock was titrated until a final concentration of 6.23 µM was reached. 40 µL of isolated mitochondria was added to the chamber. A final concentration of 50 µM ADP was added. Additions of 2 µM glutamate, 2 µM malate, and 2 µM pyruvate were added for fuel. Finally, a large bolus of ADP, final concentration of 500 µM, to stimulate respiration. A TPP electrode measured the voltage across the inner mitochondrial membrane by measuring changes in the concentration of TPP within the solution after chemical additions. The Nernst’s equation calculates the millivoltage of the mitochondrial membrane potential, which then can be quantified at each state of respiration.

### Measurement of Ca^2+^ uptake/retention capacity and Ca^2+^ efflux from isolated mitochondria

For Mitochondrial Ca^2+^ uptake/retention experiments, extramitochondrial Ca^2+^ concentration was monitored using the low-affinity fluorescent Ca^2+^ indicator Calcium Green-5N as previously described^46^. 100 μg mitochondria were suspended to 200 μl in a respiration buffer (120 mM KCl, 10 mM Tris, 5 mM MOPS, 5 mM K_2_HPO_4_, 10mM L-glutamic acid, 5mM L-Malic acid, pH = 7.4) in a 96 well, black/clear, tissue culture-treated plate (Falcon). Calcium Green-5N was added to a final concentration of 1 μM. Successive increments of 5 μM Ca^2+^ were added to the wells and Calcium Green-5N fluorescent signals were recorded with a CLARIOstar Plus plate reader (BMG Labtech). To measure Ca^2+^ efflux from isolated mitochondria, 100 μg mitochondria were suspended to 200 μl in a respiration buffer (120 mM KCl, 10 mM Tris, 5 mM MOPS, 5 mM K_2_HPO_4_, 10 mM L-glutamic acid, 5mM L-Malic acid, pH = 7.4) with Calcium Green-5N added to a final concentration of 1 μM. For a standard experiment, mitochondria were loaded with a bolus of Ca^2+^ (50 μM), followed by addition of the MCU inhibitor Ru360 (3 μM) to block mitochondrial Ca^2+^ uptake, thereby enabling observation of mitochondrial Ca^2+^ efflux in isolation. To probe Na^+^ dependent Ca^2+^ release from mitochondria, 20 mM of NaCl was added to mitochondria in the presence of Ru360. Calcium Green-5N signal was recorded with a CLARIOstar Plus plate reader (BMG Labtech).

### Statistics

Results are expressed as mean ± SD. Statistical analyses were performed using GraphPad Prism 9 software. *P* value was reported on each test. A *P* value of less than 0.05 was considered statistically significant.

### Inclusion and ethics statement

All collaborators of this study have fulfilled the criteria for authorship required by Nature Portfolio journals have been included as authors, as their participation was essential for the design and implementation of the study. We confirm that the author list of this manuscript does not include any large language models.

### Study approval

Animal studies were approved by the National Heart, Lung, and Blood Institute Animal Care and Use Committee.

### Reporting summary

Further information on research design is available in the Nature Portfolio Reporting Summary linked to this article.

## Supplementary information

Supplementary Information

Description of Additional Supplementary Files

Supplementary Movie 1

Supplementary Data 1

Reporting Summary

Transparent Peer Review file

## Source data

Source Data 1

## Data Availability

Source data are provided with this paper. The mass spectrometry proteomics data have been deposited to the ProteomeXchange Consortium (dataset identifier PXD035605) via MassIVE (UCSD, San Diego, CA, USA) a member of the consortium (dataset identifier MSV000089991). Source data are provided with this paper.

## Code availability

No custom code was used in this work.

## References

[1] Pagliarini, D. J. et al. A mitochondrial protein compendium elucidates complex I disease biology. *Cell* **134**, 112–123 (2008). 18614015 10.1016/j.cell.2008.06.016 PMC2778844

[2] Nishimura, N., Gotoh, T., Oike, Y. & Yano, M. TMEM65 is a mitochondrial inner-membrane protein. *PeerJ* **2**, e349 (2014). 24765583 10.7717/peerj.349 PMC3994636

[3] Foulds, C. E. et al. Research resource: expression profiling reveals unexpected targets and functions of the human steroid receptor RNA activator (SRA) gene. *Mol. Endocrinol.* **24**, 1090–1105 (2010). 20219889 10.1210/me.2009-0427 PMC2870939

[4] Sasarman, F. et al. LRPPRC and SLIRP interact in a ribonucleoprotein complex that regulates posttranscriptional gene expression in mitochondria. *Mol. Biol. Cell* **21**, 1315–1323 (2010). 20200222 10.1091/mbc.E10-01-0047 PMC2854090

[5] De Stefani, D., Raffaello, A., Teardo, E., Szabo, I. & Rizzuto, R. A forty-kilodalton protein of the inner membrane is the mitochondrial calcium uniporter. *Nature* **476**, 336–340 (2011). 21685888 10.1038/nature10230 PMC4141877

[6] Sharma, P. et al. Evolutionarily conserved intercalated disc protein Tmem65 regulates cardiac conduction and connexin 43 function. *Nat. Commun.* **6**, 8391 (2015). 26403541 10.1038/ncomms9391

[7] Kim, S. et al. Transmembrane glycine zippers: physiological and pathological roles in membrane proteins. *Proc. Natl. Acad. Sci. USA* **102**, 14278–14283 (2005). 16179394 10.1073/pnas.0501234102 PMC1242278

[8] Tegenfeldt, F. et al. OrthoDB and BUSCO update: annotation of orthologs with wider sampling of genomes. *Nucl. Acids Res.* **53**, E516–E522 (2025). 10.1093/nar/gkae987 PMC11701741 39535043

[9] Zhang, J. L. et al. TMEM65 functions as the mitochondrial Na+/Ca2+ exchanger. *Nat. Cell Biol.* **27**, 1301–1310 (2025). 10.1038/s41556-025-01721-x PMC12768467 40691517

[10] Nazli, A. et al. A mutation in the TMEM65 gene results in mitochondrial myopathy with severe neurological manifestations. *Eur. J. Hum. Genet.* **25**, 744–751 (2017). 28295037 10.1038/ejhg.2017.20 PMC5477357

[11] Vetralla, M. et al. TMEM65-dependent Ca2+ extrusion safeguards mitochondrial homeostasis. *Nat. Commun.* **17**, 923 (2025). 41408045 10.1038/s41467-025-67647-y PMC12830682

[12] Garbincius, J. F. et al. TMEM65 regulates and is required for NCLX-dependent mitochondrial calcium efflux. *Nat. Metab.* **7**, 714–729 (2025). 40200126 10.1038/s42255-025-01250-9 PMC12087536

[13] Glancy, B. et al. Mitochondrial reticulum for cellular energy distribution in muscle. *Nature* **523**, 617–620 (2015). 26223627 10.1038/nature14614 PMC6988728

[14] Schwenk, F., Baron, U. & Rajewsky, K. A cre-transgenic mouse strain for the ubiquitous deletion of loxP-flanked gene segments including deletion in germ cells. *Nucleic Acids Res.* **23**, 5080–5081 (1995). 8559668 10.1093/nar/23.24.5080 PMC307516

[15] Sato, S. Quantitative evaluation of ontogenetic change in heart rate and its autonomic regulation in newborn mice with the use of a noninvasive piezoelectric sensor. *Am. J. Physiol. Heart Circ. Physiol.* **294**, H1708–H1715 (2008). 18263713 10.1152/ajpheart.01122.2007

[16] Burte, F., Carelli, V., Chinnery, P. F. & Yu-Wai-Man, P. Disturbed mitochondrial dynamics and neurodegenerative disorders. *Nat. Rev. Neurol.* **11**, 11–24 (2015). 25486875 10.1038/nrneurol.2014.228

[17] Lake, N. J., Bird, M. J., Isohanni, P. & Paetau, A. Leigh syndrome: neuropathology and pathogenesis. *J. Neuropathol. Exp. Neurol.* **74**, 482–492 (2015). 25978847 10.1097/NEN.0000000000000195

[18] Liang, H., Hippenmeyer, S. & Ghashghaei, H. T. A Nestin-cre transgenic mouse is insufficient for recombination in early embryonic neural progenitors. *Biol. Open* **1**, 1200–1203 (2012). 23259054 10.1242/bio.20122287 PMC3522881

[19] Tronche, F. et al. Disruption of the glucocorticoid receptor gene in the nervous system results in reduced anxiety. *Nat. Genet* **23**, 99–103 (1999). 10471508 10.1038/12703

[20] Lucas, B. R. et al. Interventions to improve gross motor performance in children with neurodevelopmental disorders: a meta-analysis. *BMC Pediatrics* **16**, 1–16 (2016). 27899082 10.1186/s12887-016-0731-6 PMC5129231

[21] Emery, A. E. The muscular dystrophies. * Lancet* **359**, 687–695 (2002). 11879882 10.1016/S0140-6736(02)07815-7

[22] Sambasivan, R. et al. Embryonic founders of adult muscle stem cells are primed by the determination gene Mrf4. *Dev. Biol.* **381**, 241–255 (2013). 23623977 10.1016/j.ydbio.2013.04.018

[23] Southard, S. et al. A series of Cre-ER(T2) drivers for manipulation of the skeletal muscle lineage. *Genesis* **52**, 759–770 (2014). 24844572 10.1002/dvg.22792 PMC4441791

[24] Keller, C. et al. Alveolar rhabdomyosarcomas in conditional Pax3:Fkhr mice: cooperativity of Ink4a/ARF and Trp53 loss of function. *Genes Dev.* **18**, 2614–2626 (2004). 15489287 10.1101/gad.1244004 PMC525542

[25] Kallabis, S. et al. High-throughput proteomics fiber typing (ProFiT) for comprehensive characterization of single skeletal muscle fibers. *Skelet. Muscle* **10**, 7 (2020). 32293536 10.1186/s13395-020-00226-5 PMC7087369

[26] Clapham, J. C. et al. Mice overexpressing human uncoupling protein-3 in skeletal muscle are hyperphagic and lean. *Nature* **406**, 415–418 (2000). 10935638 10.1038/35019082

[27] Sowa, M. E., Bennett, E. J., Gygi, S. P. & Harper, J. W. Defining the human deubiquitinating enzyme interaction landscape. *Cell* **138**, 389–403 (2009). 19615732 10.1016/j.cell.2009.04.042 PMC2716422

[28] Nijman, S. M. et al. A genomic and functional inventory of deubiquitinating enzymes. *Cell* **123**, 773–786 (2005). 16325574 10.1016/j.cell.2005.11.007

[29] Li, W. et al. Genome-wide and functional annotation of human E3 ubiquitin ligases identifies MULAN, a mitochondrial E3 that regulates the organelle’s dynamics and signaling. *PLoS ONE* **3**, e1487 (2008). 18213395 10.1371/journal.pone.0001487 PMC2198940

[30] García-Prat, L. et al. Autophagy maintains stemness by preventing senescence. *Nature* **529**, 37–42 (2016). 26738589 10.1038/nature16187

[31] Brehme, M. et al. A chaperome subnetwork safeguards proteostasis in aging and neurodegenerative disease. *Cell Rep.* **9**, 1135–1150 (2014). 25437566 10.1016/j.celrep.2014.09.042 PMC4255334

[32] Rouillard, A. D. et al. The harmonizome: a collection of processed datasets gathered to serve and mine knowledge about genes and proteins. *Database* **2016**, baw100 (2016). 27374120 10.1093/database/baw100 PMC4930834

[33] Klaips, C. L., Jayaraj, G. G. & Hartl, F. U. Pathways of cellular proteostasis in aging and disease. *J. Cell Biol.* **217**, 51–63 (2018). 29127110 10.1083/jcb.201709072 PMC5748993

[34] Wang, X., Middleton, F. A., Tawil, R. & Chen, X. J. Cytosolic adaptation to mitochondria-induced proteostatic stress causes progressive muscle wasting. *iScience* **25**, 103715 (2022). 10.1016/j.isci.2021.103715 PMC8762400 35072007

[35] Guo, Q. et al. Mitochondrial proteostasis stress in muscle drives a long-range protective response to alleviate dietary obesity independently of ATF4. *Sci. Adv.* **8**, eabo0340 (2022). 35895846 10.1126/sciadv.abo0340 PMC9328690

[36] Ersoy, U. et al. Lifelong dietary protein restriction accelerates skeletal muscle loss and reduces muscle fibre size by impairing proteostasis and mitochondrial homeostasis. *Redox Biol.* **69**, 102980 (2024). 38064763 10.1016/j.redox.2023.102980 PMC10755587

[37] Acin-Perez, R. et al. A novel approach to measure mitochondrial respiration in frozen biological samples. * EMBO J.* **39**, e104073 (2020). 32432379 10.15252/embj.2019104073 PMC7327496

[38] Garbincius, J. F. & Elrod, J. W. Mitochondrial calcium exchange in physiology and disease. *Physiol. Rev.* **102**, 893–992 (2022). 34698550 10.1152/physrev.00041.2020 PMC8816638

[39] Brookes, P. S., Yoon, Y., Robotham, J. L., Anders, M. W. & Sheu, S. S. Calcium, ATP, and ROS: a mitochondrial love-hate triangle. *Am. J. Physiol. Cell Physiol.* **287**, C817–C833 (2004). 15355853 10.1152/ajpcell.00139.2004

[40] Austin, S. et al. TMBIM5 is the Ca2+/H+ antiporter of mammalian mitochondria. *EMBO Rep.* **23**, e54978 (2022). 36321428 10.15252/embr.202254978 PMC9724676

[41] Zhang, L. et al. TMBIM5 loss of function alters mitochondrial matrix ion homeostasis and causes a skeletal myopathy. *Life Sci. Alliance* **5**, e202201478 (2022). 10.26508/lsa.202201478 PMC9206080 35715207

[42] Bertero, E. & Maack, C. Calcium signaling and reactive oxygen species in mitochondria. *Circ. Res.* **122**, 1460–1478 (2018). 29748369 10.1161/CIRCRESAHA.118.310082

[43] Vercesi, A. E. et al. Mitochondrial calcium transport and the redox nature of the calcium-induced membrane permeability transition. *Free Radic. Biol. Med.* **129**, 1–24 (2018). 30172747 10.1016/j.freeradbiomed.2018.08.034

[44] Kolitsida, P. et al. Mfn2 induces NCLX-mediated calcium release from mitochondria. Preprint at https://www.biorxiv.org/content/10.1101/2024.08.05.606704v3 (2024).

[45] Zu, Y., Wan, L.-J., Cui, S.-Y., Gong, Y.-P. & Li, C.-L. The mitochondrial Na+/Ca2+ exchanger may reduce high glucose-induced oxidative stress and nucleotide-binding oligomerization domain receptor 3 inflammasome activation in endothelial cells. *J. Geriatr. Cardiol.* **12**, 270 (2015). 26089852 10.11909/j.issn.1671-5411.2015.03.003 PMC4460171

[46] Miao, X.-Y. et al. Astragalus polysaccharides reduce high-glucose-induced rat aortic endothelial cell senescence and inflammasome activation by modulating the mitochondrial Na+/Ca2+ exchanger. *Cell Biochem. Biophys.* **80**, 341–353 (2022). 35107747 10.1007/s12013-021-01058-w

[47] Pan, X. et al. The physiological role of mitochondrial calcium revealed by mice lacking the mitochondrial calcium uniporter. *Nat. Cell Biol.* **15**, 1464–1472 (2013). 24212091 10.1038/ncb2868 PMC3852190

[48] Palty, R. et al. NCLX is an essential component of mitochondrial Na+/Ca2+ exchange. *Proc. Natl. Acad. Sci. USA* **107**, 436–441 (2010). 20018762 10.1073/pnas.0908099107 PMC2806722

[49] Palty, R. et al. Lithium-calcium exchange is mediated by a distinct potassium-independent sodium-calcium exchanger. *J. Biol. Chem.* **279**, 25234–25240 (2004). 15060069 10.1074/jbc.M401229200

[50] Paillard, M. et al. Tissue-specific mitochondrial decoding of cytoplasmic Ca2+ signals is controlled by the stoichiometry of MICU1/2 and MCU. *Cell Rep.* **18**, 2291–2300 (2017). 28273446 10.1016/j.celrep.2017.02.032 PMC5760244

[51] Patron, M., Granatiero, V., Espino, J., Rizzuto, R. & De Stefani, D. MICU3 is a tissue-specific enhancer of mitochondrial calcium uptake. *Cell Death Differ.* **26**, 179–195 (2019). 29725115 10.1038/s41418-018-0113-8 PMC6124646

[52] Fieni, F., Bae Lee, S., Jan, Y. N. & Kirichok, Y. Activity of the mitochondrial calcium uniporter varies greatly between tissues. *Nat. Commun.* **3**, 1317 (2012). 23271651 10.1038/ncomms2325 PMC3818247

[53] Jadiya, P. et al. Neuronal loss of NCLX-dependent mitochondrial calcium efflux mediates age-associated cognitive decline. *Iscience* **26**, 106296 (2023). 10.1016/j.isci.2023.106296 PMC10014305 36936788

[54] Jadiya, P. et al. Impaired mitochondrial calcium efflux contributes to disease progression in models of Alzheimer’s disease. *Nat. Commun.* **10**, 3885 (2019). 31467276 10.1038/s41467-019-11813-6 PMC6715724

[55] Palty, R. & Sekler, I. The mitochondrial Na+/Ca2+ exchanger. *Cell calcium* **52**, 9–15 (2012). 22430014 10.1016/j.ceca.2012.02.010

[56] De Mario, A. et al. Identification and functional validation of FDA-approved positive and negative modulators of the mitochondrial calcium uniporter. *Cell Rep.* **35**, 109275 (2021). 34161774 10.1016/j.celrep.2021.109275 PMC8242467

[57] Singh, R. et al. Uncontrolled mitochondrial calcium uptake underlies the pathogenesis of neurodegeneration in MICU1-deficient mice and patients. *Sci. Adv.* **8**, eabj4716 (2022). 35302860 10.1126/sciadv.abj4716 PMC8932652

[58] Logan, C. V. et al. Loss-of-function mutations in MICU1 cause a brain and muscle disorder linked to primary alterations in mitochondrial calcium signaling. *Nat. Genet.* **46**, 188–193 (2014). 24336167 10.1038/ng.2851

[59] Yushkevich, P. A. et al. User-guided 3D active contour segmentation of anatomical structures: significantly improved efficiency and reliability. *Neuroimage* **31**, 1116–1128 (2006). 16545965 10.1016/j.neuroimage.2006.01.015

