# Supplementary material for: Loss of TMEM65 in mice causes mitochondrial disease mediated by mitochondrial Ca2+
Source: Nat Commun. 2026 Apr 14;17:5203. doi: 10.1038/s41467-026-71761-w (PMC13254257; doi:10.1038/s41467-026-71761-w)
Supplement: Supplementary file 1 — Supplementary Information [file 41467_2026_71761_MOESM1_ESM.pdf]

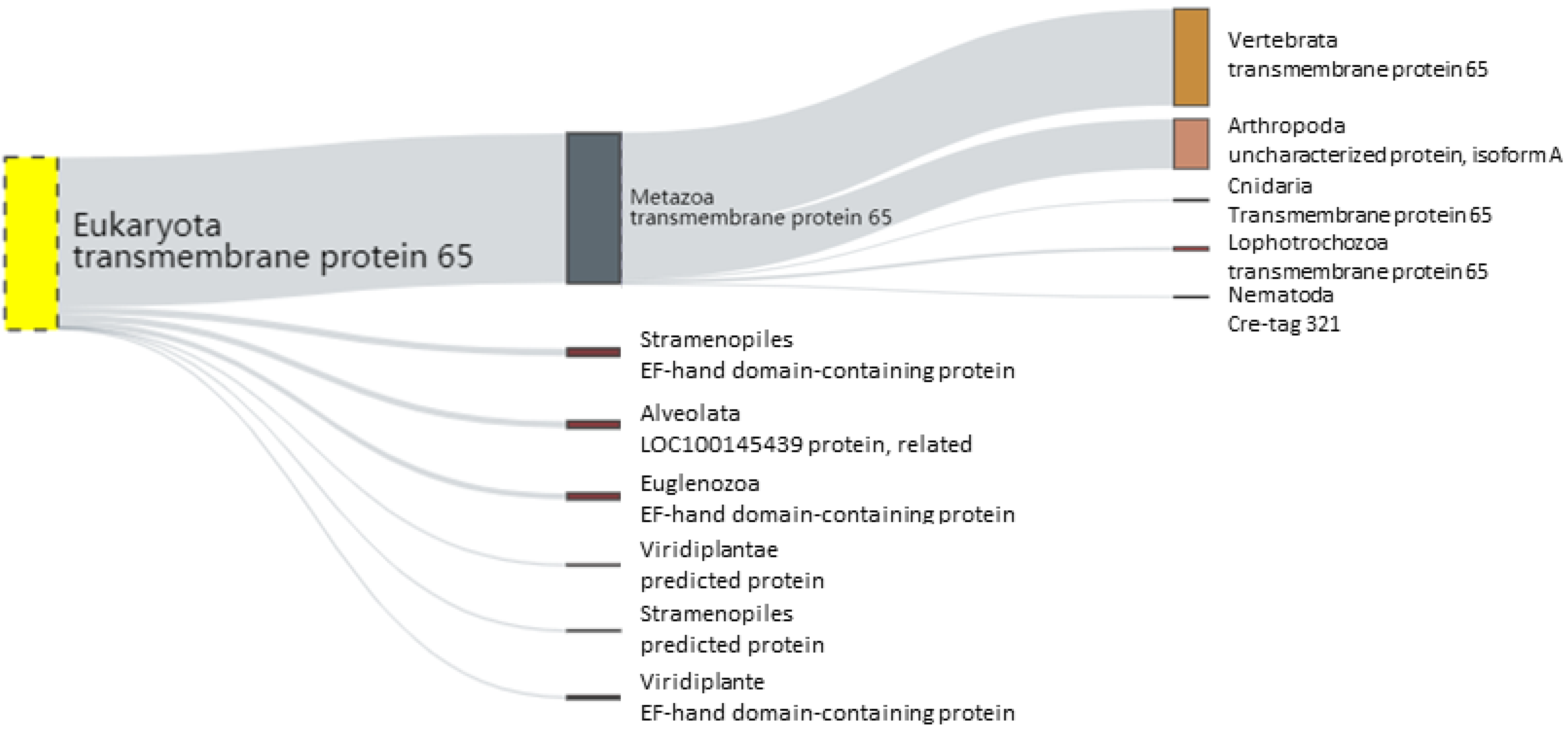

Supplementary Figure 1. Orthologs of *Tmem65*. Sankey diagram showing the orthologs of *Tmem65* in Eukaryote. EF-hand domain is found in many non-Metazoan *Tmem65* orthologs, indicating calcium involvement in TMEM65 function. Created from OrthoDB version 11.

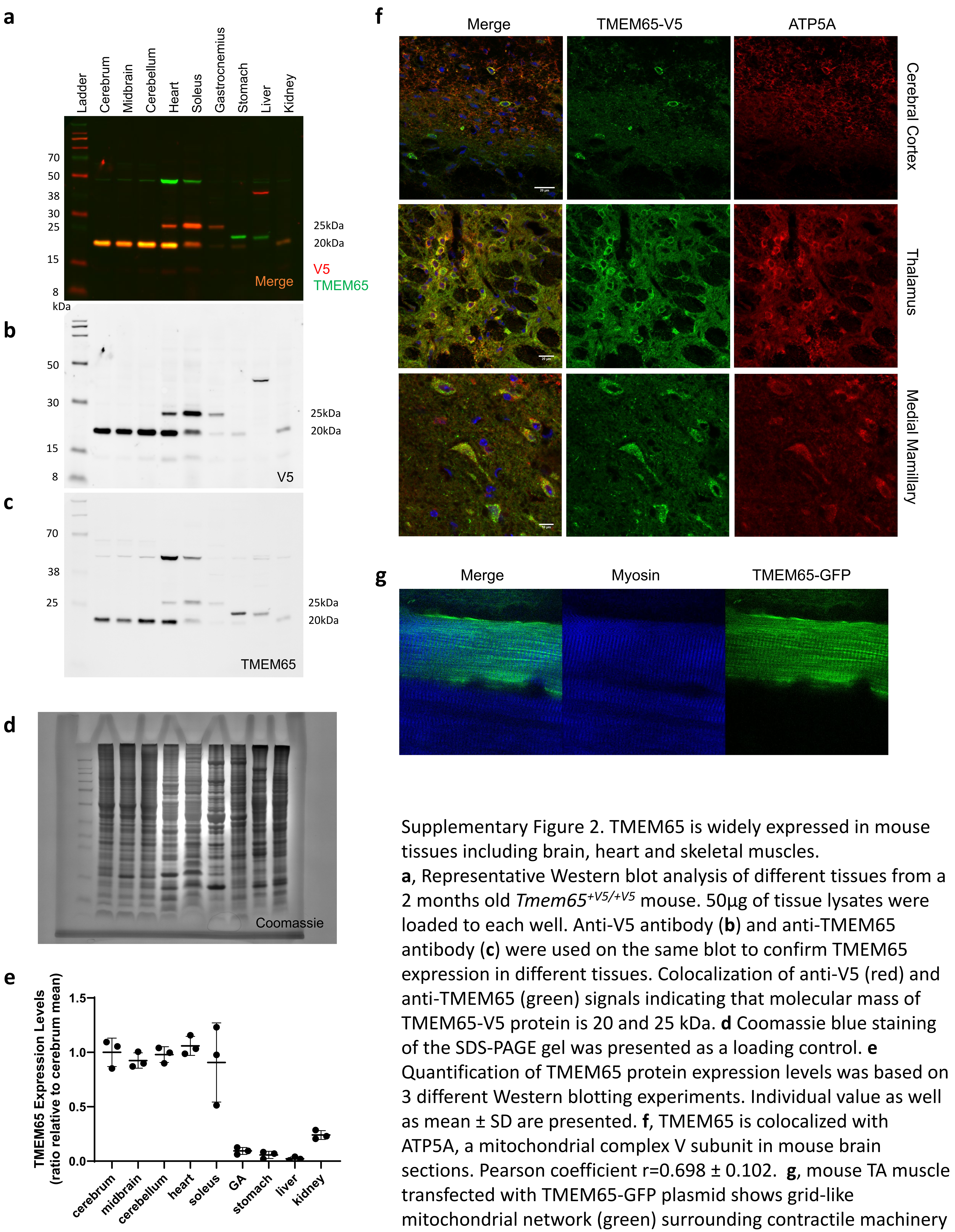

Supplementary Figure 2. TMEM65 is widely expressed in mouse tissues including brain, heart and skeletal muscles.
a, Representative Western blot analysis of different tissues from a 2 months old *Tmem65*$^{+V5/+V5}$ mouse. 50µg of tissue lysates were loaded to each well. Anti-V5 antibody (**b**) and anti-TMEM65 antibody (**c**) were used on the same blot to confirm TMEM65 expression in different tissues. Colocalization of anti-V5 (red) and anti-TMEM65 (green) signals indicating that molecular mass of TMEM65-V5 protein is 20 and 25 kDa. **d** Coomassie blue staining of the SDS-PAGE gel was presented as a loading control. **e** Quantification of TMEM65 protein expression levels was based on 3 different Western blotting experiments. Individual value as well as mean ± SD are presented. **f**, TMEM65 is colocalized with ATP5A, a mitochondrial complex V subunit in mouse brain sections. Pearson coefficient r=0.698 ± 0.102. **g**, mouse TA muscle transfected with TMEM65-GFP plasmid shows grid-like mitochondrial network (green) surrounding contractile machinery (blue) of the muscle fiber in vivo.

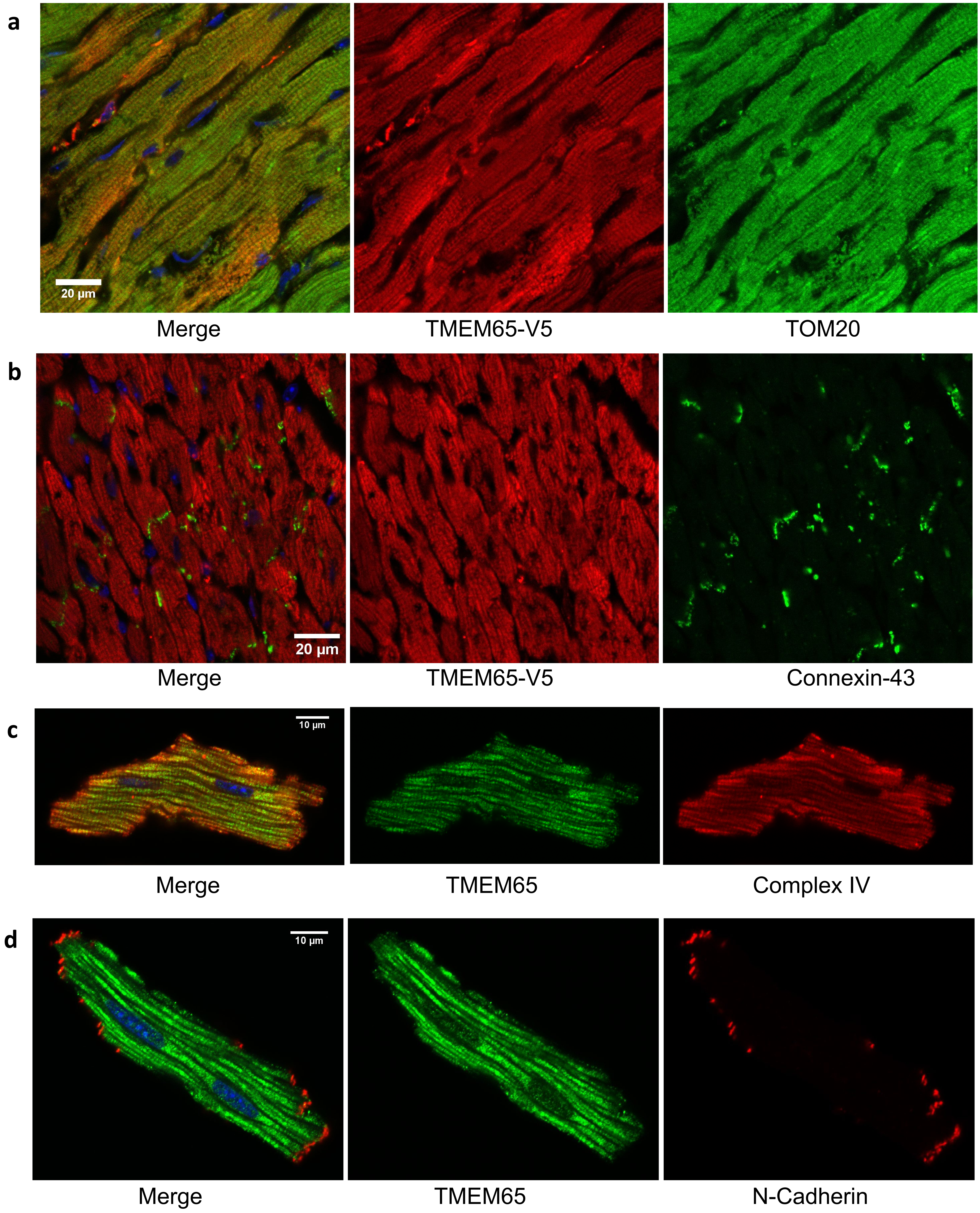

Supplementary Figure 3. TMEM65 is a mitochondrial protein expressed in mouse heart.
a, Heart section from a *Tmem65*$^{+V5/+V5}$ mouse was stained with anti-V5 and anti-TOM20 antibodies to show TMEM65 is a mitochondrial protein in the heart. Pearson coefficient r=0.578. **b,** Heart section from a *Tmem65*$^{+V5/+V5}$ mouse was stained with anti-V5 and anti-Connexin-43 antibodies to show TMEM65 is not colocalized with Connexin-43, an intercalated disc protein in mouse heart. Pearson coefficient r=0.068. **c,** Isolated mouse cardiac myocyte was stained with anti-TMEM65 and anti-Complex IV subunit IV antibodies to show TMEM65 is a mitochondrial protein in cardiac myocyte. Pearson coefficient r= 0.877. **d,** Isolated mouse cardiac myocyte was stained with anti-TMEM65 and anti-N-Cadherin to show that TMEM65 is not colocalized with N-Cadherin, an intercalated disc protein in cardiac myocyte. Pearson coefficient r=0.139.

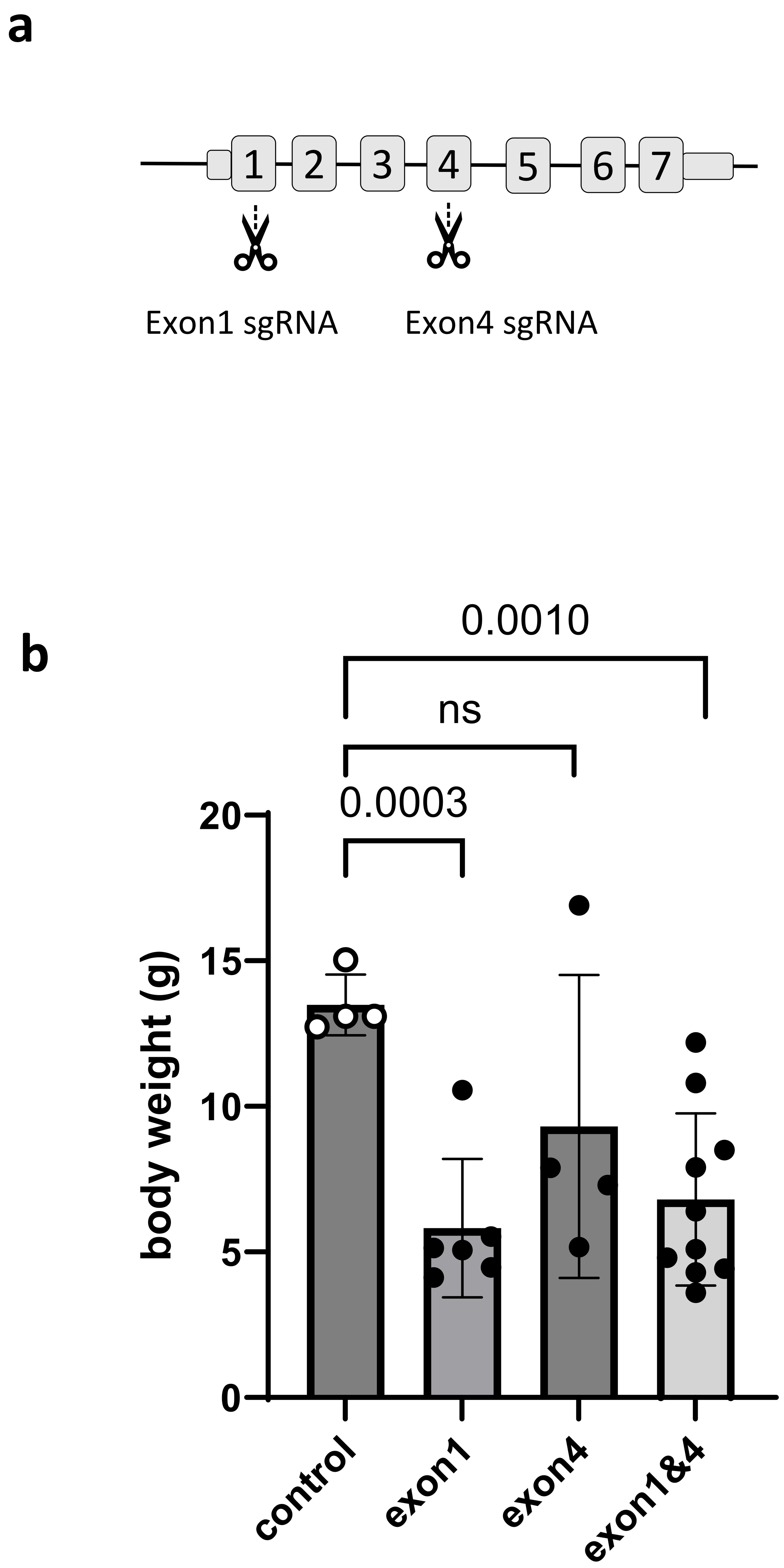

Supplementary Figure 4. **a,** Schematic of TMEM65 KO mouse with sgRNAs targeting exon1 and/or exon4 of *Tmem65* using CRISPR methodology. b, Body weights were smaller in *Tmem65* KO mice at P21 (one-way ANOVA P=0.0047). n = 4 for control, n = 6 for exon1 KO, n = 4 for exon4 KO, and n = 10 for exon1&4 KO. Two-tailed t test was used. Individual value as well as mean ± SD are presented

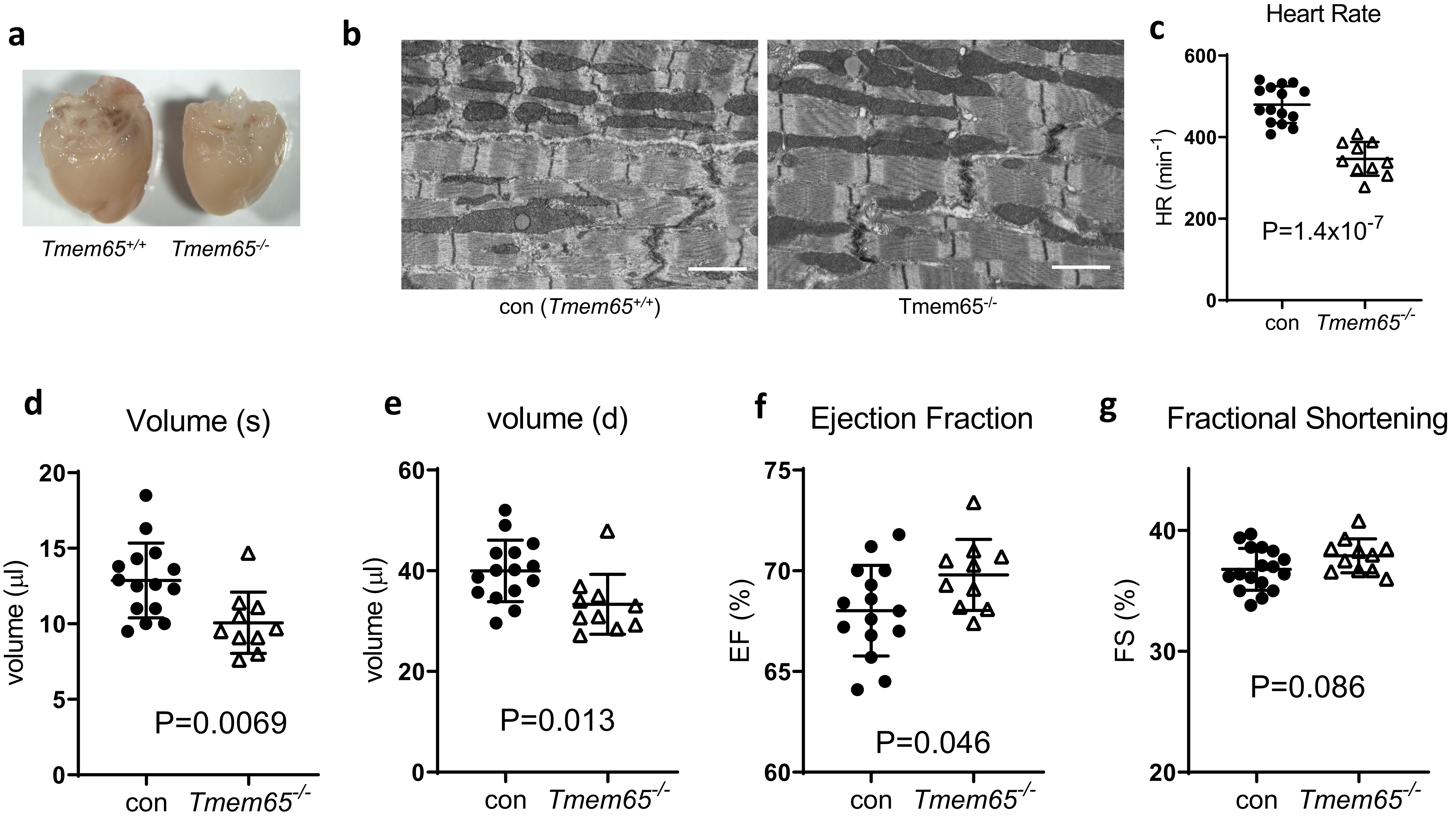

Supplementary Figure 5. Ablation of TMEM65 does not change heart structure and function.
a, Hearts from P21 littermates of control *Tmem65*$^{+/+}$ and *Tmem65*$^{-/-}$ pups. **b,** EM images of P20 hearts from *Tmem65*$^{+/+}$ control and *Tmem65*$^{-/-}$ littermates. Muscle contractile apparatus, mitochondria and intercalated discs are all normal in *Tmem65*$^{-/-}$ heart. Bar, 1 µm. **c,** *Tmem65*$^{-/-}$ mice have a lower heart rate compared to littermate control of *Tmem65*$^{+/+}$ at P21 **d-g,** Echocardiology results from P21 control *Tmem65*$^{+/+}$ and *Tmem65*$^{-/-}$ littermates. Systolic volumes (**d**) and diastolic volumes (**e**) were significantly lower, while ejection fraction (**f**) was higher in P21 *Tmem65*$^{-/-}$ hearts. **g,** Percentage of fractional shortening was similar between *Tmem65*$^{+/+}$ control and *Tmem65*$^{-/-}$ littermates. *Tmem65*$^{+/+}$ n =15; *Tmem65*$^{-/-}$ n = 10. Two-tailed t tests were used. Individual values as well as mean ± SD are presented.

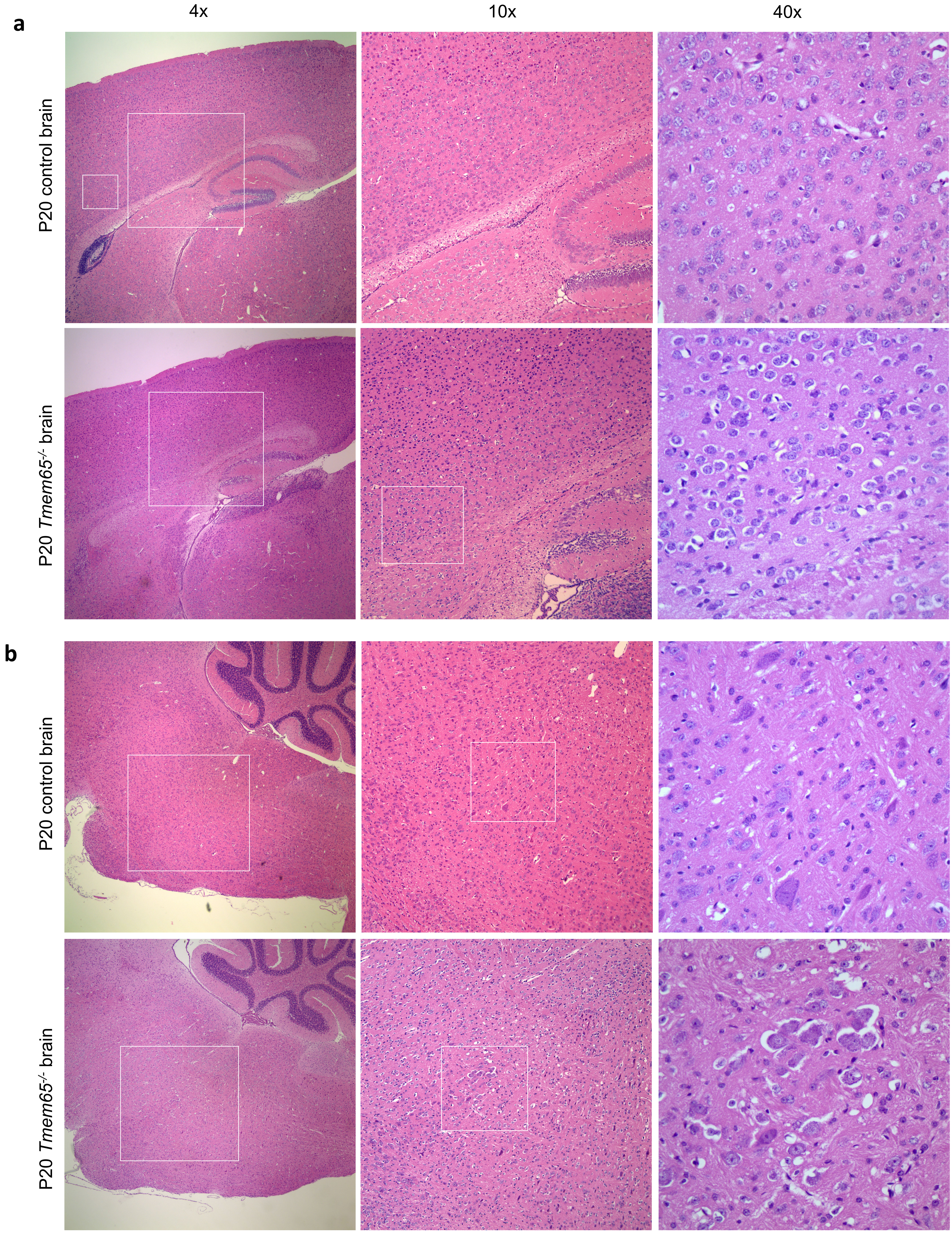

Supplementary Figure 6. Brain lesion in a P20 *Tmem65*$^{-/-}$ mouse.
H&E staining of brain sagittal sections show diffusive neuronal vacuolar degeneration in the cingulate cortex region (**a**), and brain stem area (**b**). Images were taken at 4x, 10x, and 40x.

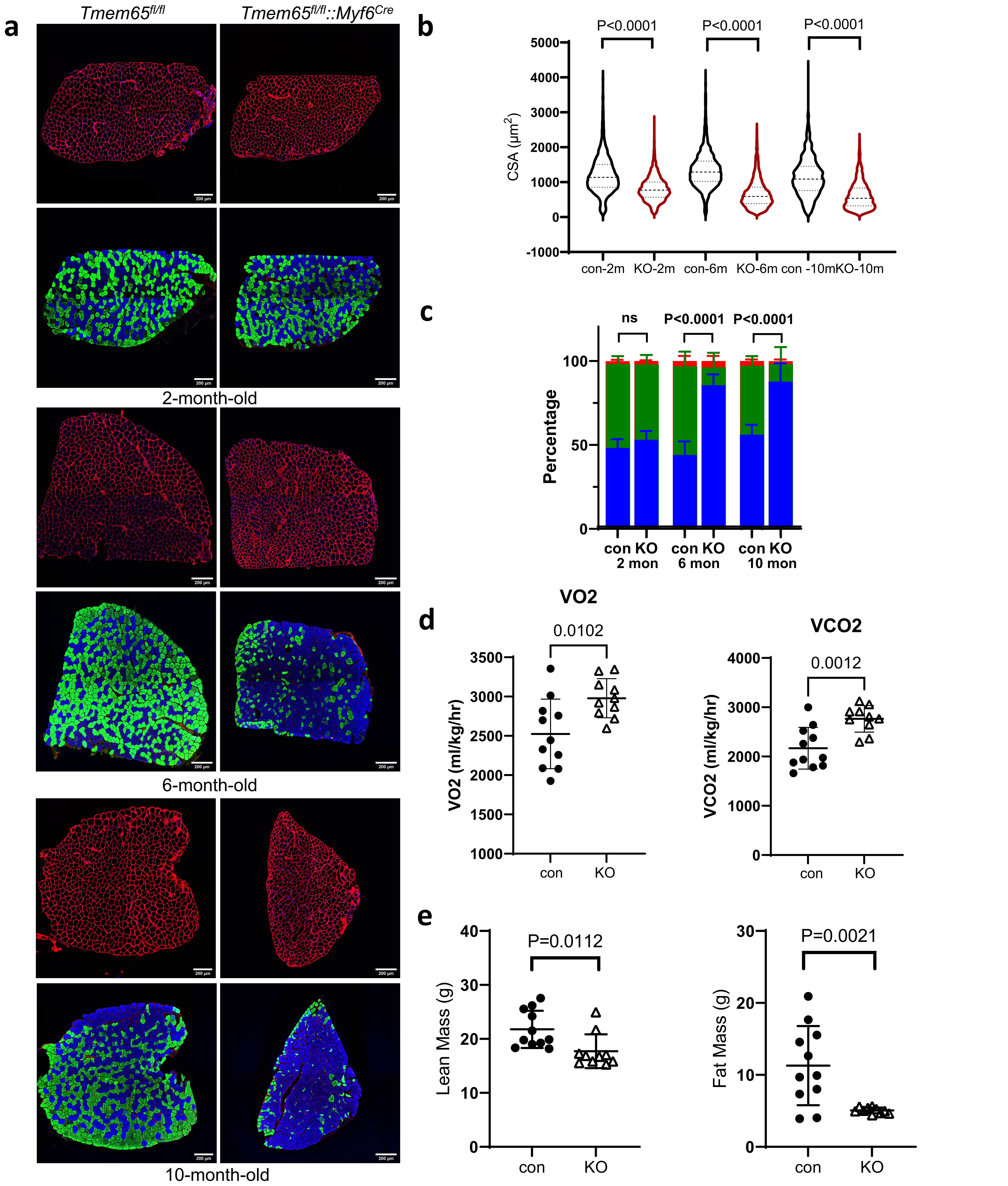

Supplementary Figure 7. Loss of TMEM65 in skeletal muscles causes muscular atrophy and muscle type switch.
a, Immunofluorescence of soleus muscles from control *Tmem65$^{fl/fl}$* and *Tmem65$^{fl/fl}$ ::Myf6^{Cre}* littermates at 2-month-old, 6-month-old, and 10-month-old. Anti-Laminin antibody staining (red, upper rows) delineated individual muscle fibers for cross-section area quantification and evaluation. Myosin isoforms were identified with specific antibodies to muscle type I (blue), IIa (green) and IIb (red), in lower rows. **b,** Quantification of soleus muscle fiber cross section area from control *Tmem65$^{fl/fl}$* and *Tmem65$^{fl/fl}$ ::Myf6^{Cre}* littermates of different ages. **c,** Quantification of myosin isoform composition in control *Tmem65$^{fl/fl}$* and *Tmem65$^{fl/fl}$ ::Myf6^{Cre}* soleus muscle cross sections at different age.(n = 3 replicates per group). **d,** VO2 and VCO2 results from 6-month-old control *Tmem65$^{fl/fl}$* and *Tmem65$^{fl/fl}$::Myf6^{Cre}* littermates collected in metabolic cages (CLAMS) for simultaneous measurement. *Tmem65$^{fl/fl}$ n* = 11; *Tmem65$^{fl/fl}$::Myf6^{Cre}* n = 10. **e,** EchoMRI body composition analysis of *Tmem65$^{fl/fl}$::Myf6^{Cre}* mouse at 6-month-old. *Tmem65$^{fl/fl}$* n = 11; *Tmem65$^{fl/fl}$::Myf6^{Cre}* n = 10. Two-tailed t tests were used. Individual values as well as mean ± SD are presented.

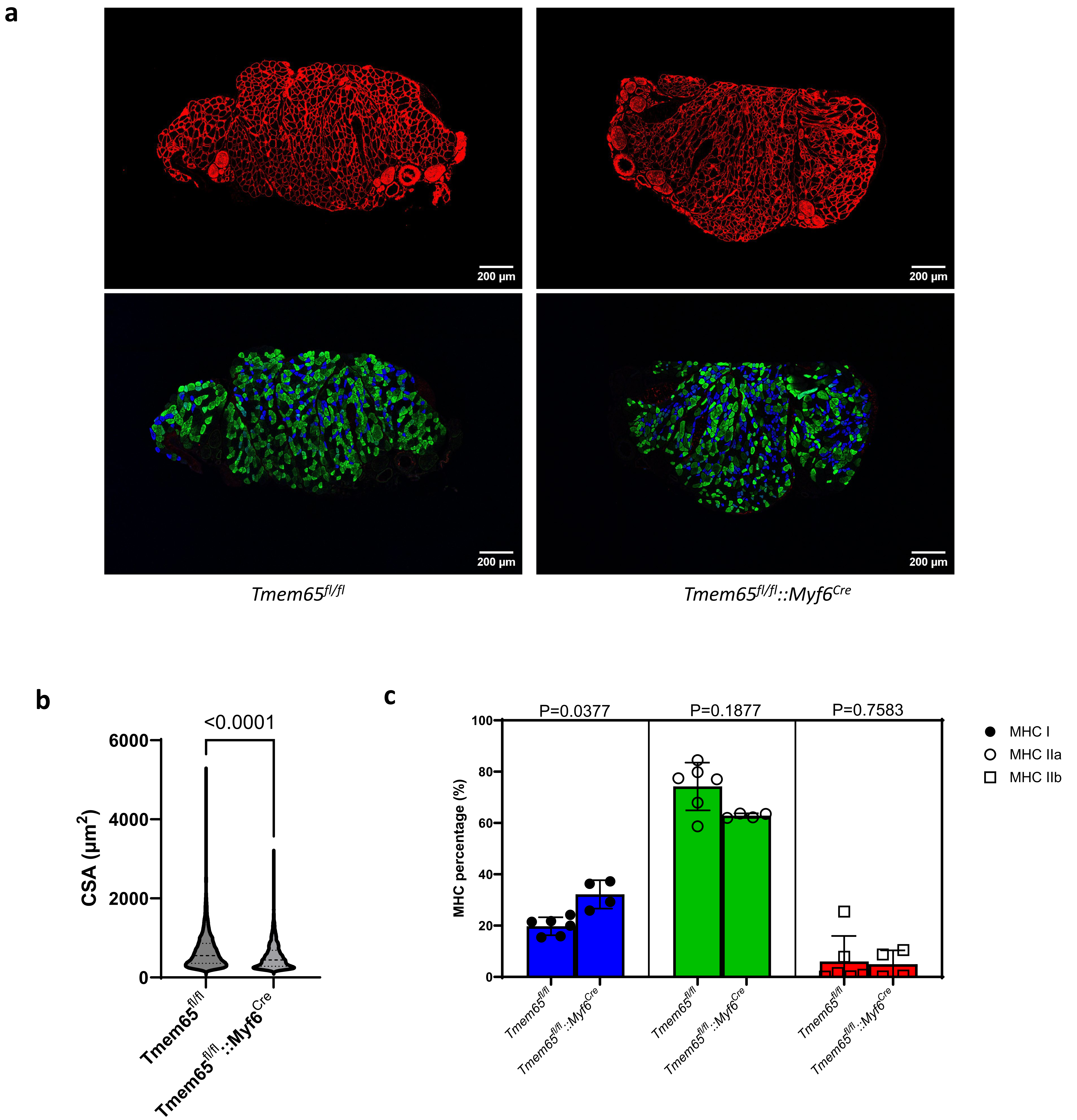

Supplementary Figure 8. Loss of TMEM65 in skeletal muscles causes muscular atrophy and muscle type switch in FDB muscle.
a, Immunofluorescence of FDB muscles from control *Tmem65*$^{fl/fl}$ and *Tmem65*$^{fl/fl}$ *::Myf6*^{Cre} littermates at 6-month-old. Anti-Laminin antibody staining (red, upper rows) delineated individual muscle fibers for cross-section area quantification and evaluation. Myosin isoforms were identified with specific antibodies to muscle type I (blue), IIa (green) and IIb (red). **b,** Quantification of FDB muscle fiber CSA from 6-month-old control *Tmem65*$^{fl/fl}$ and *Tmem65*$^{fl/fl}$ *::Myf6*^{Cre} littermates. **c.** Quantification of myosin isoform composition in 6-month-old control *Tmem65*$^{fl/fl}$ and *Tmem65*$^{fl/fl}$ *::Myf6*^{Cre} FDB muscle cross sections. n = 6 for *Tmem65*$^{fl/fl}$ and n = 4 for*Tmem65*$^{fl/fl}$ *::Myf6*^{Cre}. Two-tailed t test was used. Individual value as well as mean ± SD are presented.

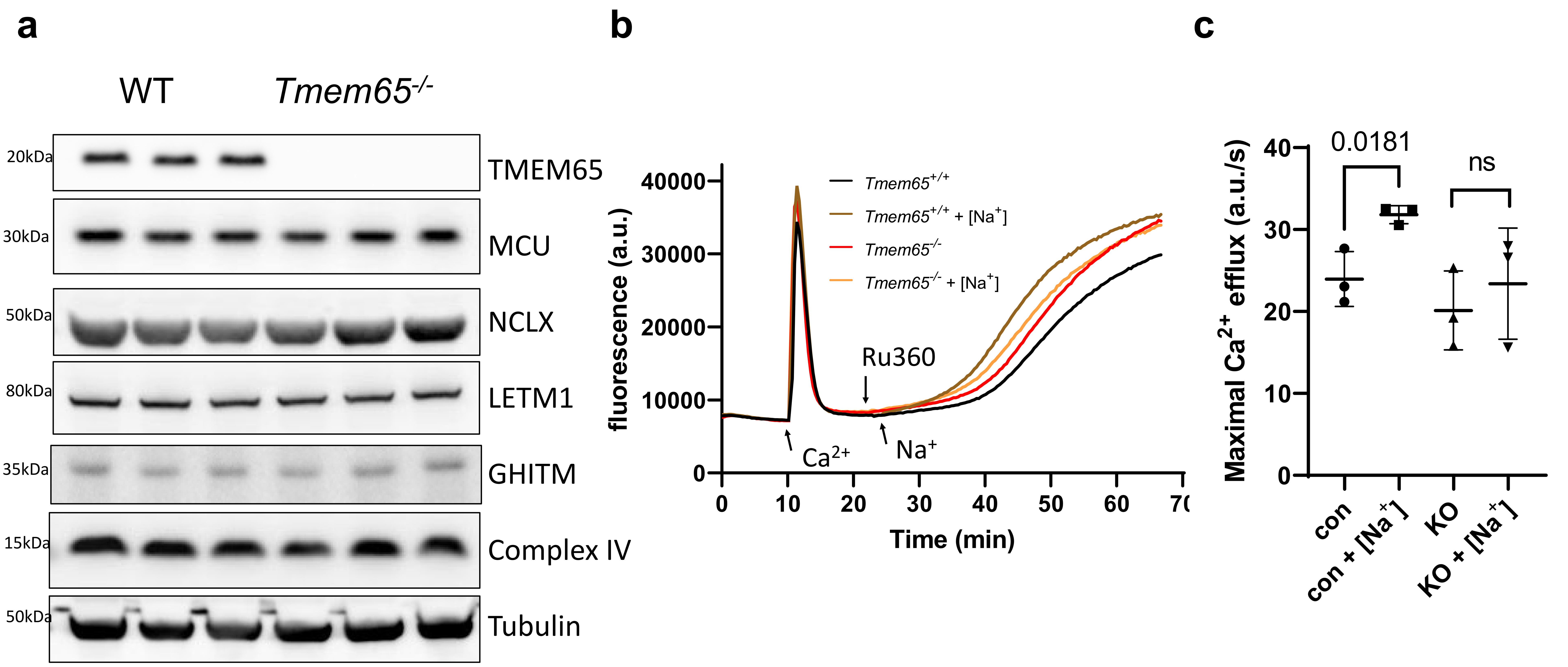

Supplementary Figure 9. TMEM65 regulates brain mitochondrial calcium release in Na^+ dependent manner

a, Western blot analysis of TMEM65, MCU, NCLX, LETM1, and GHITM protein expression in P19 brain tissues (n = 3 replicates per group). **b,** Representative traces of extramitochndrial Ca^{2+} ($[Ca^{2+}]_{ext}$) in a suspension of isolated brain mitochondria (100 µg/well). First, a bolus of Ca^{2+} (50 µM) was added, followed by addition of MCU inhibitor, Ru360 (3 µM) to block mitochondrial Ca^{2+} uptake; then 20 mM NaCl was added to induce Ca^{2+} efflux from mitochondria. **c,** Summary of the maximal rates of mitochondrial Ca^{2+} efflux induced by Ru360 alone, or Ru360 and Na^+ in *Tmem65*$^{fl/fl}$ control or *Tmem65*$^{fl/fl}$*::CMV*^{Cre} mitochondria isolated from brain. n = 3 replicates per group. Two tailed t test was used. Individual value as well as mean ± SD are presented.

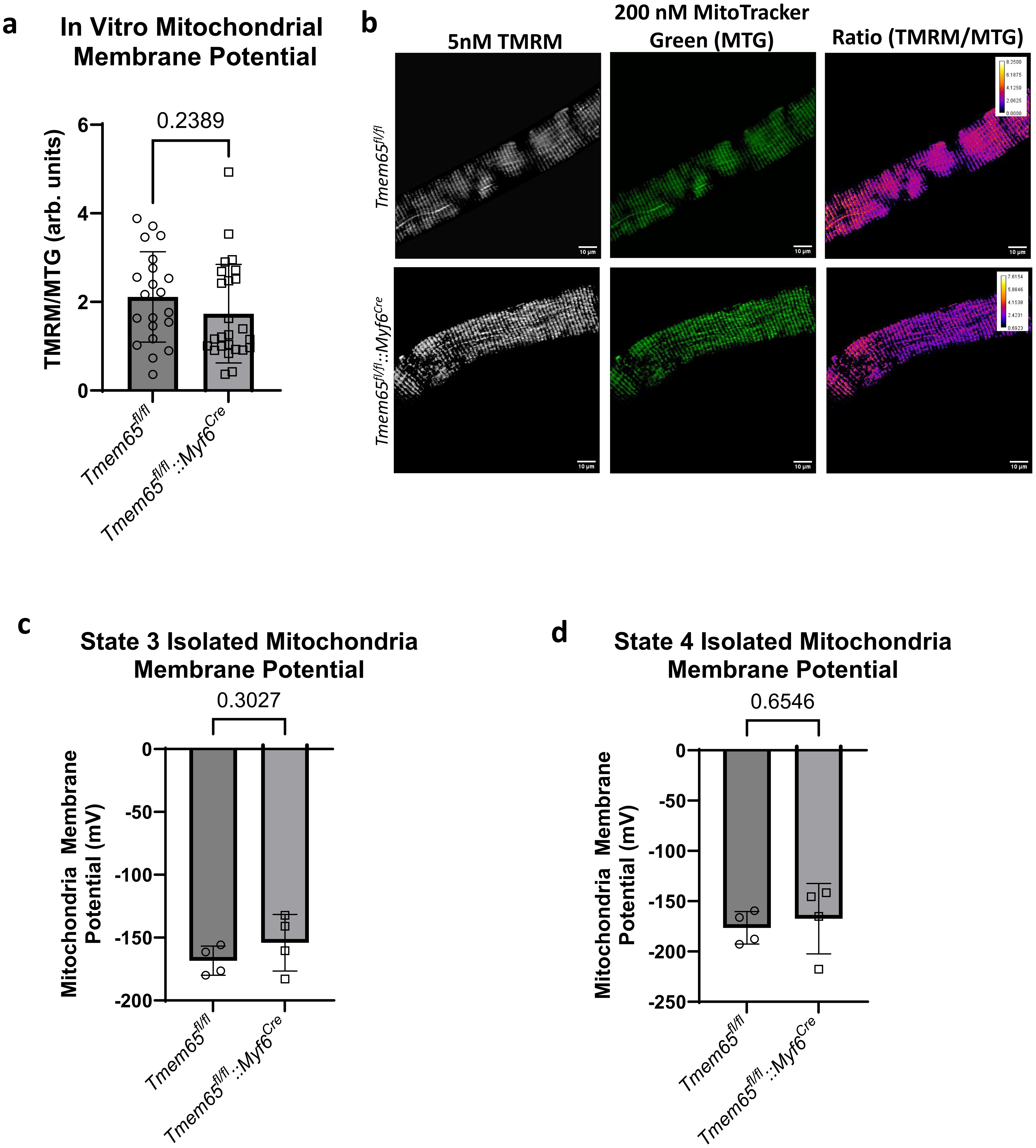

Supplementary Figure 10. Mitochondrial membrane potential was not affected by loss of TMEM65.
a, Mitochondrial membrane potential was measured in isolated FDB fibers from 2-month-old mice with TMRM. n = 21 from 8 *Tmem65*$^{fl/fl}$ mice, and n = 25 from 9 *Tmem65*$^{fl/fl}$*::Myf6*^{Cre} mice. Two-tailed t test was used. Individual value as well as mean ± SD are presented. **b,** Representative images of isolated FDB fiber for the measurement. **c,** Mitochondrial membrane potential was measured with isolated mitochondria from hindlimb muscles of 2-month-old mice at state 3. n = 4 replicates per group. Tow-tailed t test was used. Individual value as well as mean ± SD are presented. **d,** Mitochondrial membrane potential was measured with isolated mitochondria from hindlimb muscles of 2-month-old mice at state 4. n = 4 replicates per group. Two-tailed t test was used. Individual value as well as mean ± SD are presented.

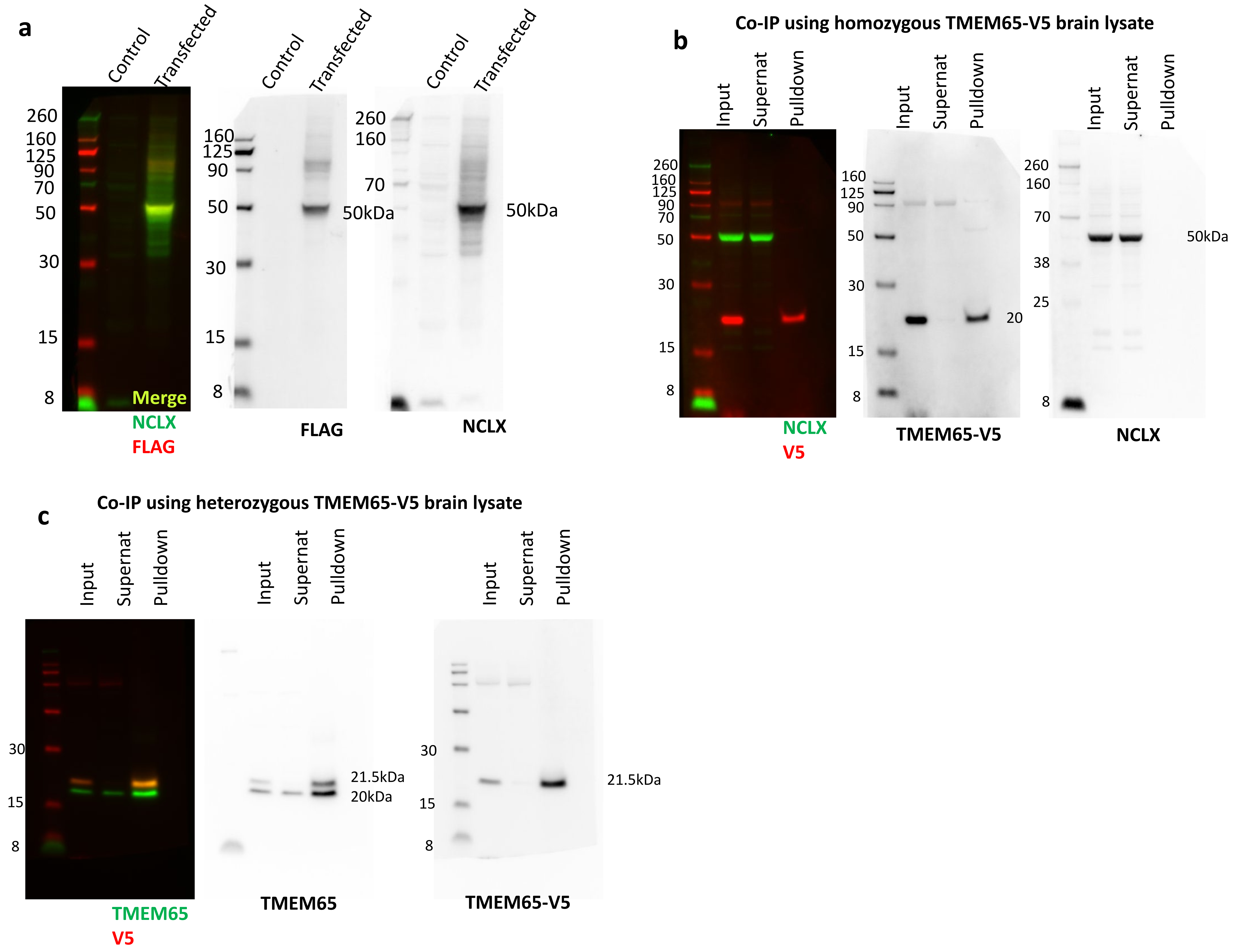

Supplementary Figure 11. TMEM65 does not interact with NCLX directly. **a,** Western blot analysis of HEK293T cells transfected with plasmid expressing NCLX-FLAG. Both anti-FLAG antibody and anti-NCLX antibody can detect a band of 50kDa in the same blot. Left lane: protein ladder; mid lane: control HEK293T cells without transfection; right lane: HEK293T cells transfected with NCLX-FLAG plasmid. **b,** Co-IP experiment using homozygous *Tmem65*$^{+V/+V}$ brain tissue lysate. Pulldown beads were conjugated with anti-V5 antibody. Anti-V5 beads can pull down TMEM65-V5 protein but can't pull down NCLX, indicating no direct interaction between TMEM65-V5 and NCLX in brain tissue. **c,** Co-IP experiment using heterozygous *Tmem65*$^{+V/+}$ brain tissue lysate. Pulldown beads were conjugated with anti-V5 antibody. Anti-V5 beads can pull down both TMEM65-V5 protein and TMEM65 protein, indicating direct interaction between TMEM65-V5 and TMEM65 which forms a complex in brain tissue.

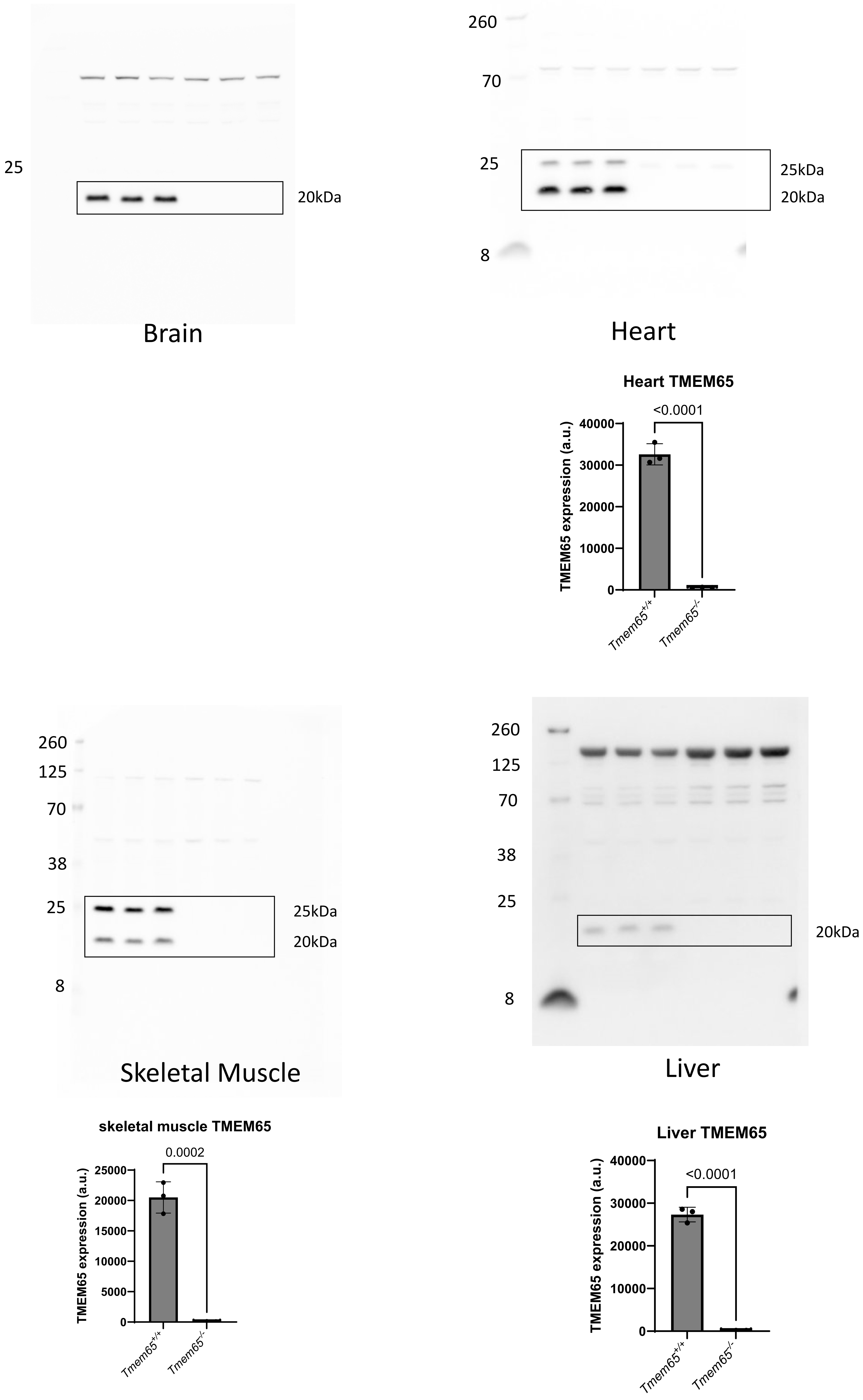

Supplementary Figure 12: Western blots of Figure 1d with anti-TMEM65 antibody.

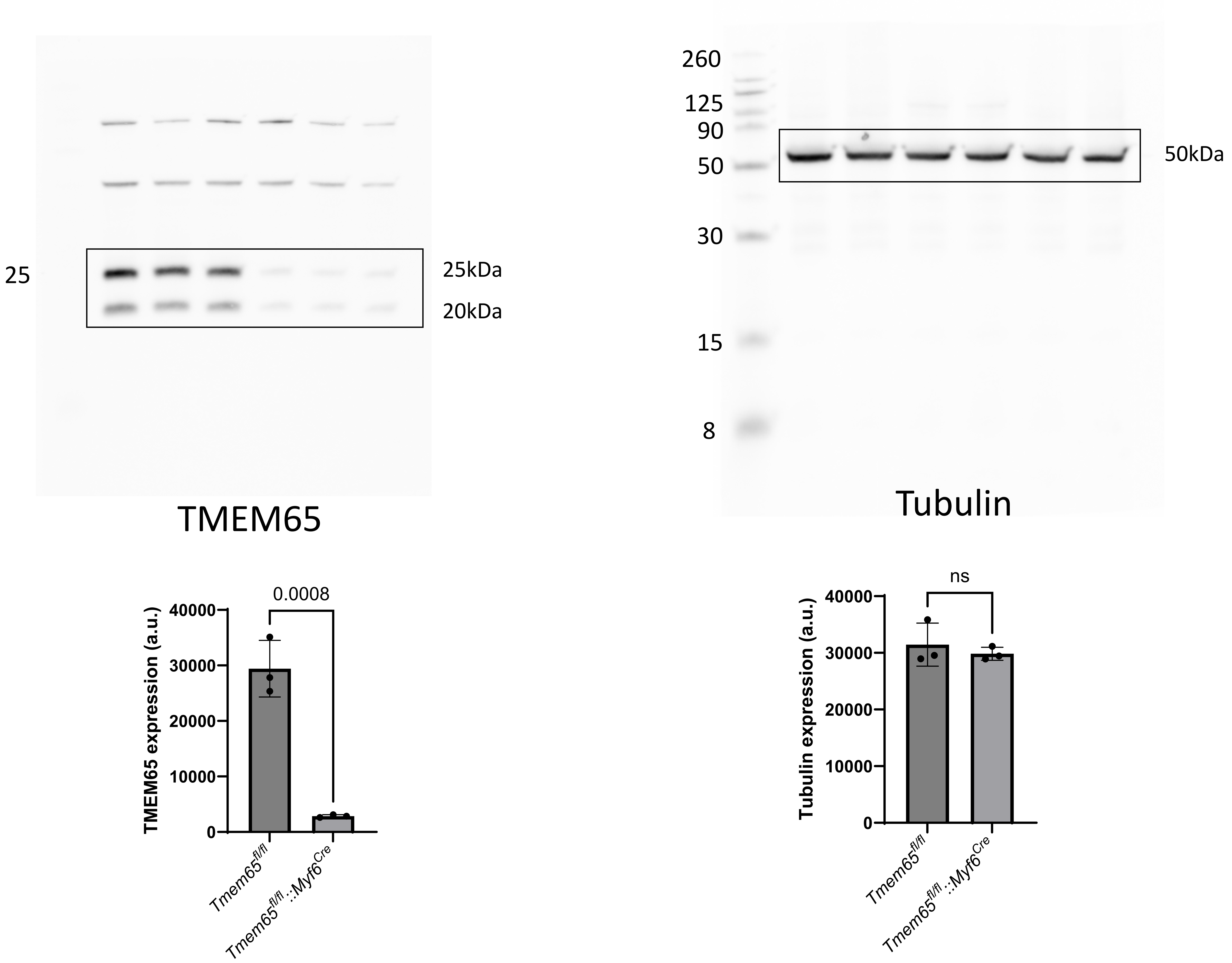

Supplementary Figure 13: Western blots and quantification of Figure 3a

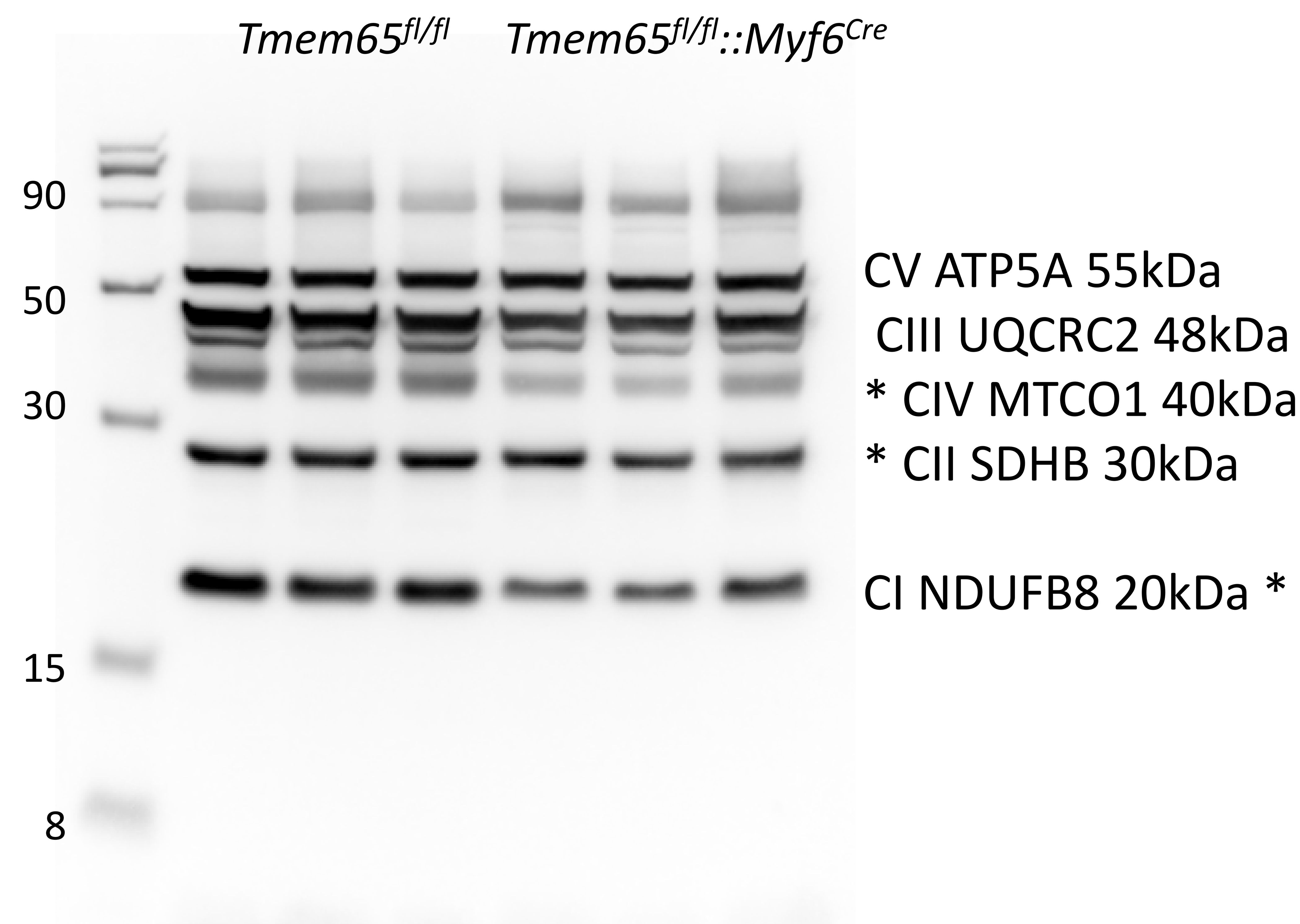

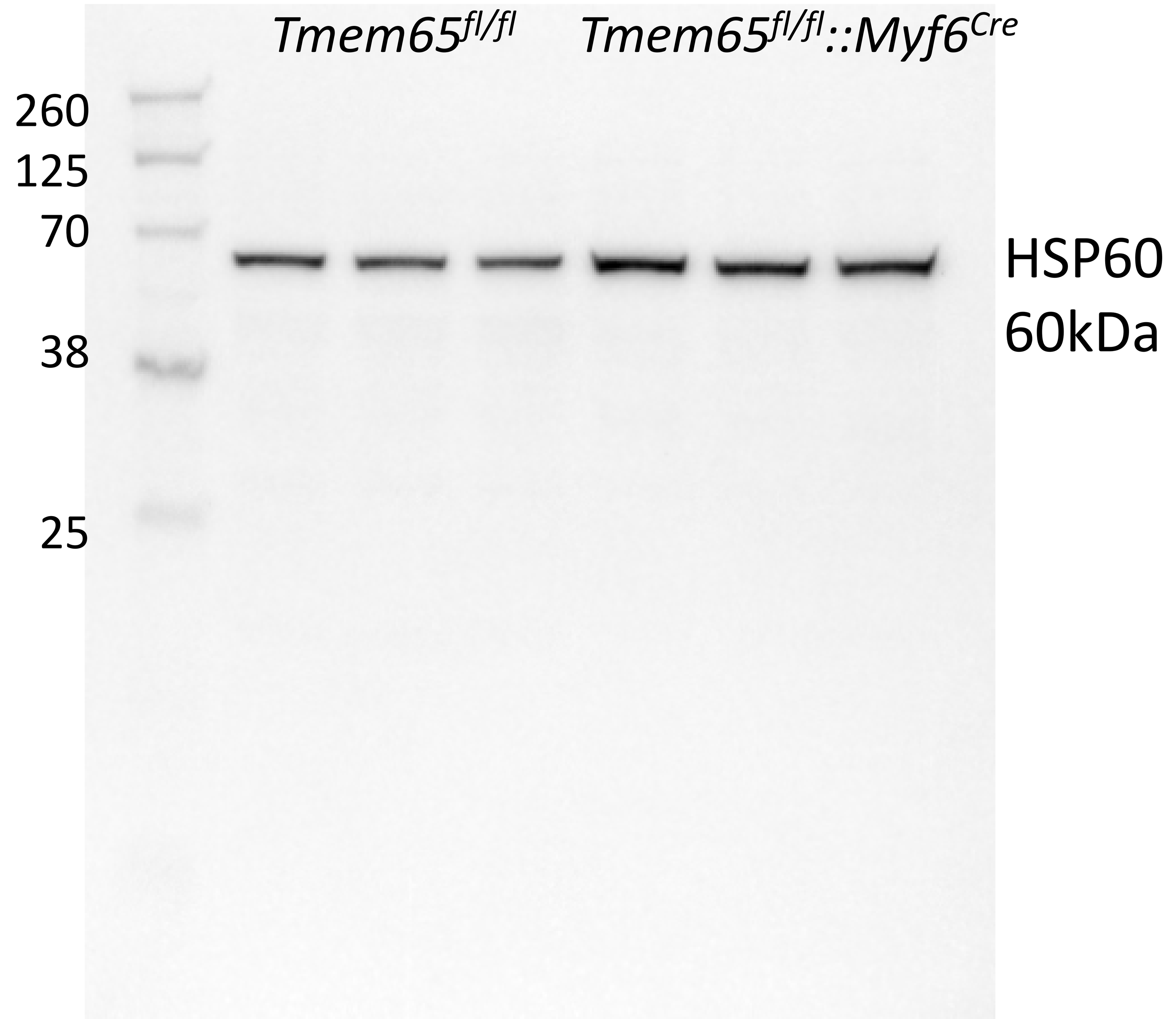

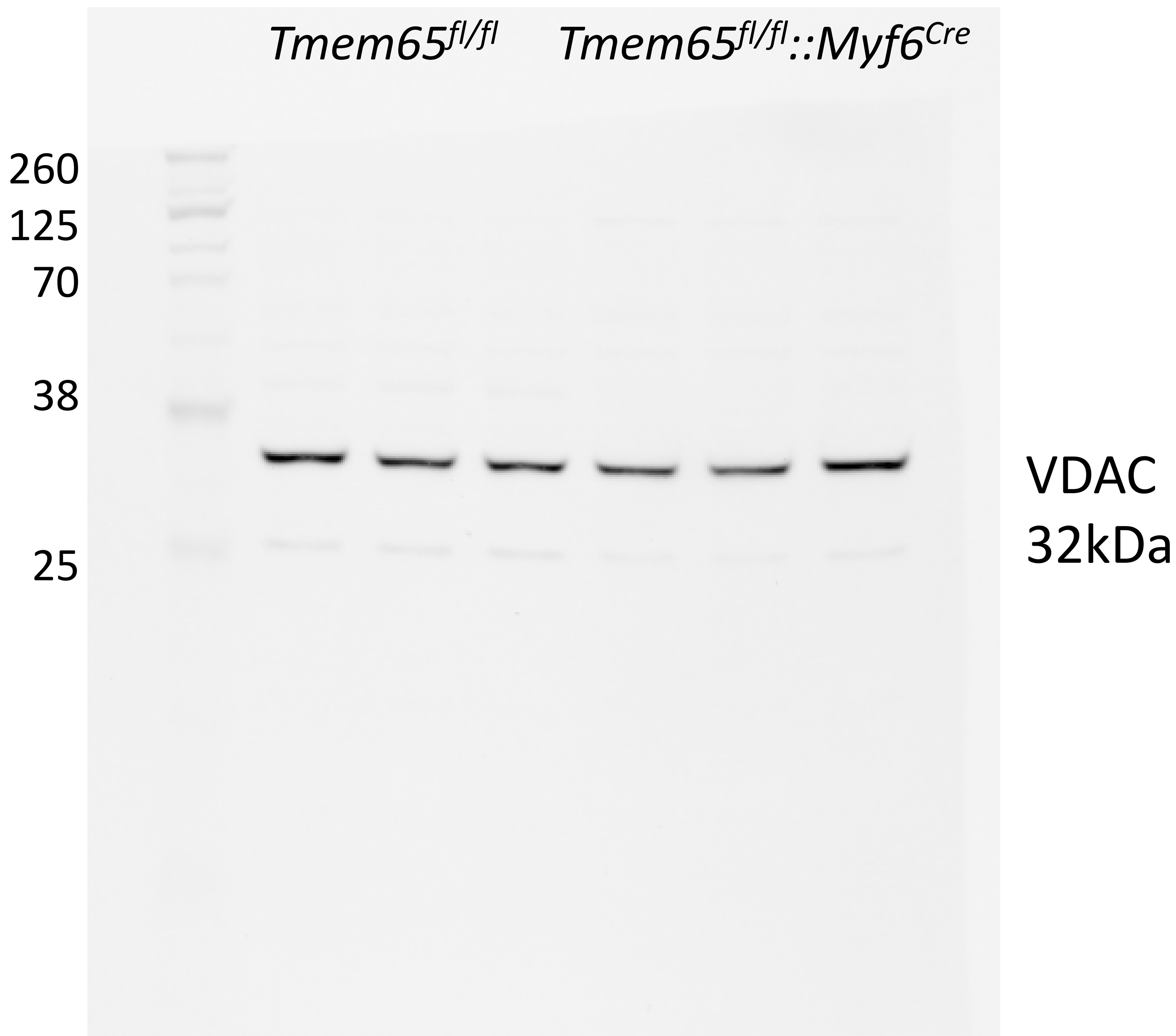

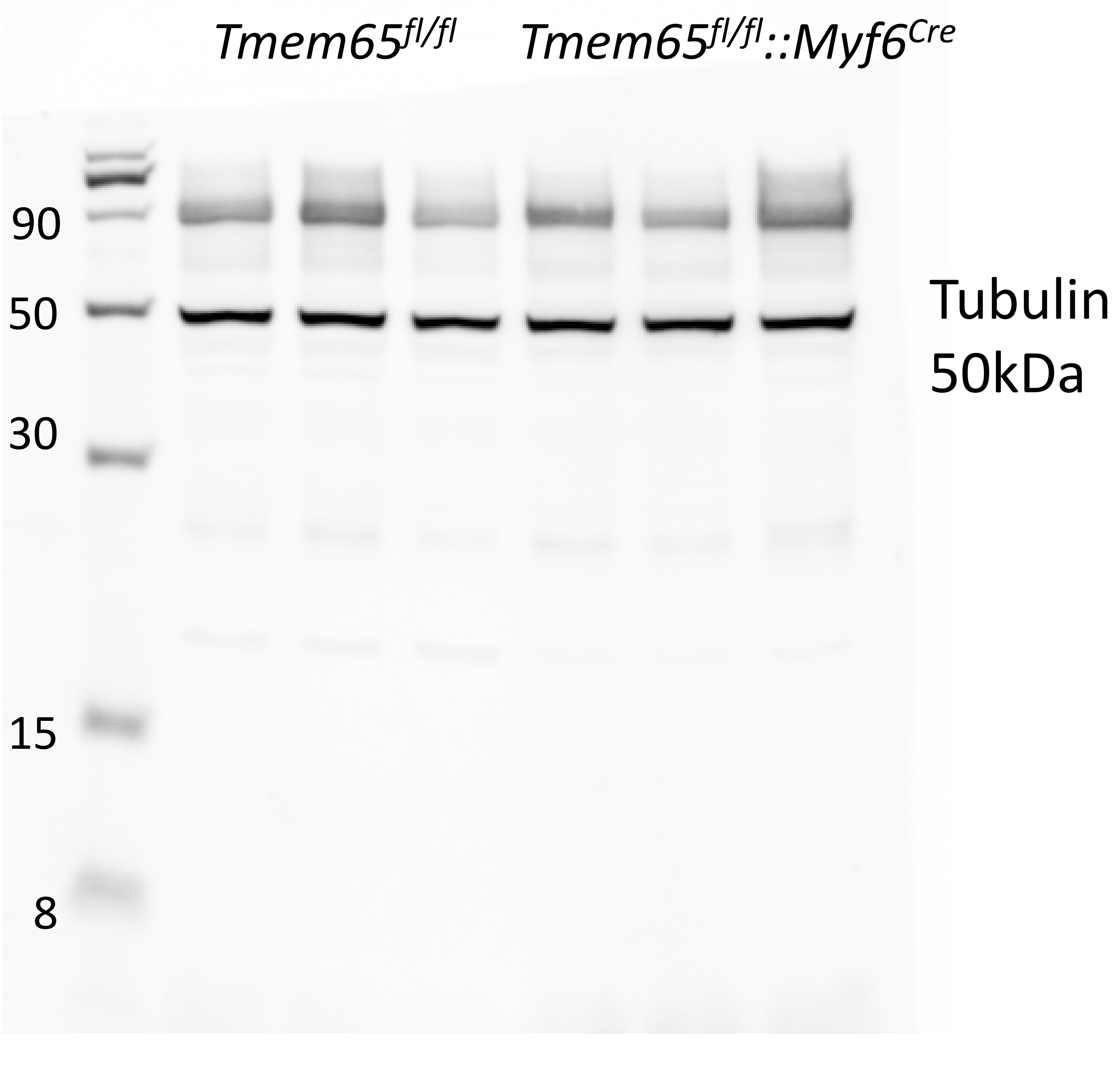

Supplementary Figure 14: Western blots of Figure 4g

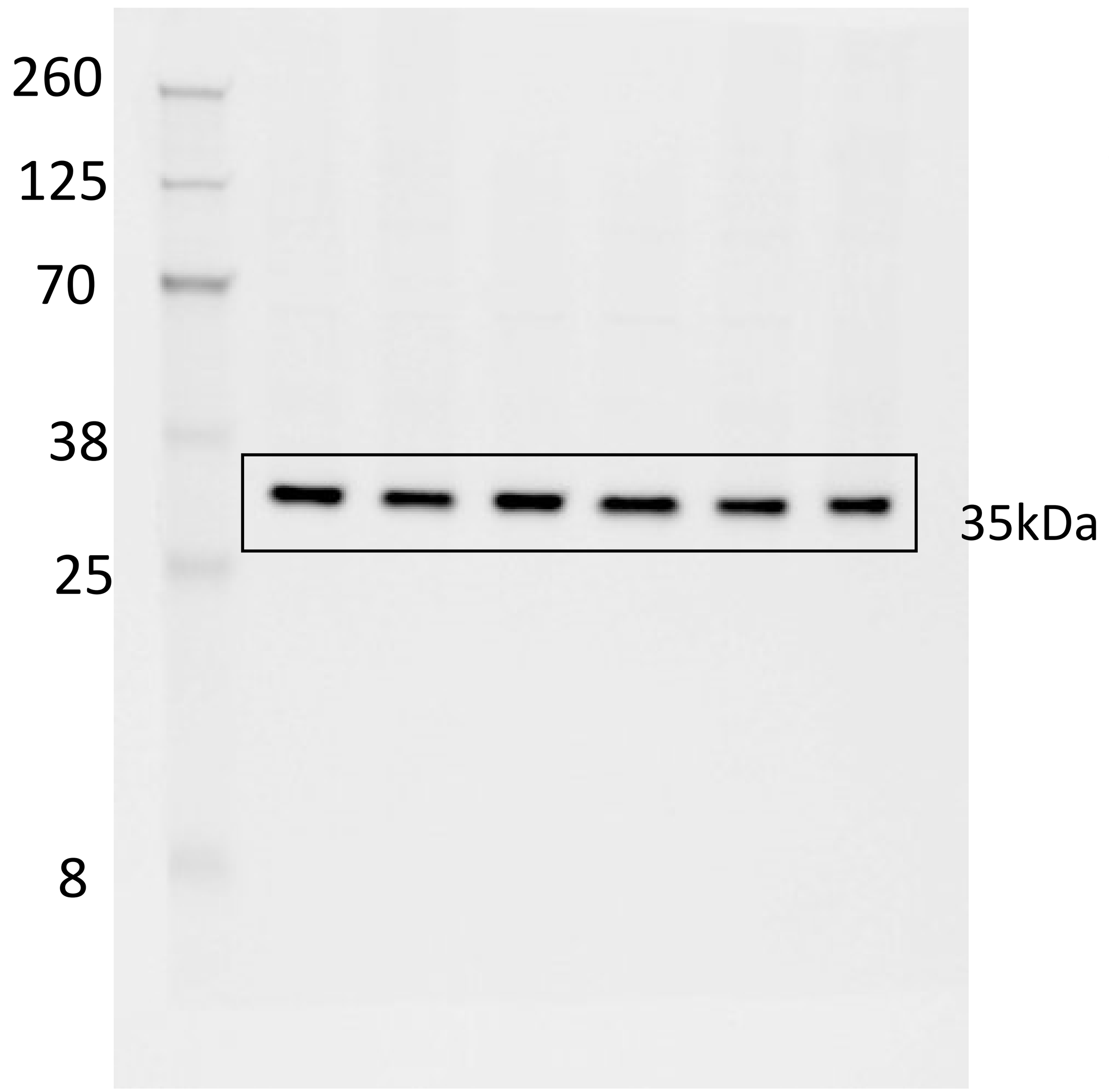

MCU

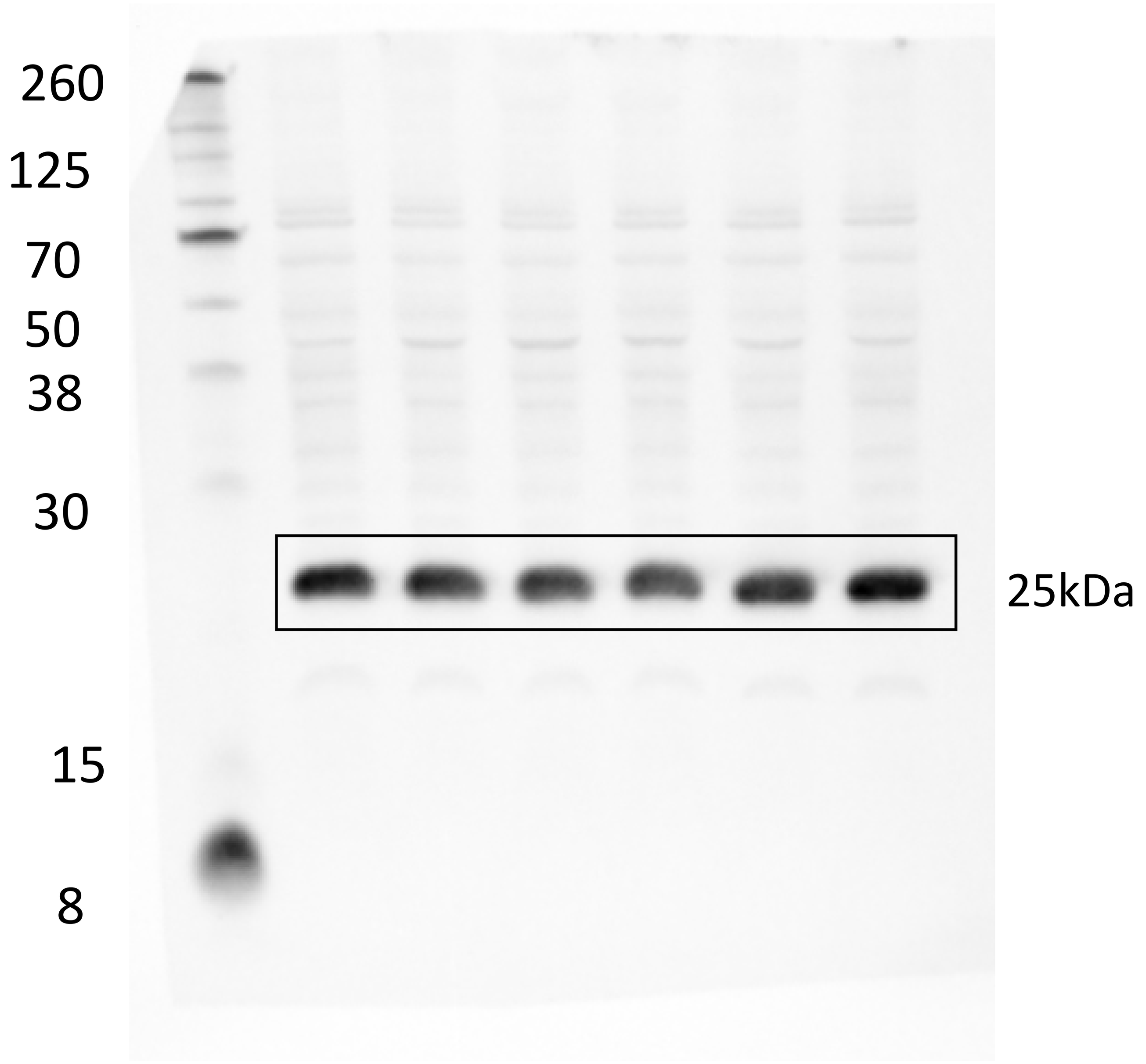

NCLX

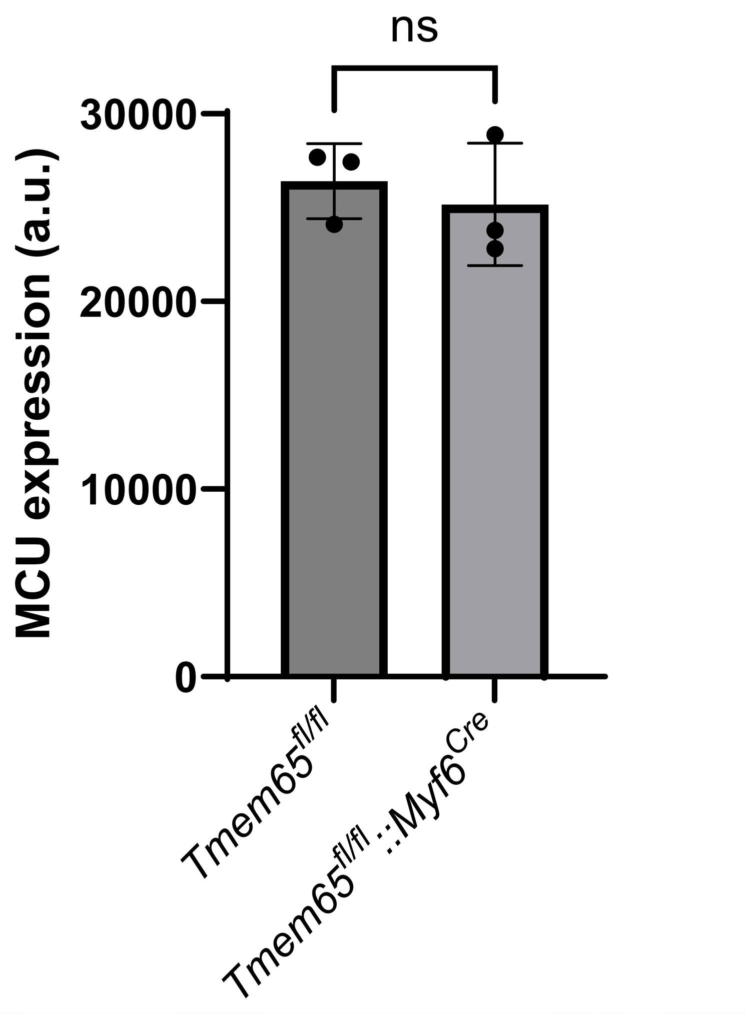

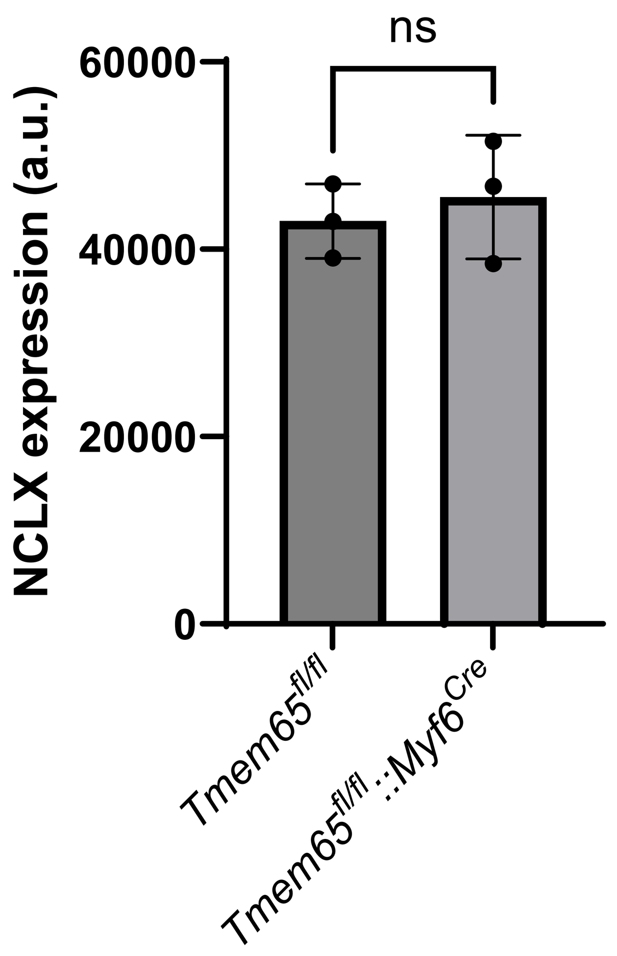

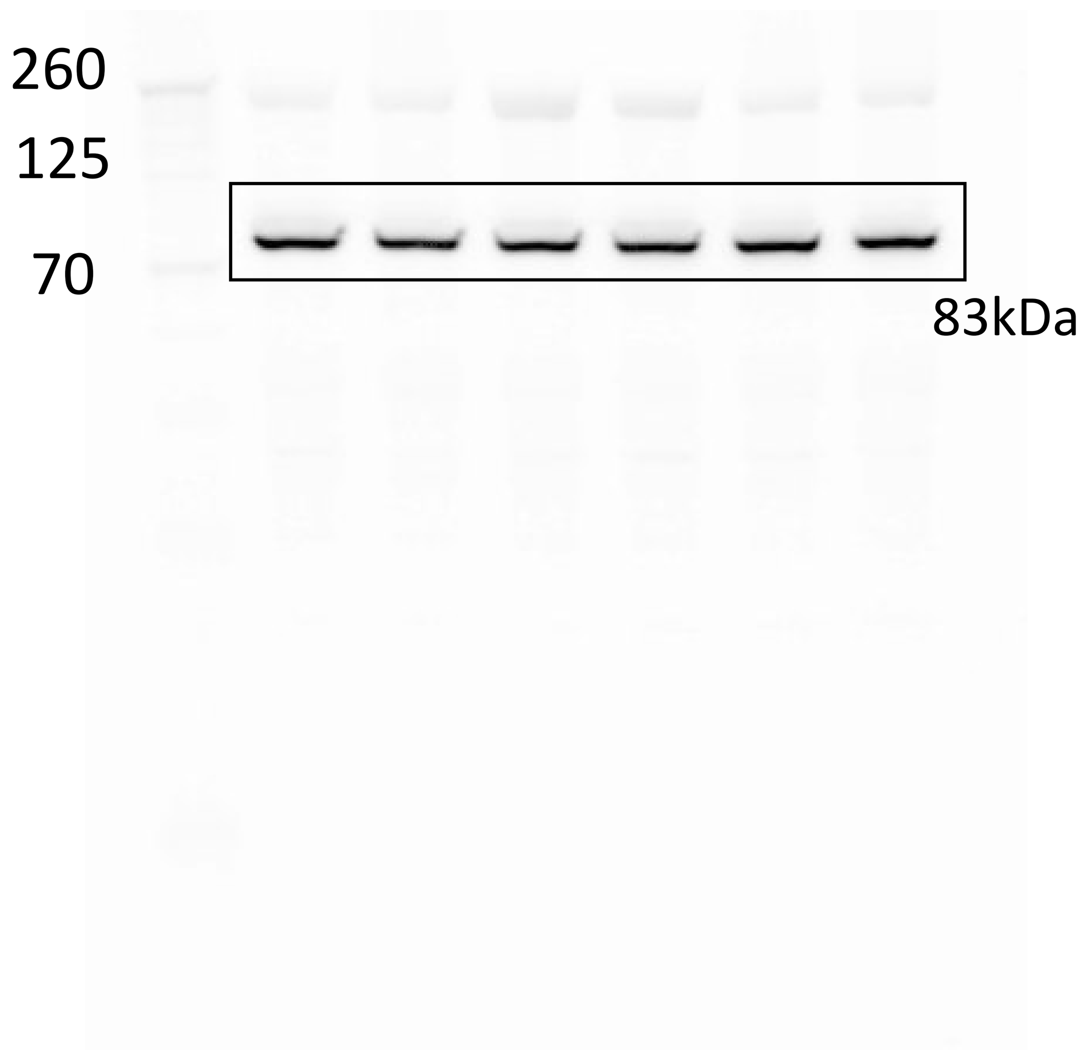

LETM1

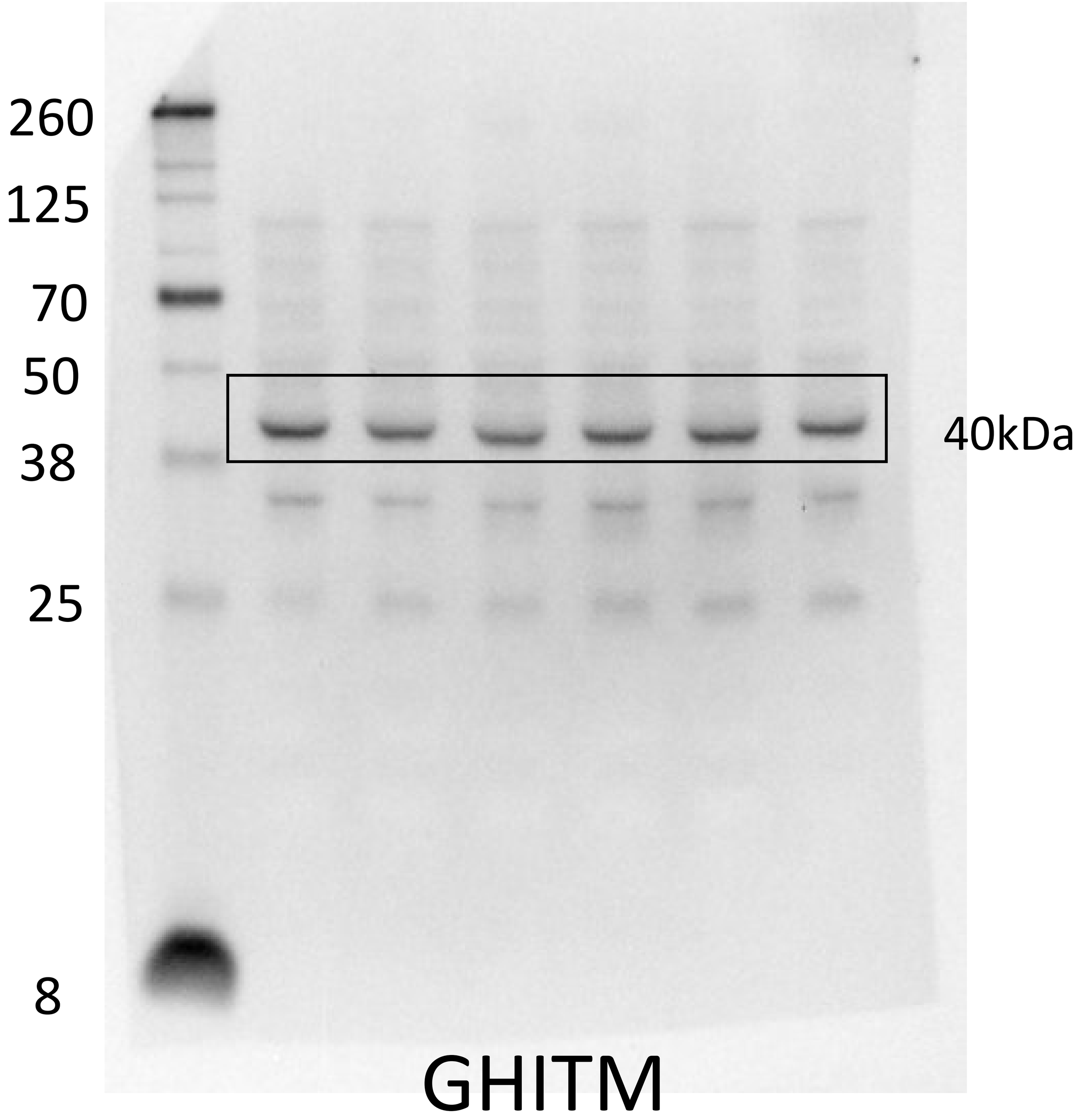

GHITM

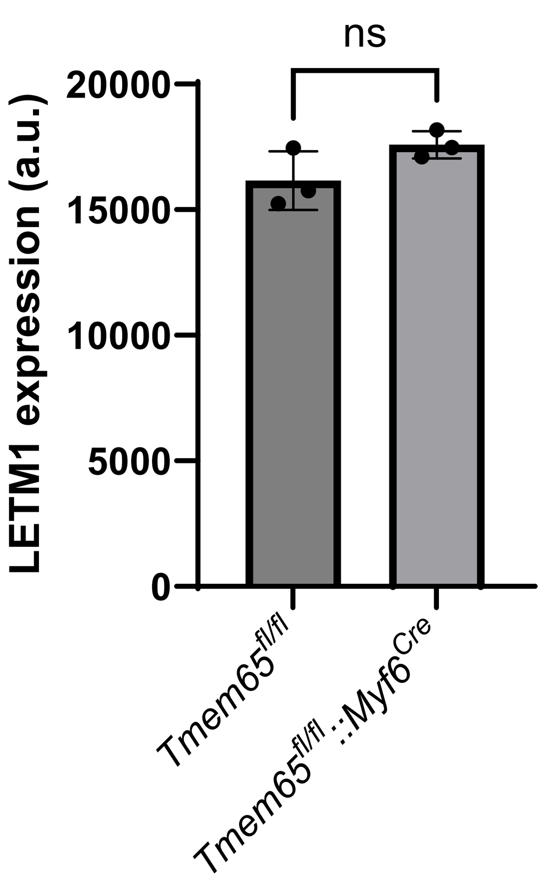

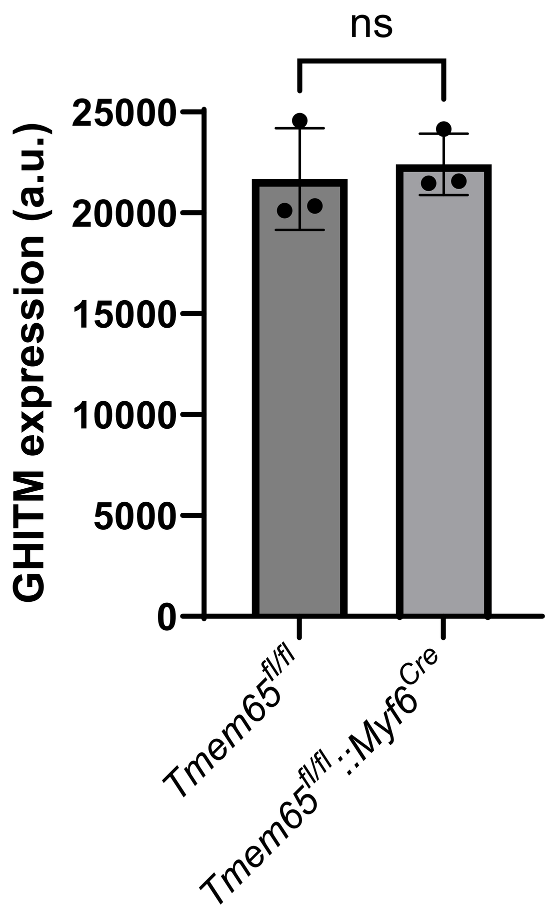

Supplementary Figure 15: Western blots and quantification of Figure 5m

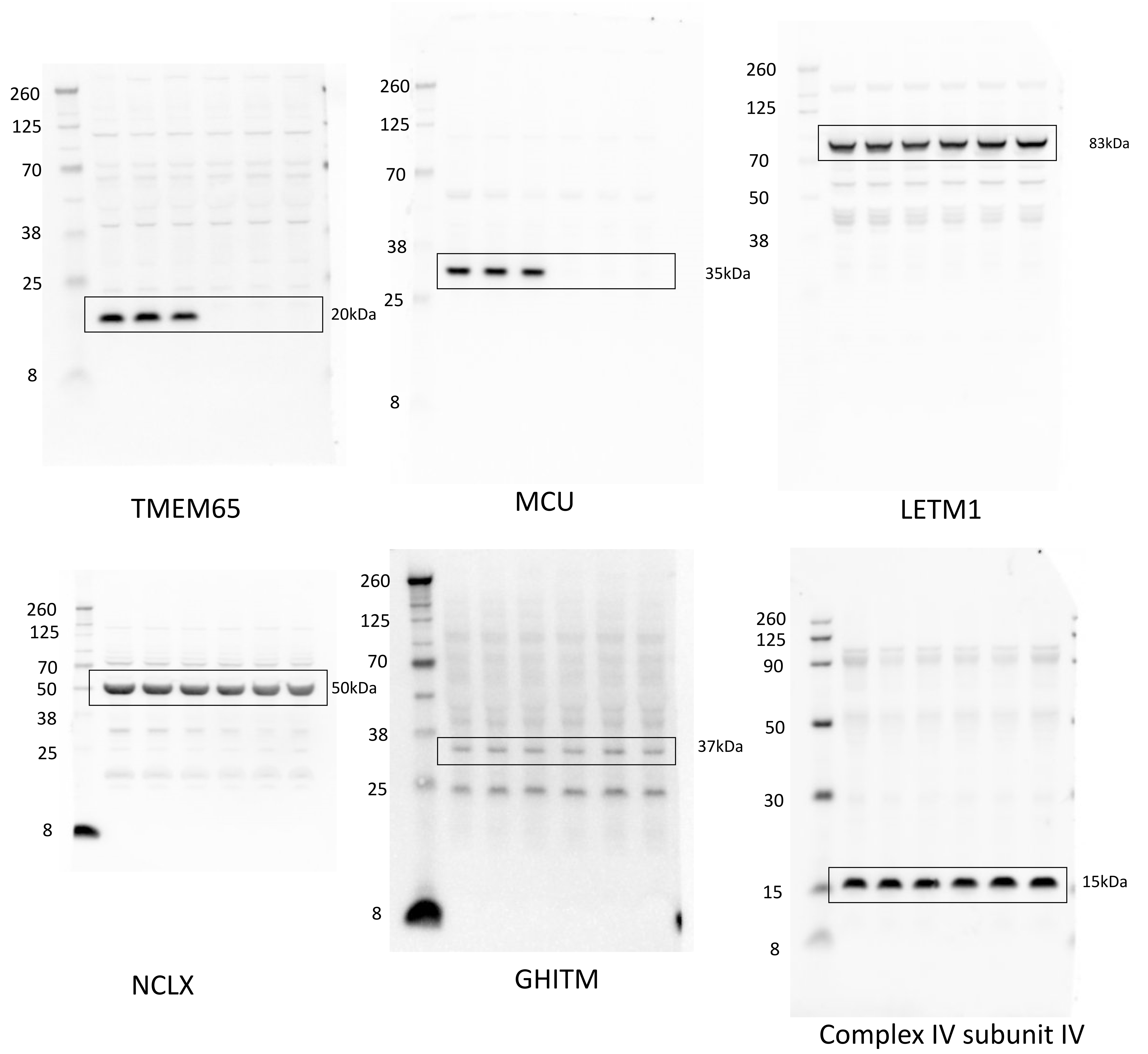

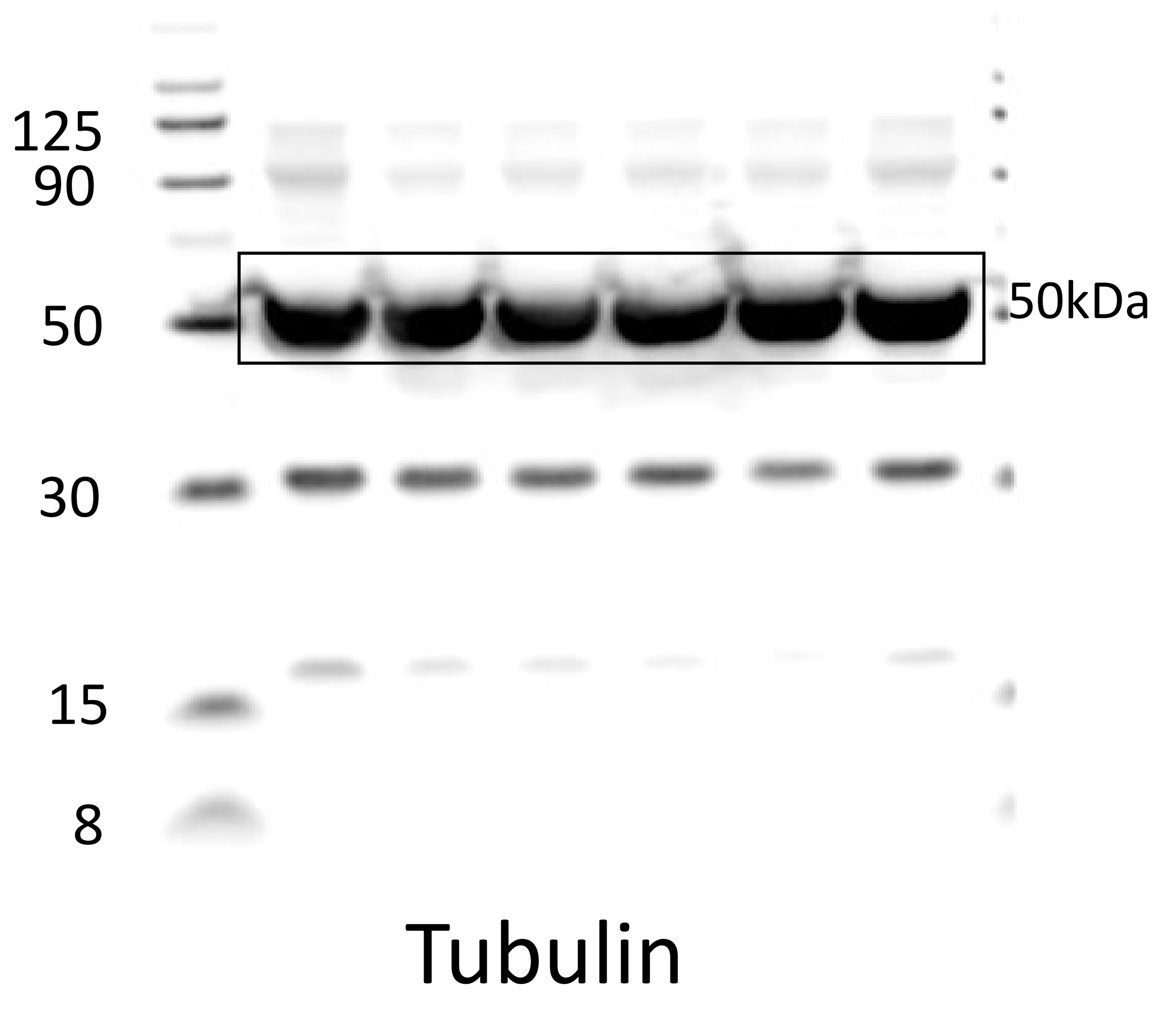

Supplementary Figure 16: Western blots of Figure 6a.

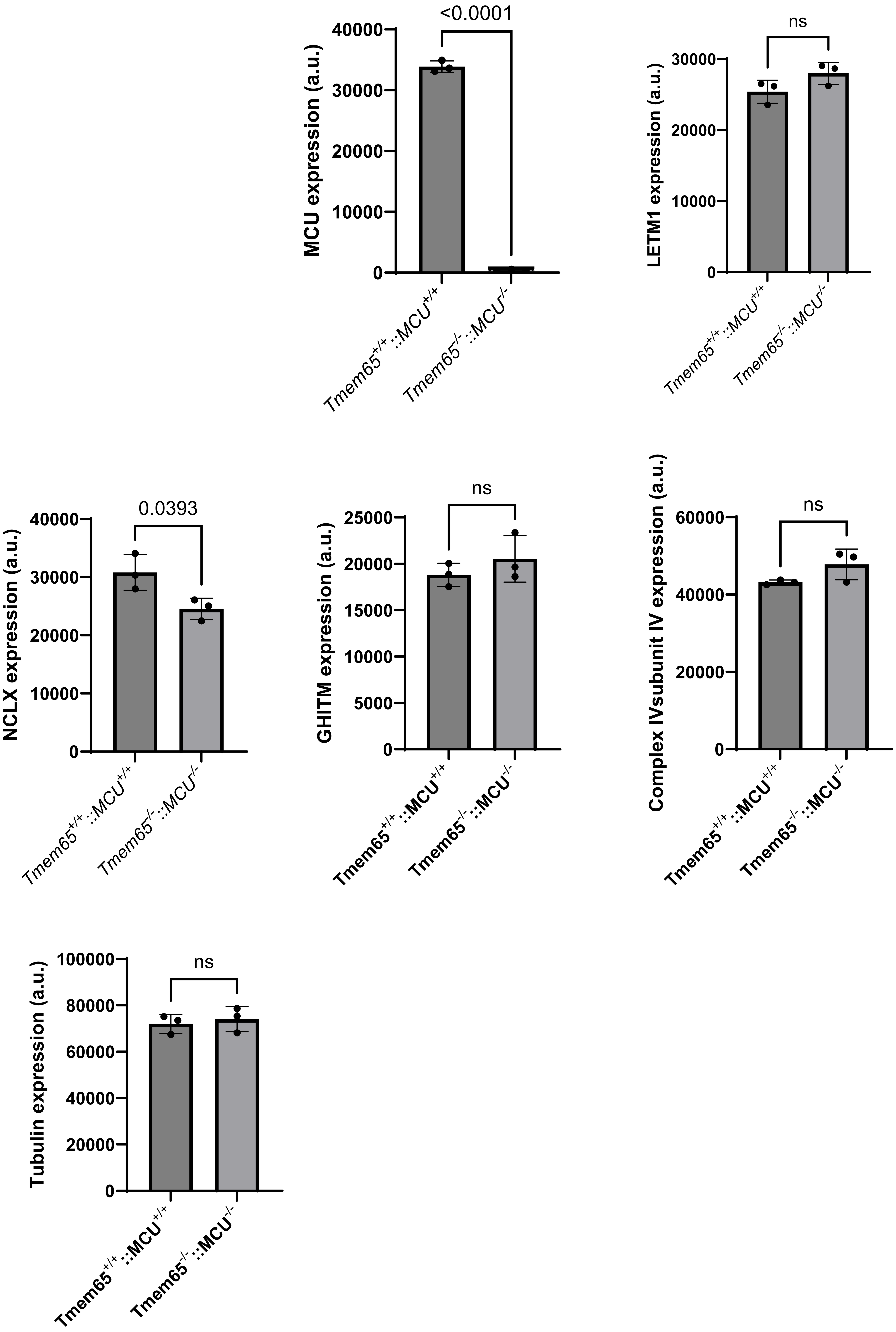

Supplementary Figure 17: Quantification: Western blots of Figure 6a.

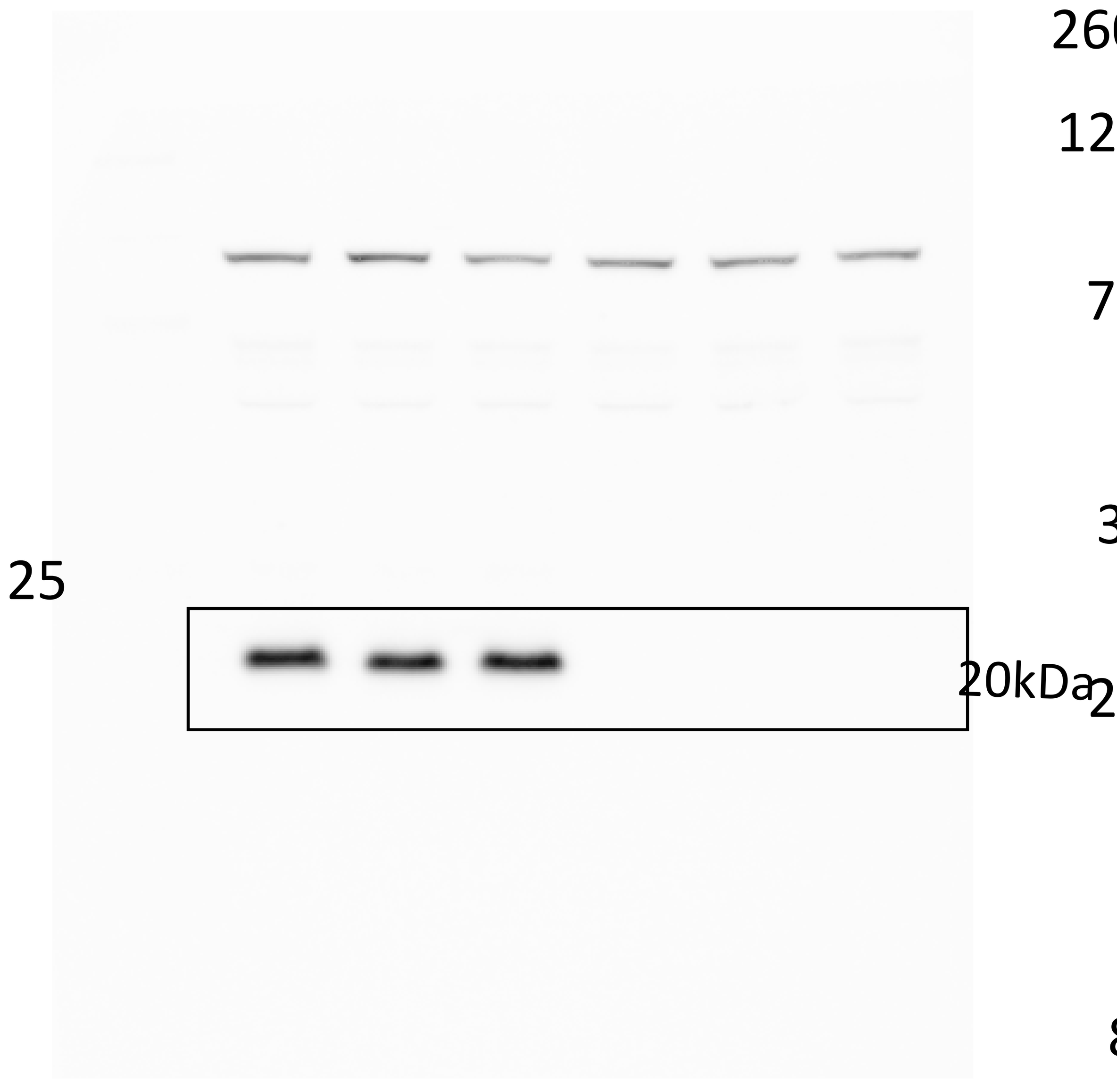

TMEM65

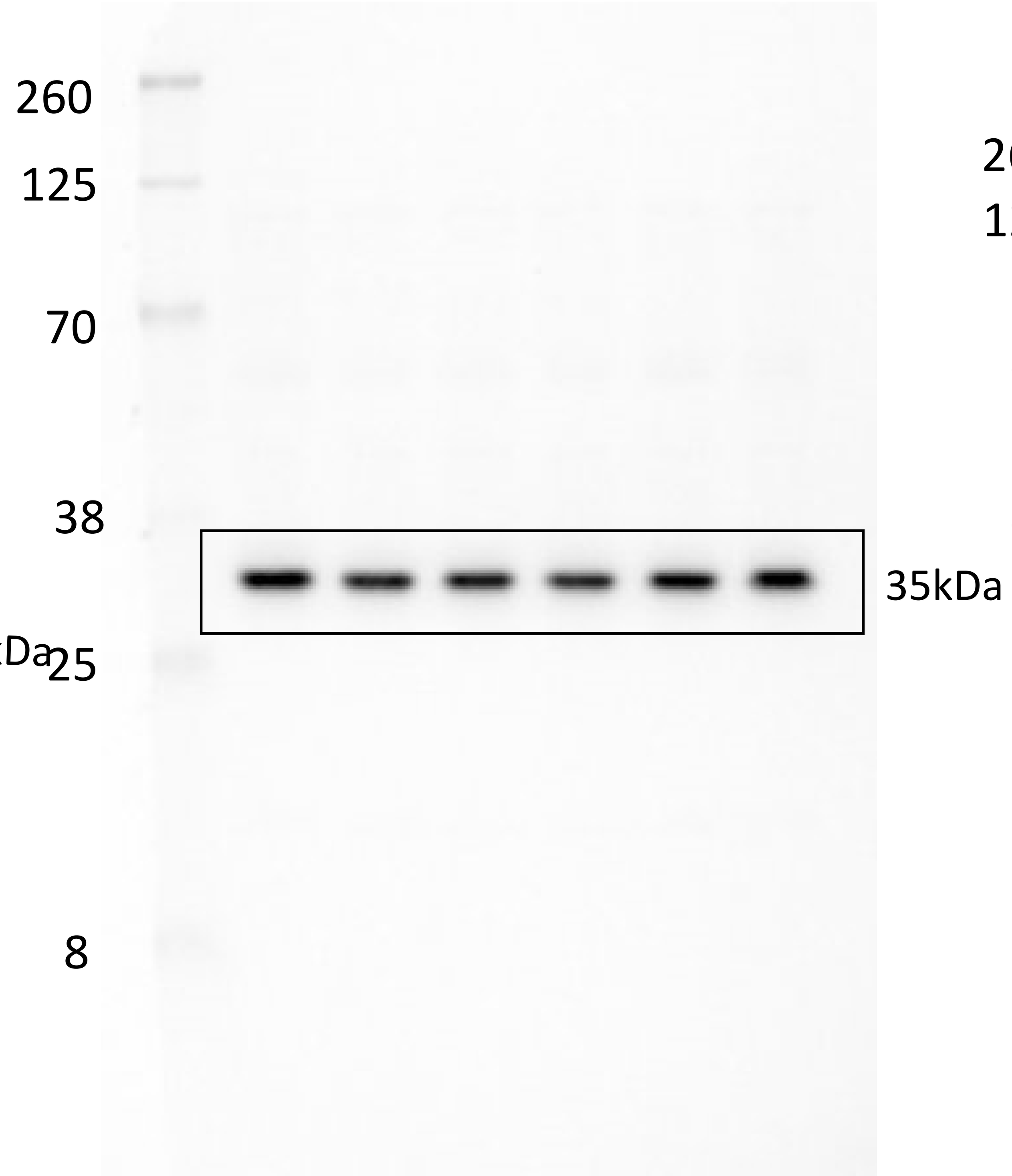

MCU

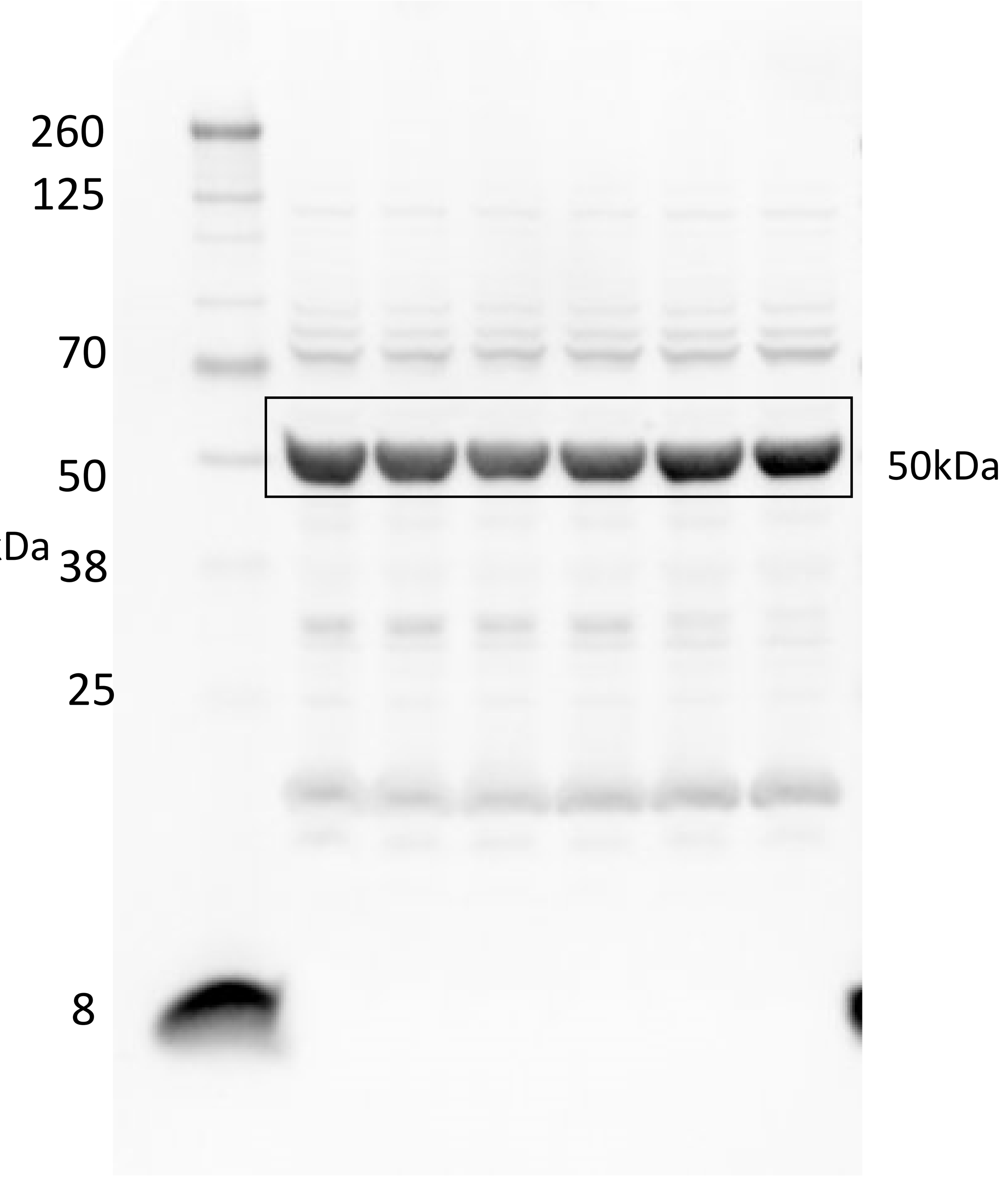

NCLX

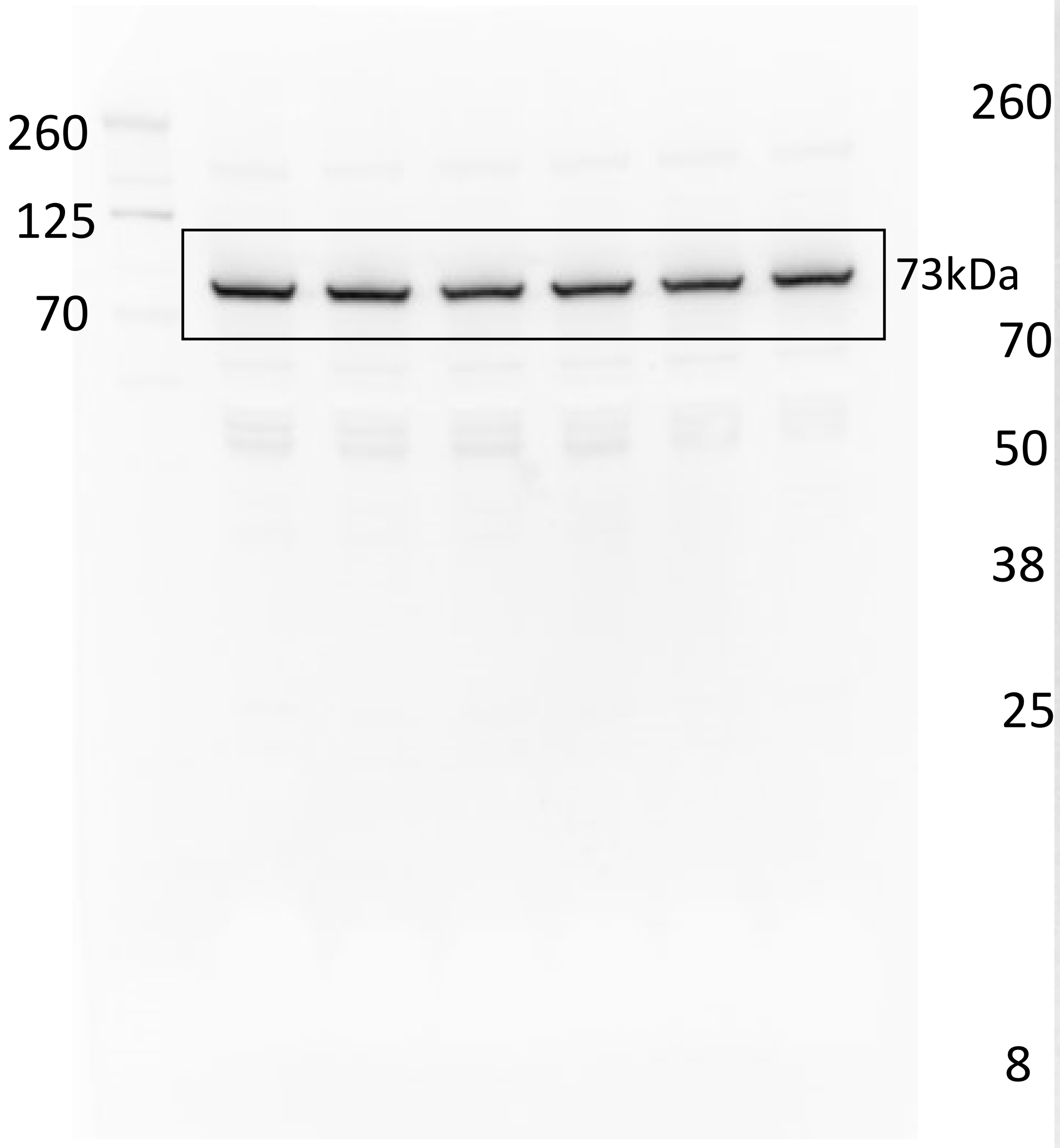

LETM1

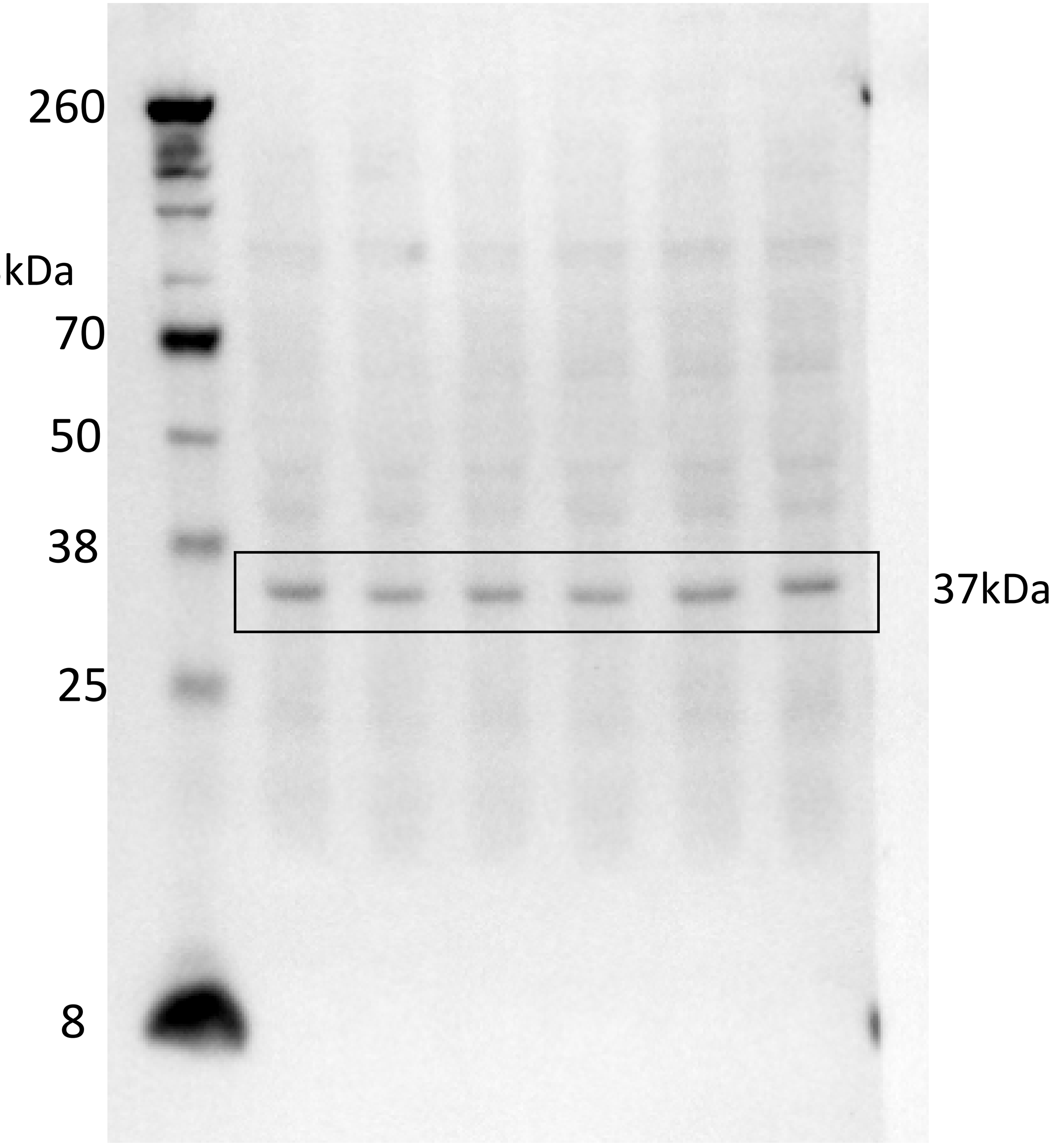

GHITM

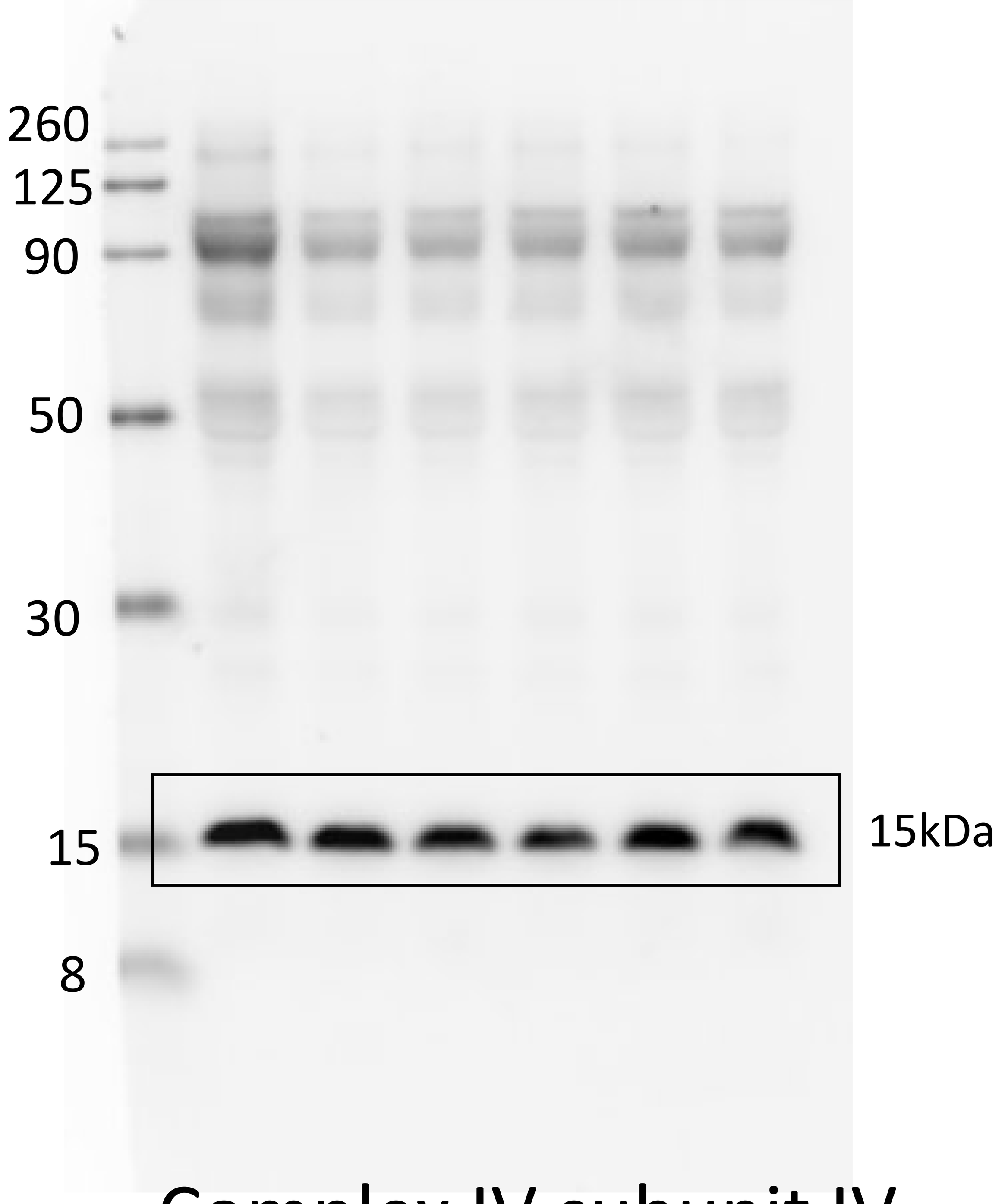

Complex IV subunit IV

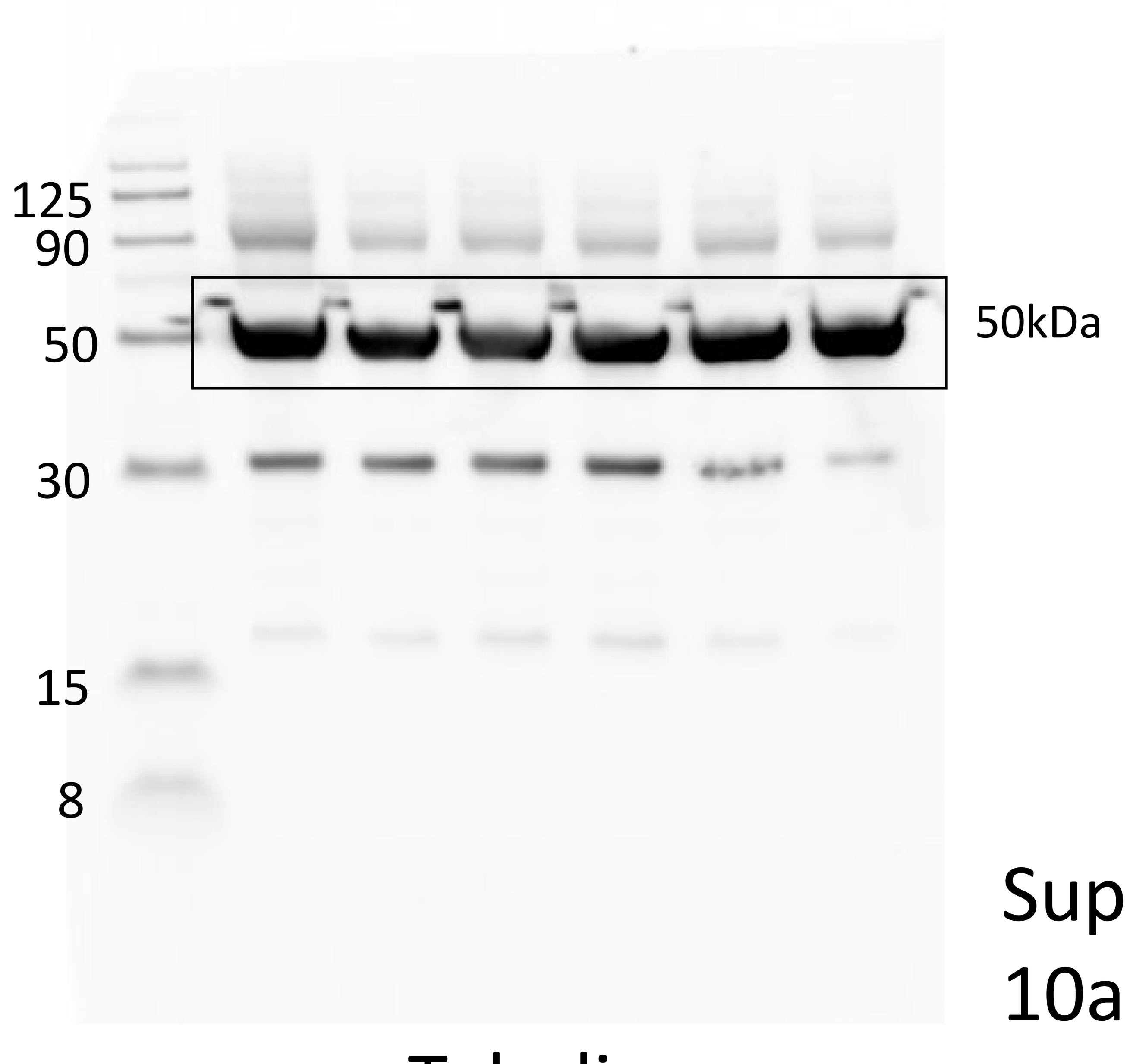

Tubulin

Supplementary Figure 18: Western blots of Extended Data Figure 10a

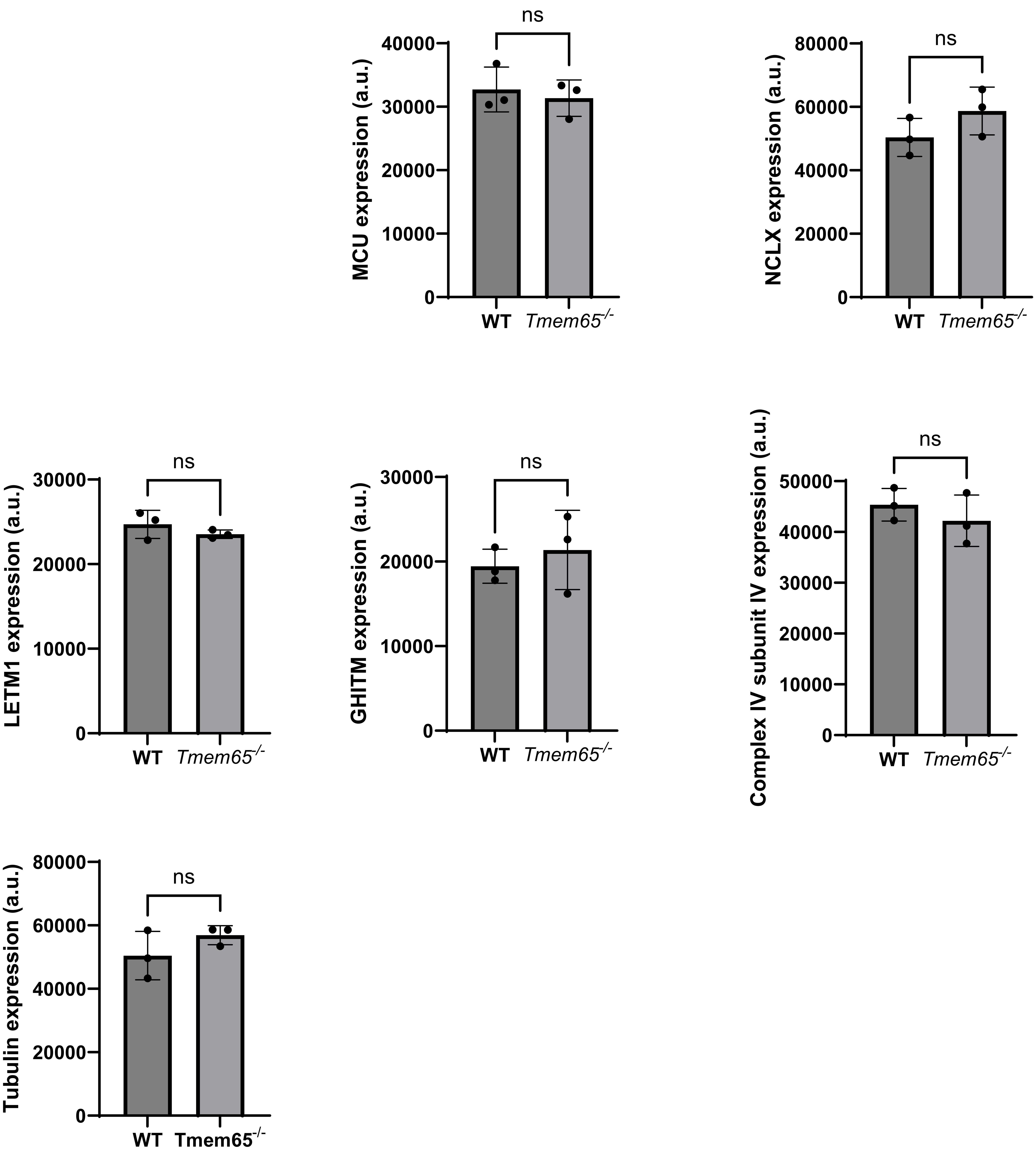

Supplementary Figure 19: Quantification: Western blots of Extended Data Figure 10a

Genotype	Number of pups	Percentage
Tmem65$^{+/+}$	65	26.7%
Tmem65$^{+/-}$	121	49.8%
Tmem65$^{-/-}$	57	23.5%
	243 (total)	100% (total)

Supplemental Table 1: Genotyping results of TMEM65 KO mouse line from pups at P5. TMEM65 KO mice were at expected Mendelian ratios.

Inactive*	7	77.8%
Pulling of hind limbs	3	33.3%
Uncoordinated walking	6	66.7%
Seizure before death	9	100%

Supplemental Table 2: Summary of phenotypes of *Tmem65*$^{-/-}$ mice before death based on recordings of 9 *Tmem65*$^{-/-}$ mice. *Inactive indicates that mouse did not move anywhere other than between the nest and the food source.
