# Supplementary material for: Loss of TMEM65 in mice causes mitochondrial disease mediated by mitochondrial Ca2+
Source: Nat Commun. 2026 Apr 14;17:5203. doi: 10.1038/s41467-026-71761-w (PMC13254257; doi:10.1038/s41467-026-71761-w)
Supplement: Supplementary file 2 — Description of Additional Supplementary Files [file 41467_2026_71761_MOESM2_ESM.pdf]

Description of Additional Supplementary Files

Supplementary Data 1: Protein abundances for soleus muscles of wildtype and muscle-specific Tmem65 knockout mice at 2 months and 10 months of age.

Supplementary Movie 1: Video cage monitoring of whole body Tmem65 knockout mouse showing seizures immediately prior to death.
