# Supplementary material for: Loss of TMEM65 in mice causes mitochondrial disease mediated by mitochondrial Ca2+
Source: Nat Commun. 2026 Apr 14;17:5203. doi: 10.1038/s41467-026-71761-w (PMC13254257; doi:10.1038/s41467-026-71761-w)
Supplement: Supplementary file 6 — Transparent Peer Review file [file 41467_2026_71761_MOESM6_ESM.pdf]

nature portfolio

Peer Review File

Loss of TMEM65 in mice causes mitochondrial disease mediated by mitochondrial Ca^{2+}

Corresponding Author: Dr Brian Glancy

This manuscript has been previously reviewed at another journal. This document only contains information relating to versions considered at Nature Communications.

This file contains all reviewer reports in order by version, followed by all author rebuttals in order by version.

Version 0:

Reviewer comments:

Reviewer #1

(Remarks to the Author)
The authors have answered the reviewers' concerns. I recommend publication.

Reviewer #2

(Remarks to the Author)
This manuscript provides a thorough characterization of TMEM65KO (whole body or tissue specific). The authors also rescue TMEM65KO phenotype by MCU KO, providing MCU as a proof-of-concept therapeutic target for patients affected by TMEM65 loss.

My main issue with the manuscript was the biochemistry the authors showed on TMEM65 and NCLX interaction. Although I think the controversy still exists, since Tsai and Elrod groups published biochemically and functionally opposite results (functional dependence and physical interaction between the two or lack of thereof), I think this manuscript is not well poised to solve the controversy.

However, I do feel strongly that the authors should remove the data shown in Suppl. Figure 11b, to avoid claiming that an antibody that is not validated for BN-PAGE works for BN-PAGE, and to claim that NCLX is observed in 3 different bands (480kDa or higher). Tsai paper Extended Data 4a and 4b BN-PAGE data using tagged protein do not agree with these sizes. It is likely that the NCLX bands shown in Suppl. Figure 11b are nonspecific bands.

Reviewer #4

(Remarks to the Author)
In all Figures: Why are P values given to 4 decimals? This doesn’t seem needed.

In many Figures: Because sometimes 2 time points were investigated, it is highly recommended to indicate “age” within the figure panel (eg in Figure 3).

Major Comments:
Figure 3
Figure 3I Energy expenditure data: Two points. 1) Please show VO2 and VCO2 separately. 2) Please (also) show data without dividing by body weight. If body composition changes, which seems the case (Extended Data Fig7, with disproportionately greater drop in adipose tissue than lean mass), then dividing through by BW is not meaningful.

Figure 4
Panel H: Given that panel H displays quantification of panel G data, Panel H x-axis labels should read the actual protein for

which the antibody (eg CIII UQCRC2) was used and not Complex I etc. The reason is that a change in a subunit of an ETC complex does not necessarily mean that the expression of the assembled complex is changes. Therefore, using Complex I etc in this context is misleading.
Regarding the interpretation of JO2 data in Fig4 (from frozen lysate – is this correct?), Extended Data Fig10 (delta psi in mitos and muscle), I need to disagree with the Authors' interpretation. First, determining JO2 in frozen muscle sure underestimates the maximal oxphos. Second, the single-condition/time point delta psi (rel to mitotracker) has too much imprecision to make an accurate comparison; the data are highly variable (not necessarily a problem) and there is no sense of the dynamic range of the measurements: what is the fully depolarized level? Is it possible to observe some hyperpolarization using oligomycin? I also don't think you can conclude that ETC capacity is decreased based on wb in Fig4H; at the very least, one should look at expression of the assembled complexes (BN-PAGE). The indirect calorimetry data showing higher energy expenditure and food intake in muscle-kO mice is interesting (though it is not easy to explain what the underlie mechanism is, though Fig4G shows some tendency for elevated state 4 JO2 (though one needs to determine properly is this reflects increased proton leak)). There is an emerging concept that mitochondrial disease can be accompanied by "hypermetabolism" (Sturm et al 2023 PMID 36635485) and this could be another example; though it's not clear if this would apply here, since muscle-KO mice may feature disproportionately more lean mass vs adipose (the decrease in lean mass is less than the decrease in WAT: EDFig7).

Figure 5
Fig5B: The text on p8-9 describes Fig5B as data from the Rhod-2 signal. However Fig5B is the CRC experiment. Please correct this.
Fig5B CRC experiment. Data from Ming-Feng Tsai's and John Elrod's labs show decreased CRC when TMEM65 is absent. Fig5B shows no effect of TMEM65 ko. However, it took many 5 uM pulses to achieve mPTP. What would occur under conditions where mPTP open probability were higher? I think this should be checked, to better reconcile your results with those from the other 2 groups. Also, as far I could tell, the conditions of the CRC experiment were not described. Please described (which is especially important if results are/remain discordant.
Fig5LMN: Fig5L shows elevated uniporter components and GHITM in 10 mth old TMEM65 ko muscle. Fog Fig5MN, legend indicates that data are from 10 mth old mice; however, there is no evidence of elevated MCU and GHITM in the wb (panel MN). What is the reason for this discrepancy?
Fig5M: NCLX wb. Why at 25kDa. In Palty et al 2004, skel muscle band is shown at ~50 kDa. Palty et al show a band at ~35 kDa for heart, but heart also shows a (slightly stronger) band at ~50 kDa, as for skel muscle. At the very least, you should show a membrane for MW spanning ~65 down to 20 kDa, for readers to make their own assessment.

Figure 6
Panel K, vs. Extended data Fig7B – what can account for the decreased CSA in KO muscle shown in EDFig7B vs. the increase shown in Fig6K? More generally, I have a concern with the interpretation of some of the double KO data in Fig6: data in Fig6K and also in Fig6J suggests that MCU KO has a predominant phenotype that is not rescued by TMEM65 KO. That would be my interpretation. I would not say that these data are revealing something primarily about TMEM65. Where are data from TMEM65-/-::MCU+/+ muscle? I think that that would be more informative. I don't think the data should be omitted. But, perhaps these data could be relocated to the supplement?

Version 1:

Reviewer comments:

Reviewer #2

(Remarks to the Author)
The authors addressed all of my concerns. This manuscript adds to the growing body of literature on the role of TMEM65 in mitochondrial calcium handling, metabolism and health.

Reviewer #4

(Remarks to the Author)
Thank you for addressing all of my concerns, and clearly better explaining certain aspects of experimental design and interpretation in your response.

Open Access This Peer Review File is licensed under a Creative Commons Attribution 4.0 International License, which permits use, sharing, adaptation, distribution and reproduction in any medium or format, as long as you give appropriate credit to the original author(s) and the source, provide a link to the Creative Commons license, and indicate if changes were made.
In cases where reviewers are anonymous, credit should be given to 'Anonymous Referee' and the source.
The images or other third party material in this Peer Review File are included in the article's Creative Commons license, unless indicated otherwise in a credit line to the material. If material is not included in the article's Creative Commons license and your intended use is not permitted by statutory regulation or exceeds the permitted use, you will need to obtain permission directly from the copyright holder.
To view a copy of this license, visit https://creativecommons.org/licenses/by/4.0/

Reviewer #1 (Remarks to the Author):

The authors have answered the reviewers' concerns. I recommend publication.

Reviewer #2 (Remarks to the Author):

This manuscript provides a thorough characterization of TMEM65KO (whole body or tissue specific). The authors also rescue TMEM65KO phenotype by MCU KO, providing MCU as a proof-of-concept therapeutic target for patients affected by TMEM65 loss.

My main issue with the manuscript was the biochemistry the authors showed on TMEM65 and NCLX interaction. Although I think the controversy still exists, since Tsai and Elrod groups published biochemically and functionally opposite results (functional dependence and physical interaction between the two or lack of thereof), I think this manuscript is not well poised to solve the controversy.

However, I do feel strongly that the authors should remove the data shown in Suppl. Figure 11b, to avoid claiming that an antibody that is not validated for BN-PAGE works for BN-PAGE, and to claim that NCLX is observed in 3 different bands (480kDa or higher). Tsai paper Extended Data 4a and 4b BN-PAGE data using tagged protein do not agree with these sizes. It is likely that the NCLX bands shown in Suppl. Figure 11b are nonspecific bands.
We have now removed this figure from the paper.

Reviewer #4 (Remarks to the Author):

In all Figures: Why are P values given to 4 decimals? This doesn't seem needed.

We thank the reviewer for this question. In several instances, we show that $p < 0.0001$ to demonstrate the level of significance. To be consistent, all p-values are shown to the same level of precision in the main figures.

In many Figures: Because sometimes 2 time points were investigated, it is highly recommended to indicate "age" within the figure panel (eg in Figure 3).

We thank the reviewer for this comment. We have now indicated age for the figure panels in Figures 3-5 when more than one age group is used.

Major Comments:
Figure 3
Figure 3I Energy expenditure data: Two points. 1) Please show VO2 and VCO2 separately. 2) Please (also) show data without dividing by body weight. If body composition changes, which seems the case (Extended Data Fig7, with disproportionately greater drop in adipose tissue than lean mass), then dividing through by BW is not meaningful.

We thank the reviewer for these points. We have now shown VO2 and VCO2 separately in Extended Data Figure 7d. For the second point, we would argue that showing energy expenditure relative to body weight is correct in this case. The assessment of work was measured as daily mobility which requires moving the animal's body weight. Thus, the lower body weight in the KO mice means less work performed per unit of mobility. In order to properly assess metabolic efficiency here (i.e. energy expenditure per unit work performed), body weight needs to be accounted for in the energy expenditure value.

If we used absolute energy expenditure as suggested, we would have to multiply daily mobility by body weight to make that measure representative of absolute work performed. Thus, both energy expenditure and work would be multiplied by body weight compared to the current values and, since both the numerator and denominator would be multiplied by the same factor, the interpretation of metabolic efficiency would not change.

Figure 4
Panel H: Given that panel H displays quantification of panel G data, Panel H x-axis labels should read the actual protein for which the antibody (eg CIII UQCRC2) was used and not Complex I etc. The reason is that a change in a subunit of an ETC complex does not necessarily mean that the expression of the assembled complex is changes. Therefore, using Complex I etc in this context is misleading.

While the 10 month old proteome data does indeed suggest expression of the entire complex is reduced, the reviewer is correct that these 6 month old western blots only show representative proteins. Thus, we have added the specific protein labels to panel H (they were already included in panel G) as requested.

Regarding the interpretation of JO2 data in Fig4 (from frozen lysate – is this correct?), Extended Data Fig10 (delta psi in mitos and muscle), I need to disagree with the Authors'

interpretation. First, determining JO2 in frozen muscle sure underestimates the maximal oxphos.

We agree with the reviewer that using homogenates made from frozen muscles may underestimate maximal oxphos. However, we have no reason to believe that any potential loss of oxphos activity would be different between WT and KO tissues. Additionally, loss of ETC activity was previously shown in muscle biopsies and primary fibroblasts from a patient with a loss of Tmem65 activity, and this loss of activity was rescued by adding Tmem65 back to the cells (Nazli et al European Journal of Human Genetics 2017). Thus, our data are consistent with the previous literature, and we have now added this citation to the text here.

Second, the single-condition/time point delta psi (rel to mitotracker) has too much imprecision to make an accurate comparison; the data are highly variable (not necessarily a problem) and there is no sense of the dynamic range of the measurements: what is the fully depolarized level? Is it possible to observe some hyperpolarization using oligomycin?

We thank the reviewer for this comment. However, we assessed membrane potential in three different ways to ensure this was a robust measurement. In vivo Tibialis anterior muscle data using TMRM relative to the fully reduced NADH pool, in vitro isolated FDB muscle fibers data using TMRM relative to MitoTracker Green, and in vitro isolated hindlimb muscle mitochondria data using a TPP electrode all suggest there is no difference in membrane potential between WT and KO at two months of age. Additionally, we have previously shown in our in vivo system that addition of uncoupler will bring the mitochondrial TMRM signal down to background levels (Willingham et al Journal of Physiology 2019, Figure G) and TPP electrodes have been widely shown to be highly sensitive to changes in membrane potential including in our own work investigating the impact of calcium on oxidative phosphorylation (Glancy et al Biochemistry 2013). Thus, we are fully confident in these results.

I also don't think you can conclude that ETC capacity is decreased based on wb in Fig4H; at the very least, one should look at expression of the assembled complexes (BN-PAGE).

We conclude that ETC capacity is decreased in 6-10 month old muscle-specific Tmem65 KO mice based on proteomic analyses, western blot analyses, and functional analyses combined. Particularly, the proteomic results from 10-month old soleus muscles assess nearly all subunits of each complex and find Complex I (45 subunits assessed), Complex III (10 subunits assessed), and Complex IV (14 subunits assessed) are reduced in Tmem65 KO mice (individual subunit data available in Supplemental Dataset 1). Again, as mentioned above, the loss of ETC capacity reported here is

consistent the previous patient report (Nazli et al European Journal of Human Genetics 2017).

The indirect calorimetry data showing higher energy expenditure and food intake in muscle-kO mice is interesting (though it is not easy to explain what the underlie mechanism is, though Fig4G shows some tendency for elevated state 4 JO2 (though one needs to determine properly is this reflects increased proton leak)). There is an emerging concept that mitochondrial disease can be accompanied by "hypermetabolism" (Sturm et al 2023 PMID 36635485) and this could be another example; though it's not clear if this would apply here, since muscle-KO mice may feature disproportionately more lean mass vs adipose (the decrease in lean mass is less than the decrease in WAT: EDFig7).

We agree with the reviewer that this could be another case of hypermetabolism as defined by Sturm et al. However, since it is not clear, as the reviewer states, we have not added this to the paper .

Figure 5
Fig5B: The text on p8-9 describes Fig5B as data from the Rhod-2 signal. However Fig5B is the CRC experiment. Please correct this.

We thank the reviewer for catching this error which has now been corrected.

Fig5B CRC experiment. Data from Ming-Feng Tsai's and John Elrod's labs show decreased CRC when TMEM65 is absent. Fig5B shows no effect of TMEM65 ko. However, it took many 5 uM pulses to achieve mPTP. What would occur under conditions where mPTP open probability were higher? I think this should be checked, to better reconcile your results with those from the other 2 groups. Also, as far I could tell, the conditions of the CRC experiment were not described. Please described (which is especially important if results are/remain discordant.

We thank the reviewer for this comment. Both the Tsai and Elrod labs performed their CRC assays in Tmem65 KO cell lines while our CRC assays were performed in skeletal muscle mitochondria from two-month old, muscle-specific KO mice. As mitochondrial calcium handling capacities are tissue-specific (e.g. Rysted et al. Cell Calcium 2021), it is perhaps unsurprising that skeletal muscle mitochondria behave differently than mitochondria from cell lines. Indeed, the rapid change in cellular calcium associated with muscle contraction together with the relatively high activity of the mitochondrial calcium uniporter in skeletal muscle mitochondria compared to other tissues (Fieni et al Nature Communications 2012) would suggest a necessarily

high CRC in skeletal muscle. We have also now added additional details for the CRC method and cited the work it is based on (Pan et al. Nature Cell Biology 2013).

Fig5LMN: Fig5L shows elevated uniporter components and GHITM in 10 mth old TMEM65 ko muscle. Fog Fig5MN, legend indicates that data are from 10 mth old mice; however, there is no evidence of elevated MCU and GHITM in the wb (panel MN). What is the reason for this discrepancy?

We thank the reviewer for catching this error. The data in Figure 5m,n is from two month old animals where there is no difference in calcium handling components other than Tmem65. This has now been corrected in the figure legend and the age has been added to the figure panel as suggested by the reviewer above.

Fig5M: NCLX wb. Why at 25kDa. In Palty et al 2004, skel muscle band is shown at ~50 kDa. Palty et al show a band at ~35 kDa for heart, but heart also shows a (slightly stronger) band at ~50 kDa, as for skel muscle. At the very least, you should show a membrane for MW spanning ~65 down to 20 kDa, for readers to make their own assessment.

We thank the reviewer for this comment. The blot for the full gel is shown in Supplemental Figure 4.

Figure 6
Panel K, vs. Extended data Fig7B – what can account for the decreased CSA in KO muscle shown in EDFig7B vs. the increase shown in Fig6K? More generally, I have a concern with the interpretation of some of the double KO data in Fig6: data in Fig6K and also in Fig6J suggests that MCU KO has a predominant phenotype that is not rescued by TMEM65 KO. That would be my interpretation. I would not say that these data are revealing something primarily about TMEM65.

We agree with the reviewer that the MCU phenotype for muscle fiber type (slow→fast) is stronger than the Tmem65 phenotype (fast→slow). Thus, in the double KO, the MCU phenotype predominates and therefore rescues the loss of type IIa fibers observed in Tmem65 KO mice.

Where are data from TMEM65-/-::MCU+/+ muscle? I think that that would be more informative. I don't think the data should be omitted. But, perhaps these data could be relocated to the supplement?

We thank the reviewer for this comment. TMEM65-/-::MCU+/+ mice die at ~20 days of

age and thus cannot be included in analyses of mature muscles as shown for Figure 6e-l. Since the fiber type and metabolic phenotypes were only present in mature Tmem65 KO muscles, the MCU KO phenotype rescue experiments also needed to be done in mature muscles.
